# n-Tuples on Scaffold Diversity Inspired by Drug Hybridisation to Enhance Drugability: Application to Cytarabine

**DOI:** 10.3390/md21120637

**Published:** 2023-12-13

**Authors:** Miguel García-Castro, David Fuentes-Rios, J. Manuel López-Romero, Antonio Romero, Federico Moya-Utrera, Amelia Díaz-Morilla, Francisco Sarabia

**Affiliations:** Department of Organic Chemistry, Faculty of Sciences, University of Malaga, Campus de Teatinos s/n, 29071 Malaga, Spain

**Keywords:** scaffold diversity, dual drugs, chemical space, arabinonucleosides, n-duples

## Abstract

A mathematical concept, n-tuples are originally applied to medicinal chemistry, especially with the creation of scaffold diversity inspired by the hybridisation of different commercial drugs with cytarabine, a synthetic arabinonucleoside derived from two marine natural products, spongouridine and spongothymidine. The new methodology explores the virtual chemical-factorial combination of different commercial drugs (immunosuppressant, antibiotic, antiemetic, anti-inflammatory, and anticancer) with the anticancer drug cytarabine. Real chemical combinations were designed and synthesised for 8-duples, obtaining a small representative library of interesting organic molecules to be biologically tested as proof of concept. The synthesised library contains classical molecular properties regarding the Lipinski rules and/or beyond rules of five (bRo5) and is represented by the covalent combination of the anticancer drug cytarabine with ibuprofen, flurbiprofen, folic acid, sulfasalazine, ciprofloxacin, bortezomib, and methotrexate. The insertion of specific nomenclature could be implemented into artificial intelligence algorithms in order to enhance the efficiency of drug-hunting programs. The novel methodology has proven useful for the straightforward synthesis of most of the theoretically proposed duples and, in principle, could be extended to any other central drug.

## 1. Introduction

Scaffold diversity is one of the most desired characteristics for the hunting potential of drugs in scientific programs directed at biologically testing chemical libraries. Restrictions regarding structural diversity in a compound library limit the discovery of small bioactive molecules for medicinal chemistry and chemical biology research [1]. Thus, the leading discovery technologies require the design of libraries for screening, and those libraries must be as rich in scaffold diversity as possible in order to enable the exploration of unexplored chemical space [2]. The concept of molecular diversity has been introduced to maximise the coverage of structural space with the hope of minimising the redundancy provided by biological screening [3]. Several strategies have been developed during the last two decades to address this topic, incorporating imaginative and creative tools for medicinal chemistry programs that have provided a good arsenal of hits and leads for candidates for future drugs [1,3,4,5,6,7].

**Figure 1:**
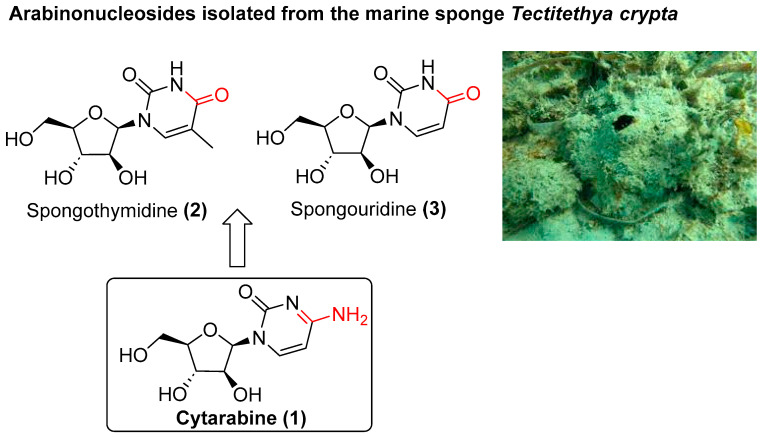
Chemical structures of cytarabine (**1**) and natural marine arabinonucleosides.

Our experience in the synthesis of organic molecules with important biological activities has inspired us to pay special attention to those that are natural products and their derivatives. Thus, cytarabine (**1**) drew our attention because it is one of the most important anticancer drugs administrated for the treatment of acute myeloid leukaemia (AML) [8,9]. Cytarabine (**1**) is a synthetic pyrimidine nucleoside containing a cytosine base derived from a specific program based on the natural marine products spongothymidine (**2**) and spongouridine (**3**), which are isolated from *Cryptotethya crypta* (now renamed *Tectitethya crypta*) [10,11] (Figure 1). Since its approval by the FDA in 1969, some efforts have been made to synthesise analogues of cytarabine [12,13,14,15] and, more recently, in the administration of delivery systems [16,17]. However, to date, no synthetic program has been directed towards the development of molecular diversity with cytarabine as the central scaffold.

A central aim of this work is to transfer the mathematical concept of n-uples (also named tuples) to chemistry, particularly for scaffold diversity generation. In mathematics, an n-uple (or tuple) is a finite ordered sequence of n-elements, *n* being a non-negative integer number. Furthermore, once an uple (or tuple) is generated, it cannot be modified or altered [18,19]. Taking into account this fact, we originally applied this concept for the generation of scaffold diversity in a compounds library that pivoted on cytarabine (**1**), although the general principle could be applied to whatever drug possesses specific binding sites, as will be defined in the results and discussion.

We have taken into consideration the recent antecedents that exist in the literature regarding the combination of two drugs by covalent binding, more specifically, in the area of drug delivery using nanotechnology. In this way, several examples have been found to be representative of dual drugs backboned for synergistic biologic effects, which is especially important in chemotherapy [20,21,22,23]. Nevertheless, all these investigations have been addressed to enhance the potency of chemotherapy for the improvement of the pharmacokinetics; thus, the employed drugs are dual or triple combinations of the same kind of drug (the anticancer drugs library, e.g., gemcitabine plus cisplatin, mitoxantrone plus curcumin, etc.). Especially relevant is the synthesis of novel liver-specific cholic-acid-cytarabine conjugates with potent antitumor activities achieved by Shen’s group [24]. Furthermore, some other cytarabine conjugates have been described as antineoplastics [25].

Herein, we describe a novel methodology for the generation of scaffold diversity in a chemical library via the introduction of the mathematical concept of n-tuples, which are organic molecules that bond the anticancer drug cytarabine (**1**) covalently to different classes of commercial drugs (antibiotics, antiemetics, immunosuppressants, and anti-inflammatory agents). The aim of this strategy lies in the fact that dual actions and/or synergistic therapeutic effects can be expected. In some specific cases, as for those duples based on the hybridisation of anticancer drugs (cytarabine-methotrexate and cytarabine-bortezomib), an enhancement of antitumor potency would be desirable. Additionally, the new scaffolds could explore the chemical space, looking for new biological targets that could be involved in many other diseases.

## 2. Results and Discussion

### 2.1. Nomenclature Foundations

**Figure 2:**
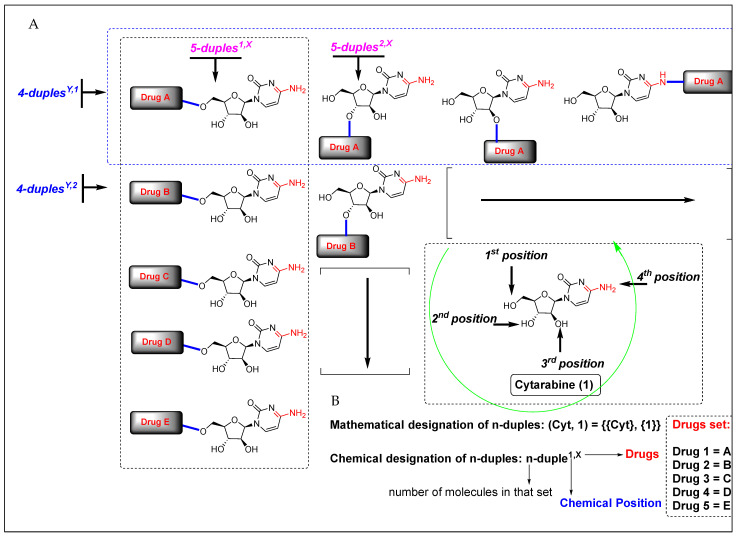
(**A**) Virtual generation of diverse n-duples systems with cytarabine. (**B**) Mathematical and chemical nomenclature for n-duples on scaffold diversity chemical libraries.

**Figure 3:**
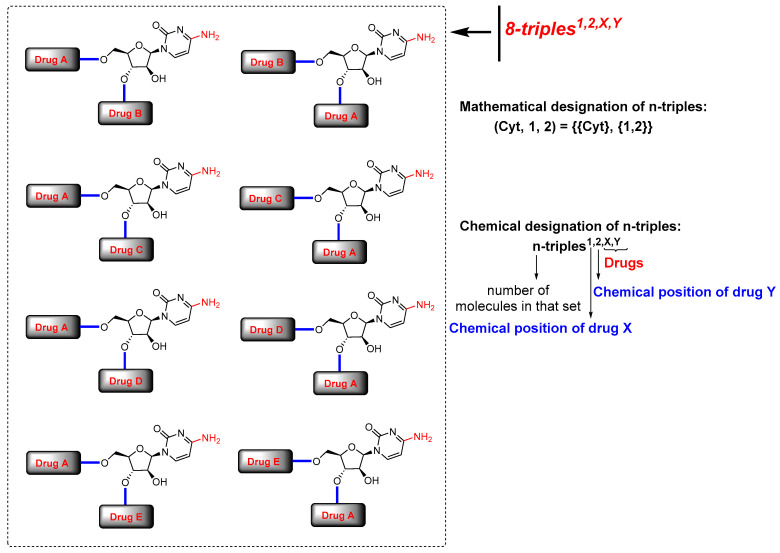
Virtual generation of diverse n-triples systems with cytarabine. Mathematical and chemical nomenclature for n-triples in scaffold diversity chemical libraries.

Considering the chemical structure of cytarabine (**1**), which contains the carbohydrate 1-*β*-D-arabinofuranose and possesses three potential binding sites occupied by three hydroxyl groups, and the cytosine framework, which contains an amine group, we counted four binding sites in total. Therefore, potentially and theoretically, we could attach up to four drugs to cytarabine. Nevertheless, we are interested in creating virtual chemical libraries based on n-duples (Figure 2); however, in principle, we could generate even n-triples and n-quadruples libraries containing an important level of structural diversity (Figure 3).

As we describe in Figure 2 and Figure 3, we first created a systematic nomenclature for sets of molecules following the rules of mathematics. In this way, a set of elements must be named using brackets and employing commas for the separation of elements. Next, square brackets separate every element in the set, as we have written in the examples below. Secondly, we adopted a chemical nomenclature for naming n-duple^number,number^. It is worth noting that the mathematical nomenclature of a set of molecules is a broader nomenclature than the chemical one; however, chemical nomenclature allows naming different kinds of duples according to two criteria: commercial drug or binding position and the naming of a particular element in a set. For example, 7-duples^1,X^ is a set of seven molecules where cytarabine is covalently bonded in position 1 with seven different commercial drugs, represented by X (where X can be numbered with 1, 2, 3…). Inside 7-duples^1,X^, the element named duple^1,1^ is a particular compound where cytarabine is covalently bonded in position 1 to drug 1. More concretely, once a duple system is fixed on a central drug, we can translate the general name of the duple with the acronym name of the central drug. In our case, n-duples^4,X^ would adopt the final name n-Cyt^4,X^, where Cyt is the acronym of cytarabine (Table 1, and Appendix A). This specific chemical nomenclature will make more sense for indexing in any future database containing many duples built with a multitude of central drugs.

Since the criterion for chemical position numbers could be assessed arbitrarily, we have decided to assign them in increasing order of the electronegativities of the involved elements in the covalent bond. Thus, the oxygen atom belonging to the hydroxyl group must be numbered first, and the nitrogen atom belonging to the amine group must be numbered later. Because of this criterion, the alcohol functional groups are numbered with priority over the amine groups, receiving numbers 1, 2, and 3 (counter-clockwise), and the number 4 is assigned to the amine group of cytarabine (Figure 2, green arrow).

The intention of establishing this nomenclature goes further than the simple introduction of names; it will allow implementation in algorithmic systems used by artificial intelligence programs to check the data collection for libraries of compounds. Thus, this kind of search would allow researchers to know which combinations of drugs have already been synthesised and, accordingly, their exhibited biological properties. In combination with different programs based on artificial intelligence, this methodology could enhance the hunting of new hits and leads in medicinal chemistry. As Lewis, Luksch, and Reker state, “analysis of very big chemical datasets is a major research area that can profit from the application of modern machine learning and AI-based methods” [26,27].

### 2.2. Chemistry

#### 2.2.1. Selection of Commercial Drugs

Once we established the criteria for the classification of chemical libraries adopting the mathematical concept of n-tuples and having also introduced the chemical nomenclature of sets of molecules and individual molecules in the set, we selected the set of drugs to be combined with our central drug, cytarabine. In Table 2, we propose suitable commercial drugs for the treatment of diverse diseases. Virtually, we can generate 16-duples with cytarabine as the central drug, resulting in 16 compounds. If we consider the four available attachment positions in cytarabine, up to 64-duples can be built (Table 3). Among these drugs, we selected a set of ten commercial drugs with important and diverse biological activities that intentionally contain a functional group amenable to attachment to cytarabine. From a chemical point of view, we have mainly selected acid groups because the coupling reaction would involve a robust esterification or amidation reaction, although amine groups can also be selected because they can be attached via a bimolecular nucleophilic substitution reaction with the corresponding prepared substrate. Nonetheless, from a therapeutic point of view, the selection of drugs was performed to achieve either a dual effect or synergy against the disease itself. Therefore, we have chosen anti-inflammatory, immunosuppressant, antibiotic, antiemetic, and anticancer drugs. Representative commercial molecules of these types of drugs are ibuprofen, sulfasalazine, ciprofloxacin, metoclopramide, bortezomib, etc.

Our therapeutic endpoint expectations regarding the use of these commercial drugs are based on known scientific literature. For instance, numerous articles relate inflammatory processes with a possible cause of cancer proliferation [28,29,30] or even with a possible origin [31,32]. Likewise, folic acid targets cancer cells because specific receptors for this vitamin are present on their lipid membranes [33,34,35,36]. The relationship between antibiotics and cancer is also well documented [37,38,39], even becoming a paradigmatic issue [40]. Lastly, the ability of some immunosuppressors, such as everolimus or rapamycin, to limit oncogenesis among organ recipients is also known [41]. 

**Figure 4:**
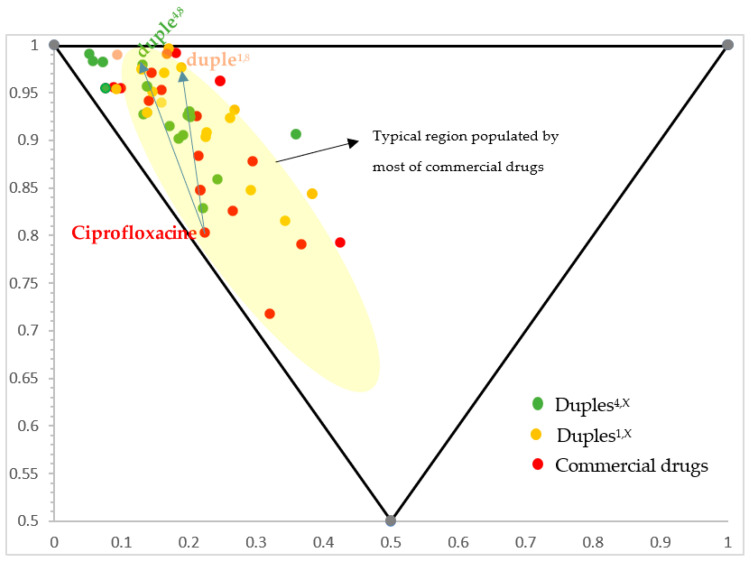
Representation of scaffold diversity according to PMI analysis of duples versus individual commercial drugs. For example, blue arrows indicate the movement from free ciprofloxacin to duple^4,8^ (amide bond with cytarabine) and duple^1,8^ (ester bond with cytarabine).

Furthermore, for our selection of commercial drugs, we considered that the resultant organic drug-like substance follows the extended Lipinski rules Ro5 or those known as the beyond rules of five (bRo5) [42] regarding the physicochemical properties that every potential drug should have [43]. Therefore, our proposed 16-duples must follow the typical restrictions for ADMET (absorption, distribution, metabolism, excretion, and toxicity) indicated by those ’beyond rules of five’ while considering the possibility of hunting undruggable targets. The pharmaceutical opportunity for ’beyond rule of five drugs’ in next-generation therapeutics to target larger biological binding sites not accessible to conventional small molecules was established by Lokey’s group [42]. Likewise, normalised principal moments of inertia (PMI) were also performed to compare their three-dimensional shape diversity (Figure 4) (PMI was calculated using ChembioOffice v.22.2.0.3300 software), [44] and Material and Methods. As depicted in Figure 4, our 16-duples library is highly structurally diverse, and all designed duples are located in the same chemical space explored for all commercial drugs.

If we compare the normalised principal ratios of commercial drugs with their corresponding normalised principal ratios in duples, we can observe that the dots in the triangle move but are maintained inside the classical region populated by commercial drugs (the yellow zone; comparison between green versus red dots, respectively, in Figure 4), which is quite interesting from the point of view of chemical space exploration. Therefore, all our duples settle in the typical region where most commercial drugs are.

#### 2.2.2. Syntheses of Selected Duples

**Figure 5:**
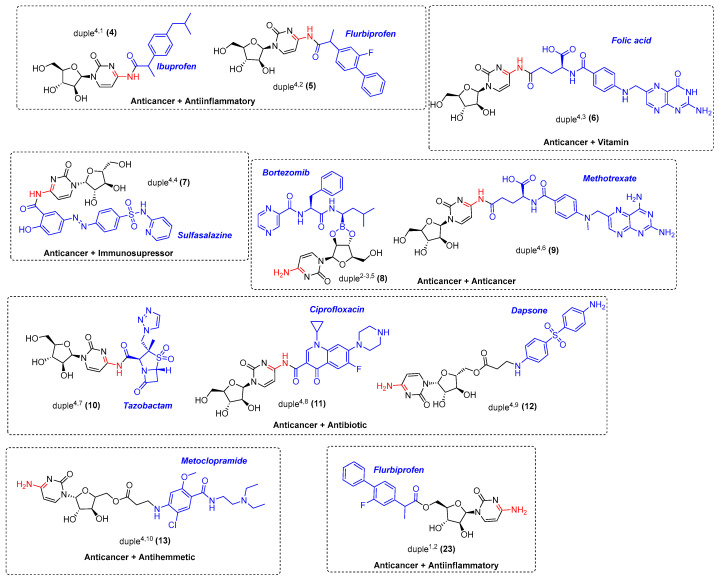
9-duples^4,X^, duple^2−3,5^, and duple^1,2^ were selected to be synthesised as proof of concept.

**Scheme 1:**
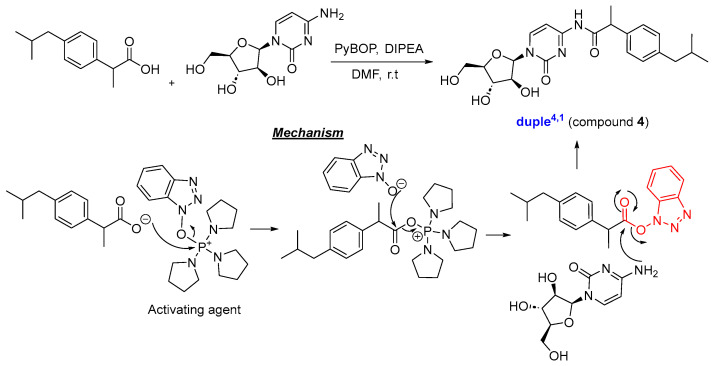
Synthesis of duple^4,1.^

Considering all these details, we embarked on synthesising the 11-duples depicted in Figure 5. Having established the guidelines for the systematic creation of scaffold diversity by designing our 16-duples with cytarabine and commercial drugs, we started our construction of 9-duples^4,X^, duple^1,2^, and a special duple^2−3,5^ as a proof of concept (Appendix A). For that purpose, we initiated the synthesis of duple^4,1^ (compound **4**), consisting of hybridisation with ibuprofen, following the standard procedure for the amidation reaction, which employs activating agents such as DCC and DMAP, EDCI, and HATU or by using PyBOP and DIPEA, and stirring for days at room temperature [45]. Compound **4** was then obtained in 42% yield (Scheme 1). The same protocols were systematically used for the syntheses of: duple^4,2^ (compound **5**), consisting of hybridisation with flurbiprofen; duple^4,3^ (compound **6**), consisting of hybridisation with folic acid; duple^4,4^ (compound **7**), consisting of hybridisation with sulfasalazine; duple^4,6^ (compound **9**), consisting of hybridisation with methotrexate; and duple^4,8^ (compound **11**), consisting of hybridisation with ciprofloxacin. Yields of those pure compounds ranged from 20–40% (Figure 5). It is especially relevant that the straightforward syntheses, without using protecting groups, afforded the coupling between cytarabine and drugs through amide bonds (amidation reaction) versus ester bonds (esterification reaction). 

From the point of view of chemical synthesis, we must highlight the straightforward strategy raised that avoids the employment of protecting groups. Although the yields could be enhanced using the latter strategy, from the point of view of the pharmaceutical industry, shorter synthetic routes for both economic and environmental reasons are always preferred [46].

Unfortunately, the syntheses of some duples were unsuccessful, such as the planned duple^4,7^ (compound **10**), which consisted of hybridisation with the antibiotic tazobactam in all different protocols. We explained this negative output because of the strong acidic proton present in the carbapenem cycle, which, after basic treatment of DIPEA, may be abstracted, triggering the decomposition of tazobactam. 

As we have already stated, it is especially important that the coupling between both drugs occurs in the nitrogen atom of cytarabine (position 4). It is known that the amine group of cytarabine increases the degradation ability in the human metabolism by involving the degradation process of rapid deamination by the liver enzyme cytidine deaminase [42,47]. Because of this, several works have been directed towards the synthesis of cytarabine derivatives by blocking that position [48,49]. Our goal in this direction was also to block this position in duples, producing new molecules with two substructures with an important biological and therapeutic activity that could produce synergies regarding the therapeutic effect. The conjugate molecule breakdown should deliver two active molecular species. However, it cannot be excluded that the conjugate may possess distinct biological activity, as Novotny states [25]. Therefore, two diverse research lines could be started to corroborate these hypotheses. On the one hand, one might study in vitro hydrolysis employing human amidase enzymes. On the other hand, biological assays of our synthesised duples could be examined. Further investigation is currently being carried out in painting cell assays [a collaboration was established with COMAS centre at the Max Planck Institute of Molecular Physiology (Dortmund, Germany), headed by Dr. Sonja Sievers, a member of Prof. H. Waldmann’s group (Chemical Biology department)].

**Scheme 2:**
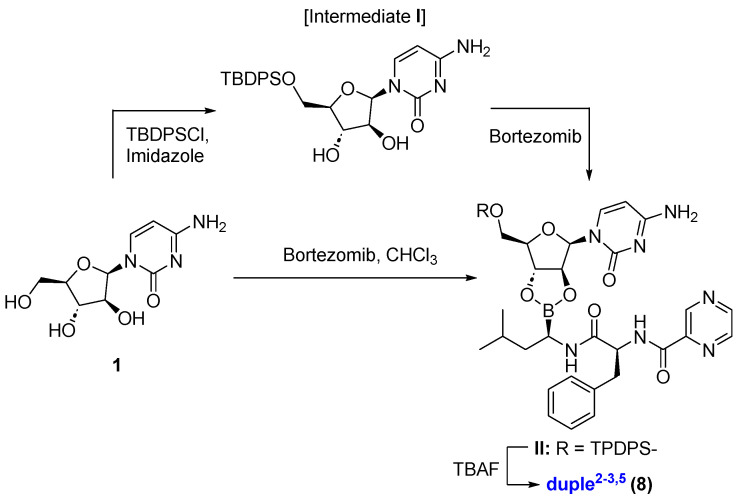
Synthesis of duple^2−3,5.^

For duple^2−3,5^ (compound **8**), we protected the primary alcohol, *tert*-butyldiphenylsilyl ether derivative (intermediate **I**) [50,51], and then accomplished the coupling with bortezomib, yielding, after deprotection of silylether, pure compound **8** in a 34% yield [52,53]. This duple^2−3,5^ is particularly interesting because it represents the only example where two positions on cytarabine are linked at the same time [See Supplementary Information for DFT calculations of duple^2−3,5^]. Alternatively, we also synthesised the duple^2−3,5^ in a straightforward manner without the need to employ protecting groups in an acceptable yield (42%), which represents a significant advantage for an efficient diversity-oriented synthesis strategy (Scheme 2). DFT calculations justify the most stable diol system (*anti*-position) to form the final compound **8**, among other possibilities (DFT calculations were performed using Gaussian software 2016 c_02_22, and Figure S2).

#### 2.2.3. Duples Using Linkers

**Scheme 3:**
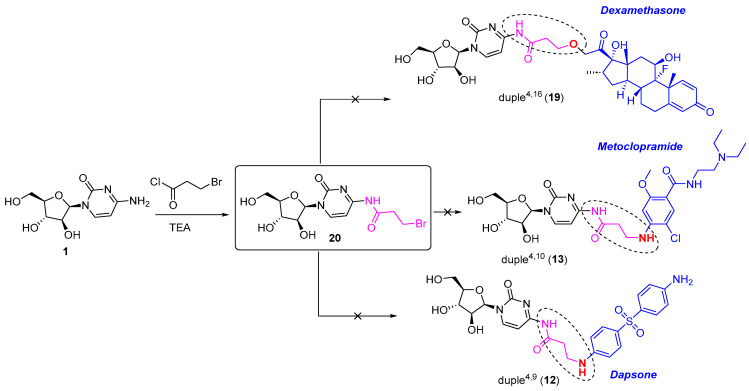
Unsuccessful syntheses of diverse duples^4,X^ through a linker.

Finally, all efforts devoted to introducing diverse commercial drugs through the amine position of the corresponding drug, as for duple^4,9^ (**12**) and duple^4,10^ (**13**), and through the ether position, as for duple^4,16^ (**19**), were unsuccessful. For this purpose, we needed to employ the linker 3-bromopropyl chloride, which was first attached to cytarabine to form the intermediate **20**. However, we could not isolate enough of the pure form of compound **20** (Scheme 3). We also tried to achieve the syntheses of these duples without the isolation of intermediate **20**, but none of those reactions provided the coveted duples (Scheme 3). Further similar studies are currently under investigation.

**Scheme 4:**
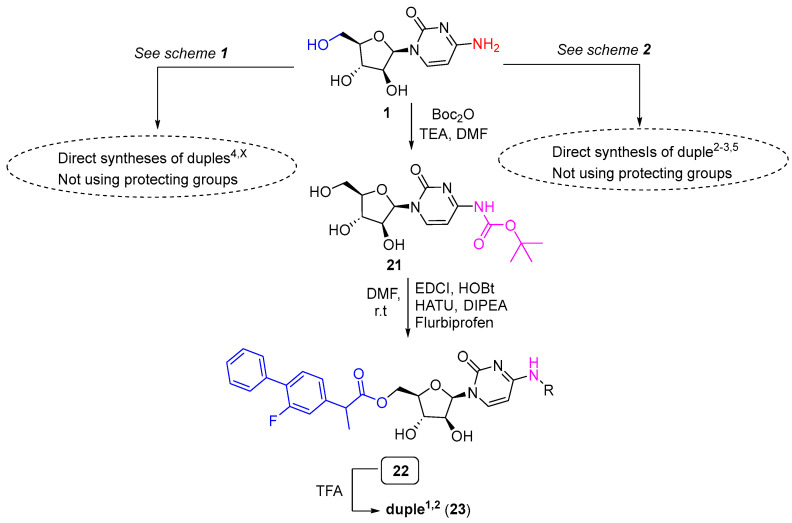
Modulation to access duples^1,X^.

Nevertheless, and particularly interesting, was the modulation to synthesise duples^1,X^ using a strategy consisting of the protection of cytarabine with a Boc group and the esterification between the free primary alcohol of cytarabine with the free carboxylic acid of the corresponding drug, e.g., flurbiprofen for duple^1,2^. This modulation allowed easy access to duples^1,X^ (Scheme 4). 

Thus, cytarabine (**1**) was protected by employing Boc anhydride in the presence of triethylamine to yield compound **21** (42% yield). Pure compound **21** was then coupled with flurbiprofen to get the Boc-duple^1,2^ (**22**). Deprotection of **22** using TFA provided duple^1,2^ (**23**) at a 94% yield.

Duples^1,X^ open an interesting discussion because these new molecules contain an ester function. In the literature, there are some excellent examples of the derivatisation of cytarabine at the primary alcohol protected by ester [54,55,56]. We believe that these ester derivatives could be hydrolysed in vivo due to the action of human esterases, liberating two drugs into the bloodstream. Our hypothesis is that a dual biological effect could be achieved. Further work will be addressed to create a library containing duples^1,X^ and investigate the hydrolysis with human esterases [57].

## 3. Materials and Methods

All commercial drugs and reagents were purchased from Sigma-Aldrich, except for cytarabine, which was acquired from Fluorochem. All reactions were carried out under an inert atmosphere (argon or nitrogen). Yields refer to chromatographically and spectroscopically using the proton nuclear magnetic resonance (^1^H-NMR) of homogeneous materials unless otherwise stated. All solutions used in the workup procedures were saturated unless otherwise noted. All coupling reagents, such as PyBOP, HOBt, EDCI, etc., were purchased from Sigma-Aldrich at ACS reagent quality. All reactions were monitored by thin-layer chromatography carried out on 0.25 mm silica gel plates (60F-254) using UV light as the visualising agent and phosphomolybdic acid solution (PMA) and heat as the developing agents. Silica gel (60, particle size 0.040–0.063 mm) was used for flash column chromatography. Preparative thin-layer chromatography (PTLC) separations were carried out on 0.25-, 0.50-, or 1-mm silica gel plates (60F-254). Some products were purified using Biotage^®^ equipment (Isolera prime) and commercial silica-gel cartridge SFAR-DUO 10 g (60 µM particle size). Some products were purified using flash column chromatography using silica gel 60 (0.040–0.063 mm), 230–400 mesh ASTM.

Proton nuclear magnetic resonance (^1^H-NMR) spectra were recorded on Bruker 500 MHz or 400 MHz instruments and calibrated using residual undeuterated solvent as an internal reference. The following abbreviations were used to explain the multiplicities: s, singlet; d, doublet; t, triplet; q, quartet; m, multiplet; band, several overlapping signals; and b, broad. ^1^H-NMR assignments were undertaken based on bidimensional NMR experiments of COSY, HSQC, HMBC, and NOESY experiments. 

### 3.1. Mass Spectrometric Analysis

The dried droplet method was used to prepare the samples for MALDI analysis. Briefly, samples were mixed in an Eppendorf tube at a 1:1 ratio with 2,5-dihydroxybenzoic acid (DHB), α-Cyano-4-hydroxycinnamic acid (α-CHCA) matrix, or DITHRANOL matrix. Each matrix was prepared at a 15 mg/mL concentration and dissolved in TA50 (50% [*v*/*v*] acetonitrile, 0.1% [*v*/*v*] trifluoroacetic acid in distilled water). Then, a 2 μL volume of the sample-matrix mixture was spotted on a stainless-steel sample plate and allowed to dry for 10 min at room temperature. Mass spectra were recorded with an ultrafleXtreme^®^ matrix-assisted laser desorption ionisation-time-of-flight/time-of-flight (MALDI-TOF/TOF) mass spectrometer (Bruker Daltonics, Billerica, MA, USA) equipped with a nitrogen laser emitting at 337 nm and operated in reflectron positive mode with the flexControl software (version 3.4; Bruker Daltonics). The laser power was manually adjusted until the optimum signal-to-noise ratio was obtained, and each acquired spectrum resulted from the accumulation of a minimum of 2500 laser shots. Spectra were analysed using the Flex Analysis software (Bruker Daltonics).

### 3.2. Typical Protocol for Amidation

To a solution of a commercial acid drug (0.41 mmol, 1.0 equiv.) and benzotriazole-1-yl-oxy-tris-pyrrolidino-phosphonium hexafluorophosphate (PyBOP) (214 mg, 0.41 mmol, 1.0 equiv.) in dry DMF (15 mL), DIPEA (73 μL, 0.45 mmol, 1.1 equiv.) and cytarabine (100 mg, 0.41 mmol, 1.0 equiv.) were sequentially added, and the mixture was stirred at room temperature under an argon atmosphere for 18 h. Ethyl acetate (20 mL) and ammonium chloride saturated solutions were added to the reaction mixture. The extraction process was triplicated, and the organic phase was sequentially washed with distilled water and brine, dried over anhydrous magnesium sulphate, then filtered, and concentrated in the rotavapor. Purification was performed using Isolera Biotage^®^ with a gradient of eluent (from 1% to 10% methanol in DCM). 

### 3.3. Alternative Protocol for Amidation 

To a solution of a commercial acid drug (0.41 mmol, 1.0 equiv.) and 1-[Bis(dimethylamino)methylene]-1*H*-1,2,3-triazolo[4,5-*b*]pyridinium-3-oxid-hexafluorophosphate, HATU (312 mg, 0.82 mmol, 2.0 equiv.) and EDCI·HCl (79 mg, 0.41 mmol, 1.0 equiv.) in dry DMF, DIPEA (100 μL, 0.55 mmol, 1.33 equiv.), and cytarabine (100 mg, 0.41 mmol, 1.0 equiv.) were sequentially added and stirred for 24 h at room temperature.

Ethyl acetate (20 mL) and ammonium chloride saturated solution were added to the reaction mixture. The extraction process was triplicated, and the organic phase was sequentially washed with distilled water and brine, dried over anhydrous magnesium sulphate, filtered, and concentrated in the rotavapor. The crude was purified through flash column chromatography using a gradient of eluent (from methanol 1% to 10% in DCM). 

### 3.4. Protection of Cytarabine’s Primary Alcohol

In a 50 mL round bottom flask, 300 mg of cytarabine (1.23 mmol, 1.0 equiv.) was dissolved in 25 mL of DMF. To this solution, 117.23 mg of Imidazole (1.722 mmol, 1.4 equiv.) and 0.390 mL of TBDPSCl (1.476 mmol, 1.2 equiv.) were added by slow dropping at 0 °C. The reaction was stirred at ambient temperature for 24 h. The solution was dissolved in DCM and washed (3 times) with 100 mL of a saturated solution of NH_4_Cl. The organic phase was dried over anhydrous magnesium sulphate, filtered, and concentrated in the rotavapor, affording 557 mg of a white solid, a 93% yield.

### 3.5. Synthesis of Cytarabine-Bortezomib

In a 25 mL round bottom flask, 20.8 mg of cytarabine (0.085 mmol, 1.0 equiv.) was dissolved in 5 mL of anhydrous MeOH. After complete dissolution, 18.07 mg of bortezomib (0.047 mmol, 0.55 equiv.) was added to the solution. The coupling reaction mixture was stirred for 72 h. The residue upon workup was purified through flash column chromatography using silica gel, affording 11.9 mg of cytarabine-bortezomib, a 42% yield.

### 3.6. Protocol for Introduction of Linker and Further Bimolecular Nucleophilic Substitution

In a 100 mL round bottom flask, 300 mg of cytarabine (1.23 mmol, 1.0 equiv.) was dissolved in 50 mL of DMF. After complete dissolution, 0.136 mL of TEA (1.353 mmol, 1.1 equiv.) and 0.1886 mL of 3-bromopropionyl chloride (1.353 mmol, 1.1 equiv.) were added by slow dripping for 6 h at 0 °C. After this addition, the solution was left stirring at room temperature overnight. After the complete disappearance of the starting product, the reaction was quenched with 3 drops of saturated NaHCO_3_ at 0 °C, and the reaction crude was lyophilised. Subsequently, the residue upon workup was chromatographed on silica gel with DCM:MeOH (8:2), obtaining 156 mg of the desired product, a yield of 33%.

In a 50 mL round bottom flask, 54.6 mg of dapsone (0.264 mmol, 1.0 equiv.) was dissolved together with 4 Å molecular sieves (100 mg). Lithium hydroxide (5.3 mg, 0.22 mmol, 1.0 equiv.) was added to this solution and stirred at room temperature for 30 min. After this period, 100 mg of cytarabine-chain (0.264 mmol, 1.2 equiv.) was added. The reaction was stirred at ambient temperature for 72 h with an unsuccessful result.

### 3.7. Typical Protocol for Esterification

Cytarabine (500 mg, 2.06 mmol, 1.0 equiv.) was dissolved in DMF (50 mL). *Tert*-butyloxycarbonyl anhydride (538 mg, 2.47 mmol, 1.2 equiv.) was then added, and the solution was stirred for 24 h at ambient temperature. After the typical workup, the crude was purified through flash column chromatography using MeOH: DCM (1:9) to afford 302 mg of Boc-protected cytarabine (**21**). 

Compound **21** (40 mg, 0.12 mmol, 1.0 equiv.) was dissolved in DMF (20 mL), and then EDCI (31 mg, 0.20 mmol, 1.6 equiv.), HATU (76 mg, 0.20 mmol, 1.6 equiv.), HOBt (27 mg, 0.20 mmol, 1.6 equiv.), and DIPEA (43 μL, 0.24 mmol, 2 equiv.) were sequentially added. The solution was stirred for 10 min. Then, an acidic commercial drug, for example, flurbiprofen (50 mg, 1.6 equiv.), was added, and the reaction mixture was stirred at room temperature overnight. Workup was realised using an extraction with ethyl acetate (3 times) and washing with an ammonium chloride saturated solution. Organic layers were sequentially washed with distilled water and brine and then dried over anhydrous magnesium sulphate, filtered, and concentrated in the rotavapor. Purification with flash column chromatography was realised using a gradient of methanol-dicloromethane as the eluent.

### 3.8. Principal Moments of Inertia (PMI)

PMI analysis was performed using Chembioffice 3D software (v22.0.0.22) after minimisation of energy using a Forcefield MMFF94; eps = r, Cutoff [8,10] and Gradient = 0.1 RMS Kcal/mol/A^2^. The principal moment of inertia and related calculations were performed in units of Dalton (AMU) and Angstroms (Å). The stochastic conformational search algorithm in the Chem3D software package was used to generate 3D conformers for each compound. Sampling and minimisation parameters were implemented as follows: Stochastic Search Limit: 7; Refinement Conformation Limit: 300; Stochastic Search Failure Limit: 100; Stochastic Search Iteration Limit: 1000; Energy Minimisation Iteration Limit: 200; and Energy Minimisation Gradient Test: 0.01. Normalised PMI ratios (I1/I3 and I2/I3) of these conformers were obtained from Chem3D and then plotted on a triangular graph, with the coordinates (0,1), (0.5,0.5), and (1,1) representing a perfect rod, disc, and sphere, respectively [44]. 

## 4. Conclusions

We originally transferred the mathematical concept of n-tuples to a medicinal chemistry program in order to create scaffold diversity in small libraries. We generated a specific nomenclature for this set of molecules, which will be able to be implemented in informatics algorithms for AI software. Furthermore, the objective of this strategy was to enhance the drugability of cytarabine and to explore the chemical space to hunt biological binding sites not accessible to conventional small molecules. Thus, we designed the generation of virtual 16-duples with cytarabine as the central drug and diverse commercial drugs, including antibiotics, antiemetics, anticancer drugs, vitamins, anti-inflammatories, immunosuppressants, and so on. As proof of concept, we efficiently synthesised a part of this library, represented by 6-duples^4,X^ (compounds **4**–**11**), duple^2−3,5^ (compound **8**), and duple^1,2^ (compound **23**), in moderate to good yields in a straightforward manner without using protecting groups (with the exception of compound **23**). It is presumed that the present methodology may be extended and applied in the near future to different central commercial drugs to create scaffold diversity in pharmaceutical libraries. Despite the biological assays of this small library, which will provide the first validation of the present methodology, we present that all 8-duples herein efficiently synthesised and described achieved the ´beyond rules of five, bRo5´ for oral bioavailability. 

This article initiates the syntheses of diverse and multiple n-duples with cytarabine as the central drug. Further work will be directed towards the biological testing of the 6-duples^4,X^, duple^2−3,5^, and duple^1,2^, which were synthesised in this article and are currently under investigation. Furthermore, an interesting field of investigation is the possibility of a full enzymatic study with amidases in order to corroborate our hypothesis that these created duples could be hydrolysed in a physiological environment, delivering the two drugs independently.

Conceptually, we intentionally and successfully introduced a new methodology in the field of chemical biology through the synthesis of a small library based on a central drug, the anticancer drug cytarabine, with high scaffold diversity as the main feature. The covalent conjugation with diverse commercial and important drugs harbours the hope of finding dual actions and/or synergistic therapeutic effects. 

## Figures and Tables

**Table 1:** Nomenclature foundations. General, specific, and mathematical nomenclature.

General Chemical Nomenclature	Specific Chemical Nomenclature	Mathematical Nomenclature
n-duples^1,X^	n-Cyt^1,X^	(Cyt, 1) = {Cyt}, {Cyt, 1}; where Cyt = Central drug, in our case cytarabine, which is covalently bonded in position 1 to drug X (drug number).
n-duples^2,X^	n-Cyt^2,X^	(Cyt, 2) = {Cyt}, {Cyt, 2}; where Cyt = Central drug, in our case cytarabine, which is covalently bonded in position 2 to drug X (drug number).
n-duples^Y,1^	n-Cyt^Y,1^	It is included in diverse mathematical nomenclature. Drug 1 is attached to different positions in the central drug, in our case, cytarabine.

**Table 2:** Suitable commercial drugs for the treatment of diverse diseases.

Entry	Drug	Medical Uses	Main Functional Group ^1^
1	Ibuprofen	Anti-inflammatory	Carboxylic acid
2	Flurbiprofen	Anti-inflammatory	Carboxylic acid
3	Norfloxacin	Antibiotic	Carboxylic acid
4	Hydroxychloroquine	Immunosuppressor	Carboxylic acid
5	Metoclopramide	Antiemetic	Amine
6	Furosemide	Diuretic	Carboxylic acid
7	Cilastatin	Antibiotic	Carboxylic acid
8	Alprostadil	Erectile dysfunction	Carboxylic acid
9	Ciprofloxacin	Antibiotic	Carboxylic acid
10	Sulfasalazine	Immunosuppressor	Carboxylic acid
11	Methotrexate	Anticancer	Carboxylic acid
12	Tazobactam	Antibiotic	Carboxylic acid
13	Dexamethasone	Steroid	Alcohol
14	Folic acid	Vitamin	Carboxylic acid
15	Dapsone	Antibiotic	Amine
16	Bortezomib	Anticancer	Boronic acid

^1^ Functional group where it is attached cytarabine in the construction of our 16-duples.

**Table 3:** Virtual 16-duples built with cytarabine. Up to 64-duples can be achieved.

Entry	Drug	Substitution Positions ^1^	Lipinski Rules? ^2,3^
1	Ibuprofen	1; 2; 3; 4	Ro5
2	Flurbiprofen	1; 2; 3; 4	Ro5
3	Norfloxacin	1; 2; 3; 4	bRo5
4	Hydroxychloroquine	1; 2; 3; 4	Ro5
5	Metoclopramide	1; 2; 3; 4	bRo5
6	Furosemide	1; 2; 3; 4	bRo5
7	Cilastatin	1; 2; 3; 4	bRo5
8	Alprostadil	1; 2; 3; 4	bRo5
9	Ciprofloxacin	1; 2; 3; 4	bRo5
10	Sulfasalazine	1; 2; 3; 4	bRo5
11	Methotrexate	1; 2; 3; 4	bRo5
12	Tazobactam	1; 2; 3; 4	bRo5
13	Dexamethasone	1; 2; 3; 4	bRo5
14	Folic acid	1; 2; 3; 4	bRo5
15	Dapsone	1; 2; 3; 4	Ro5
16	Bortezomib	1; 2; 3; 4	bRo5

^1^ Substitution positions in cytarabine according to Figure 2. ^2^ Do resulting molecules follow the Lipinski rules Ro5 or bRo5? ^3^ See Supplementary Information for calculations and data.

## Data Availability

Data are contained within the article and Supplementary Material. The data presented in this study are available.

## Supplementary Materials

The following supporting information can be downloaded at: https://www.mdpi.com/article/10.3390/md21120637/s1, including all NMR-spectra and MALDI-TOFs of duples. Calculations regarding Lipinski Rules Ro5 and bRo5 for all virtual 16-duples. Calculations of PMI and NPR1, NPR2. Characterisation of all new compounds [58,59].

## Appendix A

### Drugs Set Designation Numbers in Duples Nomenclature

Ibuprofen = drug number **1**; Flurbiprofen = drug number **2**; Folic acid = drug number **3**; Sulfasalazine = drug number **4**; Bortezomib = drug number **5**; Methotrexate = drug number **6**; Tazobactam = drug number **7**; Ciprofloxacin = drug number **8**; Dapsone = drug number **9**; Metoclopramide = drug number **10**; Hydroxychloroquine = drug number = **11**; Norfloxacin = drug number **12**; Furosemide = drug number **13**; Cilastatin = drug number **14**; Alprostadil = drug number **15**; Dexamethasone = drug number **16**.

**Table A1:** General chemical nomenclature of our 16-duples.

[Compound **4**]:	Ibuprofen bond in position 4	=	duple^4,1^
[Compound **5**]:	Flurbiprofen bond in position 4	=	duple^4,2^
[Compound **6**]:	Folic acid bond in position 4	=	duple^4,3^
[Compound **7**]:	Sulfasalazine bond in position 4	=	duple^4,4^
[Compound **8**]:	Bortezomib bond in positions 2 and 3	=	duple^2−3,5^
[Compound **9**]:	Methotrexate bond in position 4	=	duple^4,6^
[Compound **10**]:	Tazobactam bond in position 4	=	duple^4,7^
[Compound **11**]:	Ciprofloxacin bond in position 4	=	duple^4,8^
[Compound **12**]:	Dapsone bond in position 4	=	duple^4,9^
[Compound **13**]:	Metoclopramide bond in position 4	=	duple^4,10^
[Compound **14**]:	Hydroxychloroquine bond in position 4	=	duple^4,11^
[Compound **15**]:	Norfloxacin bond in position 4	=	duple^4,12^
[Compound **16**]:	Furosemide bond in position 4	=	duple^4,13^
[Compound **17**]:	Cilastatin bond in position 4	=	duple^4,14^
[Compound **18**]:	Alprostadil bond in position 4	=	duple^4,15^
[Compound **19**]:	Desamethasone bond in position 4	=	duple^4,16^
[Compound **23**]:	Flurbiprofen bond in position 1	=	duple^1,2^

**Table A2:** Specific chemical nomenclature of our 16-duples.

[Compound **4**]:	Ibuprofen bond in position 4	=	Cyt^4,1^
[Compound **5**]:	Flurbiprofen bond in position 4	=	Cyt^4,2^
[Compound **6**]:	Folic acid bond in position 4	=	Cyt^4,3^
[Compound **7**]:	Sulfasalazine bond in position 4	=	Cyt^4,4^
[Compound **8**]:	Bortezomib bond in positions 2 and 3	=	Cyt^2−3,5^
[Compound **9**]:	Methotrexate bond in position 4	=	Cyt^4,6^
[Compound **10**]:	Tazobactam bond in position 4	=	Cyt^4,7^
[Compound **11**]:	Ciprofloxacin bond in position 4	=	Cyt^4,8^
[Compound **12**]:	Dapsone bond in position 4	=	Cyt^4,9^
[Compound **13**]:	Metoclopramide bond in position 4	=	Cyt^4,10^
[Compound **14**]:	Hydroxychloroquine bond in position 4	=	Cyt^4,11^
[Compound **15**]:	Norfloxacin bond in position 4	=	Cyt^4,12^
[Compound **16**]:	Furosemide bond in position 4	=	Cyt^4,13^
[Compound **17**]:	Cilastatin bond in position 4	=	Cyt^4,14^
[Compound **18**]:	Alprostadil bond in position 4	=	Cyt^4,15^
[Compound **19**]:	Desamethasone bond in position 4	=	Cyt^4,16^
[Compound **23**]:	Flurbiprofen bond in position 1	=	Cyt^1,2^
