# Supplementary material for: n-Tuples on Scaffold Diversity Inspired by Drug Hybridisation to Enhance Drugability: Application to Cytarabine
Source: Mar Drugs. 2023 Dec 13;21(12):637. doi: 10.3390/md21120637 (PMC10744741; doi:10.3390/md21120637)
Supplement: Supplementary file 1 [file marinedrugs-21-00637-s001.zip › marinedrugs-2687369-supplementary.pdf]

Figure S1. MOLECULAR PROPIERTIES OF 17-DUPLES AND LIPINSKI RULES OF FIVE (Ro5) OR BEYOND RULES OF FIVE (bRo5).

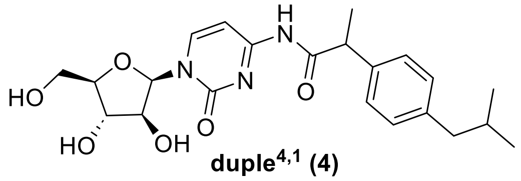

duple^{4,1} (4)

Chemical Formula: $C_{22}H_{29}N_3O_6$
Molecular Weight: 431,4890
tPSA: 131.69
CLogP: 1.76745
LogS: -3.043

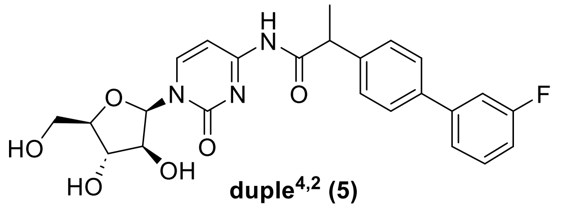

duple^{4,2} (5)

Chemical Formula: $C_{24}H_{24}FN_3O_6$
Molecular Weight: 469,4694
tPSA: 131.69
CLogP: 1.84245
LogS: -3.852

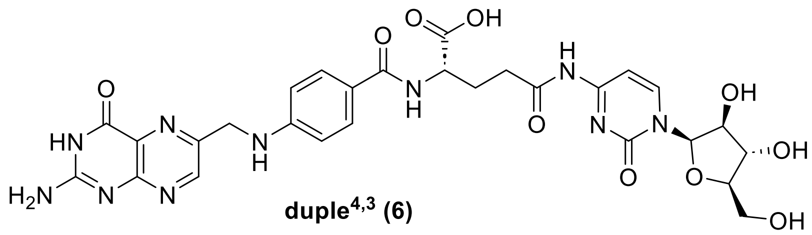

duple^{4,3} (6)

Chemical Formula: $C_{28}H_{30}N_{10}O_{10}$
Molecular Weight: 666,6080
tPSA: 302.32
CLogP: -4.21765
LogS: -4.12

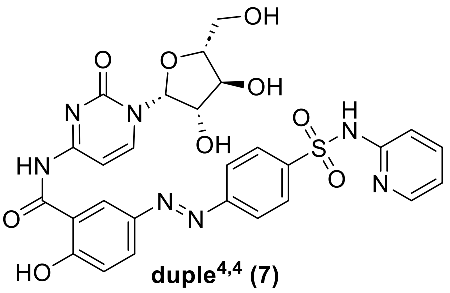

duple^{4,4} (7)

Chemical Formula: $C_{27}H_{25}N_7O_9S$
Molecular Weight: 623,5970
tPSA: 235.17
CLogP: 1.86609
LogS: -6.135

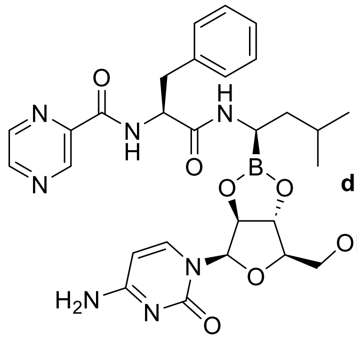

duple$^{2\text{-}3,5}$ (8)

Chemical Formula: $C_{28}H_{34}BN_7O_7$
Molecular Weight: 591,4320
tPSA: 189.53
CLogP: 0.48151
LogS: -4.922

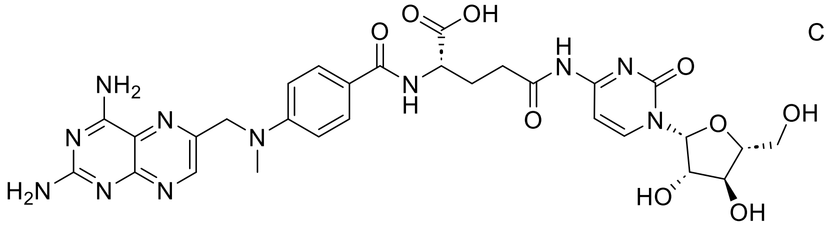

Chemical Formula: $C_{29}H_{33}N_{11}O_9$
Molecular Weight: 679,6510
tPSA: 302.81
CLogP: -2.37076
LogS: -4.374

duple^{4,6} (9)

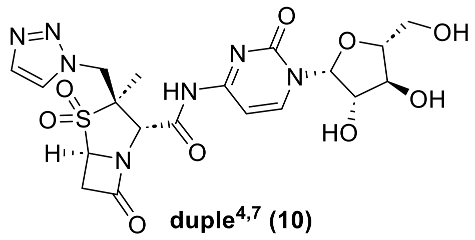

duple[4,7] (10)

Chemical Formula: $C_{19}H_{23}N_7O_9S$
Molecular Weight: 525,4930
tPSA: 214.1
CLogP: -2.51505
LogS: -0.5855

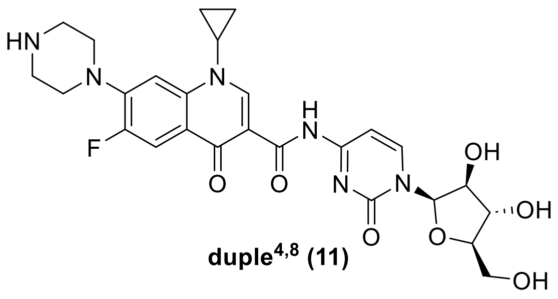

duple[4,8] (11)

Chemical Formula: $C_{26}H_{29}FN_6O_7$
Molecular Weight: 556,5514
tPSA: 167.27
CLogP: -1.46322
LogS: -3.589

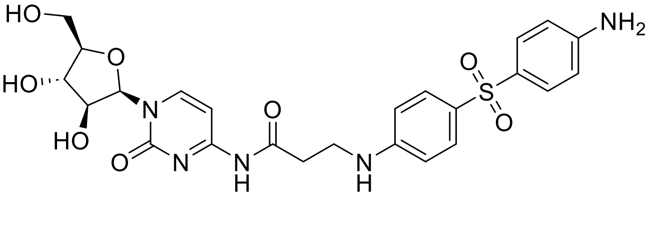

duple[4,9] (12)

Chemical Formula: $C_{24}H_{27}N_5O_8S$
Molecular Weight: 545,5670
tPSA: 203.88
CLogP: -0.401151
LogS: -3.213

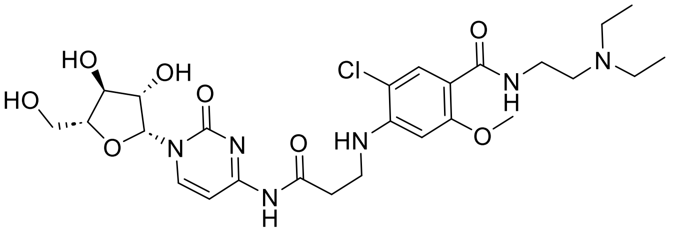

duple[4,10] (13)

Chemical Formula: $C_{26}H_{37}ClN_6O_8$
Molecular Weight: 597,0660
tPSA: 185.29
CLogP: 0.921321
LogS: -3.292

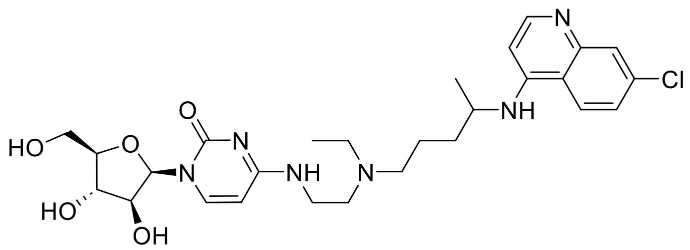

duple[4,11] (14)

Chemical Formula: $C_{27}H_{37}ClN_6O_5$
Molecular Weight: 561,0800
tPSA: 142.25
CLogP: 3.33853
LogS: -4.18

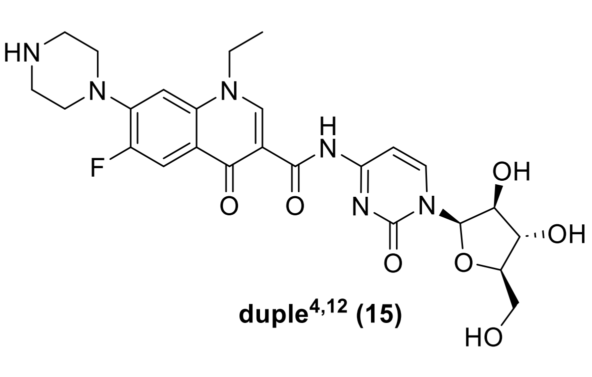

duple[4,12] (15)

Chemical Formula: $C_{25}H_{29}FN_6O_7$
Molecular Weight: 544,5404
tPSA: 167.27
CLogP: -1.51822
LogS: -3.393

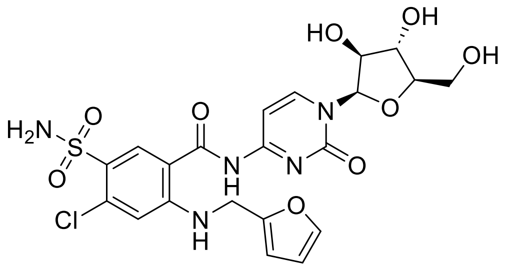

duple[4,13] (16)

Chemical Formula: $C_{21}H_{22}ClN_5O_9S$
Molecular Weight: 555,9430
tPSA: 213.11
CLogP: -0.791156
CMR: 12.9031
LogS: -3.496

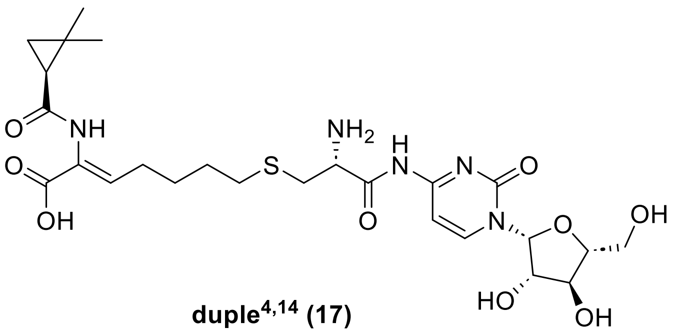

duple[4,14] (17)

Chemical Formula: $C_{25}H_{37}N_5O_9S$
Molecular Weight: 583,6570
tPSA: 224.11
CLogP: -2.24005
LogS: -2.637

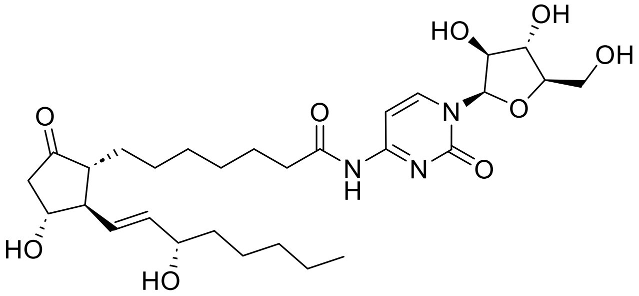

duple[4,15] (18)

Chemical Formula: $C_{29}H_{45}N_3O_9$
Molecular Weight: 579,6910
tPSA: 189.22
CLogP: 0.655847
LogS: -3.12

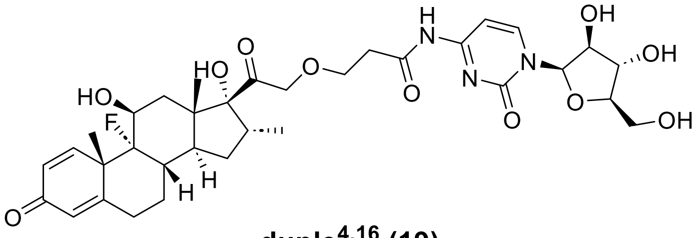

duple[4,16] (19)

Chemical Formula: $C_{34}H_{44}FN_3O_{11}$
Molecular Weight: 689,7344
tPSA: 215.52
CLogP: 0.207049
LogS: -2.522

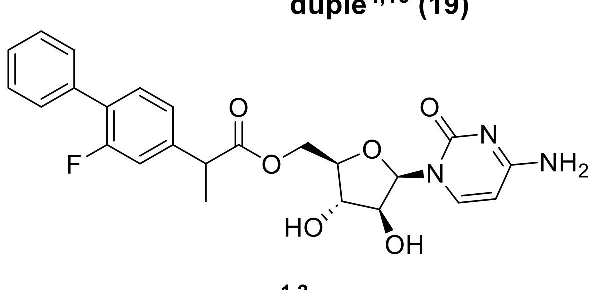

duple[1,2] (23)

Chemical Formula: $C_{24}H_{24}FN_3O_6$
Molecular Weight: 469,4694
tPSA: 134.68
CLogP: 2.01305
LogS: -4.307

Figure S2. DFT Minimized-3D structure for duple[2-3,5] (compound **8**).

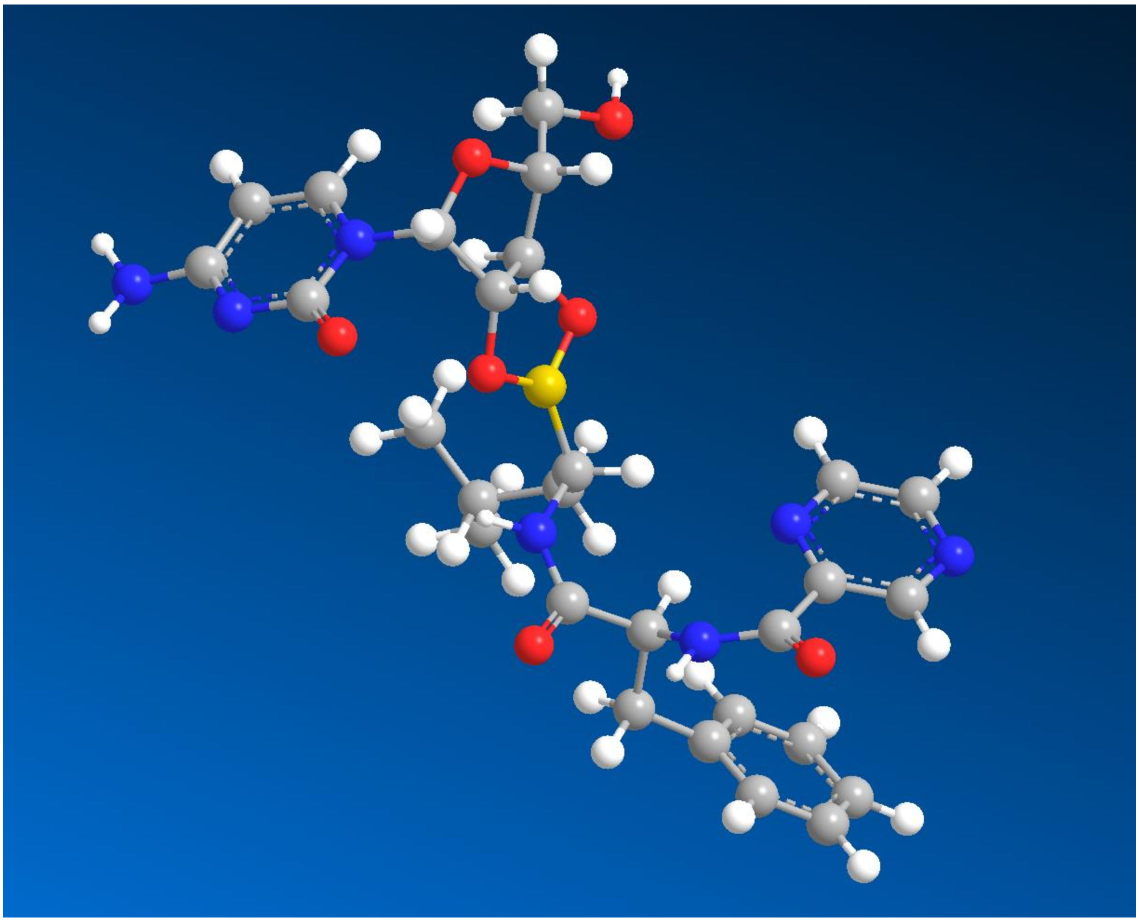

```
Calculation Type = FREQ
Calculation Method = RB3LYP
Basis Set = 6-31G(d)
Charge = 0
Spin = Singlet
Solvation = None
E(RB3LYP) = -2022.2077        Hartree
RMS Gradient Norm = 3.921e-06 Hartree/Bohr
Imaginary Freq = 0
Dipole Moment = 13.590484 Debye
Polarizability (?) = 352.57494 a.u.
Point Group = C1
Job cpu time:  0 days 16 hours 12 minutes 5.3 seconds.

Thermo Tab Data Section:
Imaginary Freq = 0
Temperature = 298.15 Kelvin
Pressure = 1 atm
Frequencies scaled by = 1
Electronic Energy (EE) = -2022.2077 Hartree
Zero-point Energy Correction = 0.624909 Hartree
Thermal Correction to Energy = 0.665292 Hartree
Thermal Correction to Enthalpy = 0.666237 Hartree
Thermal Correction to Free Energy = 0.544252 Hartree
EE + Zero-point Energy = -2021.5827 Hartree
EE + Thermal Energy Correction = -2021.5424 Hartree
```

```
EE + Thermal Enthalpy Correction = -2021.5414 Hartree
EE + Thermal Free Energy Correction = -2021.6634 Hartree
E (Thermal) = 417.477 kcal/mol
Heat Capacity (Cv) = 152.333 cal/mol-kelvin
Entropy (S) = 256.739 cal/mol-kelvin

Opt Tab Data Section:
Step number = 1
Maximum force = 1.5e-05 Converged
RMS force = 3e-06 Converged
Maximum displacement = 0.003079 Not converged
RMS displacement = 0.000622 Converged
Predicted energy change = -1.994309e-08 Hartree
```

Principal Moments of Inertia (PMI)[1] of 16-duples

Table S1. PMI for duples[4,X]

	npr1	npr2	pmi1	pmi2	pmi3
Duple[4,12]	0.072317605	0.981846756	1596.575	21676.492000	22077.266000
Duple[4,11]	0.052838474	0.99045677	2046.521000	38362.020000	38731.645000
Duple[4,16]	0.190472267	0.941045138	1581224.000	7812177.500	8301597.000
Duple[4,13]	0.221238695	0.828878583	3623.775	13576.601	16379.481
Duple[4,9]	0.170879688	0.914837037	3371.237	18048.561	19728.717
Duple[4,15]	0.057862444	0.982970809	2021.1840	34336.0000	34930.8440
Duple[4,6]	0.242322343	0.859005736	1932637.125	6850983.500	7975480.500
Duple[2-3,5]	0.261535858	0.923215089	3992.002	14091.668	15263.689
Duple[4,4]	0.191208522	0.905227895	4453.496	21083.939	23291.305
Duple[4,3]	0.131935755	0.926877323	1609414.75	11306488	12198473
RibufrofenfreeAmino	0.201922861	0.930053209	655346.25	3018513.5	3245527.75
Sibuprofeneamino	0.198352023	0.926533983	656148.938	3064976.5	3308002.25
Rflurbiprofenamino	0.210356292	0.922913753	799741.438	3508772.5	3801842.25
Sflurbiprofenamino	0.202324239	0.924860144	789009.5	3606703	3899728
Duple[4,7]	0.359543168	0.905607534	938849.188	2364747.75	2611228.000
Duple[4,8]	0.130867878	0.979649372	786703.875	5889099.5	6011436
Duple[4,10]	0.076850244	0.954779739	2421.751	30087.592	31512.6
Duple[4,14]	0.18426677	0.901395335	1414775.75	6920793.5	7677867

Table S2. PMI for duples[1,X]

	npr1	npr2	pmi1	pmi2	pmi3
Duple[1,12]	0.131189	0.974602221	716168.625	5320412	5459060
Duple[1,11]	0.094381886	0.989377395	1102087	11552852	11676891
Duple[1,16]	0.169961858	0.996059803	993192	5820592	5843617
Duple[1,13]	0.291488331	0.847888421	3201.082	9311.386	10981.853
Duple[1,9]	0.225429658	0.903249226	2811.916	11266.756	12473.585
Duple[1,15]	0.145869099	0.9512397	1196168.625	7800439.5	8200288
Duple[1,6]	0.139158421	0.95624094	1314083.125	9029853	9443073
bortezomibester	0.261535858	0.923215089	3992.002	14091.668	15263.689

Duple[1,4]	0.159672232	0.939587112	1264298.625	7439732.5	7918087
Duple[1,3]	0.138270068	0.929317797	1606510	10797408	11618639
Ribufrofenfreeester	0.26769379	0.932272108	729241.25	2539660.25	2724162
Sibuprofeneester	0.226273999	0.908404154	2064.96	8290.03	9125.927
Rflurbiprofenester	0.163302512	0.971236807	632237.312	3760212.5	3871571.25
Sflurbiprofenester	0.166984187	0.991155503	647570.188	3843733.75	3878033
Duple[1,7]	0.343030887	0.815332365	3062.612	7279.364	8928.094
Duple[1,8]	0.188349666	0.976963005	955349.250	4955362.500	5072211.000
Duple[1,10]	0.09257767	0.953688599	2775.393	28590.703	29979.076
Duple[1,14]	0.383331459	0.842973262	337341.531	741838.125	880025.688

Table S3. PMI for commercial drugs

	npr1	npr2	pmi1	pmi2	pmi3
Norfloxacinefree	0.218224167	0.846860156	288414.688000	1119247.750000	1321644.125000
HidroxicloroquineLibre	0.181298597	0.992009861	1059.319000	5796.266000	5842.952000
DexametasoneLibre	0.211878055	0.925677164	1159.837000	5067.229000	5474.078000
furosemidefree	0.394175725	0.832041701	1348.066000	2845.551000	3419.962000
Dapsonefree	0.29399611	0.878031467	677.173000	2022.405000	2303.340000
Alprostadilfree	0.320577985	0.717472868	2099.010000	4697.711000	6547.580000
Methotrexatefree	0.264687255	0.826295562	2831.007000	8837.783000	10695.668000
bortezomibfree	0.424072457	0.792847887	2561.566000	4789.116000	6040.397000
sulfasalacinafree	0.088101191	0.955430922	907.890000	9845.794000	10305.082000
FolicAcidFree	0.099554407	0.954589602	1440.457000	13811.998000	14469.043000
Ribufrofenfree	0.145054325	0.971172397	315.181000	2110.210000	2172.848000
SibuprofeneFree	0.175785488	0.957364901	368.590000	2007.419000	2096.817000
Rflurbiprofenfree	0.15875572	0.953045334	455.751000	2735.973000	2870.769000
sflurbiprofenfree	0.140805609	0.941823099	419.944000	2808.929000	2982.438000
tazobactamfree	0.367629268	0.790484959	891.587000	1917.111000	2425.234000
Ciprofloxacinfree	0.223213383	0.802823161	1101.898000	3963.155000	4936.523000
Metoclopramidefree	0.214745468	0.883477672	855.605000	3520.018000	3984.275000
Cillastatinfree	0.247704318	0.961978624	519943.625000	2019240.750000	2099049.500000

[1] PMI analysis was performed using Chembioffice 3D software (v22.0.0.22). After minimization of energy using a Forcefield MMFF94; eps = r, Cutoff [8,10] and Gradient = 0.1 RMS Kcal/mol/A2. The principal moment of inertia and related calculations are performed in units of daltons (AMU) and angstroms. The stochastic conformational search algorithm in the Chem3D software package was used to generate 3D conform-ers for each compound. Sampling and minimization parameters were implemented as follows: Stochastic Search Limit: 7; Refinement Conformation Limit: 300; Stochastic

Search Failure Limit: 100; Stochastic Search Iteration Limit: 1000; Energy Minimization Iteration Limit: 200; Energy Minimization Gradient Test: 0.01. Normalized PMI ratios (I1/I3 and I2/I3) of these conformers were obtained from Chem3D and then plotted on a triangular graph, with the coordinates (0,1), (0.5,0.5) and (1,1) represent-ing a perfect rod, disc and sphere respectively, according to reference 44 of the article.

NMR Spectra

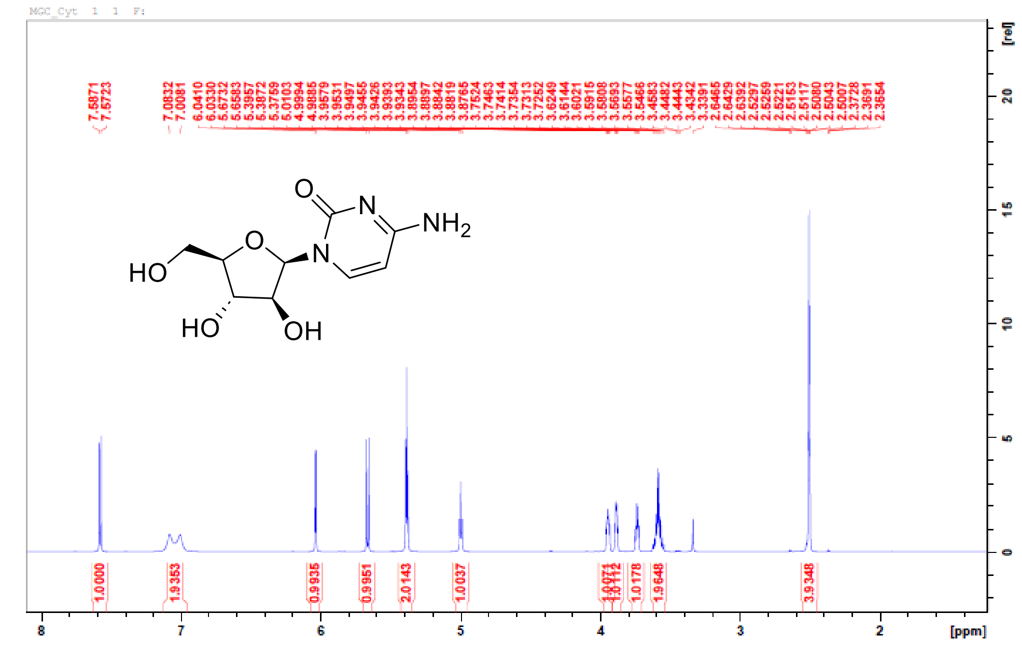

^{1}H-NMR (500 MHz, DMSO-d^6) of Cytarabine

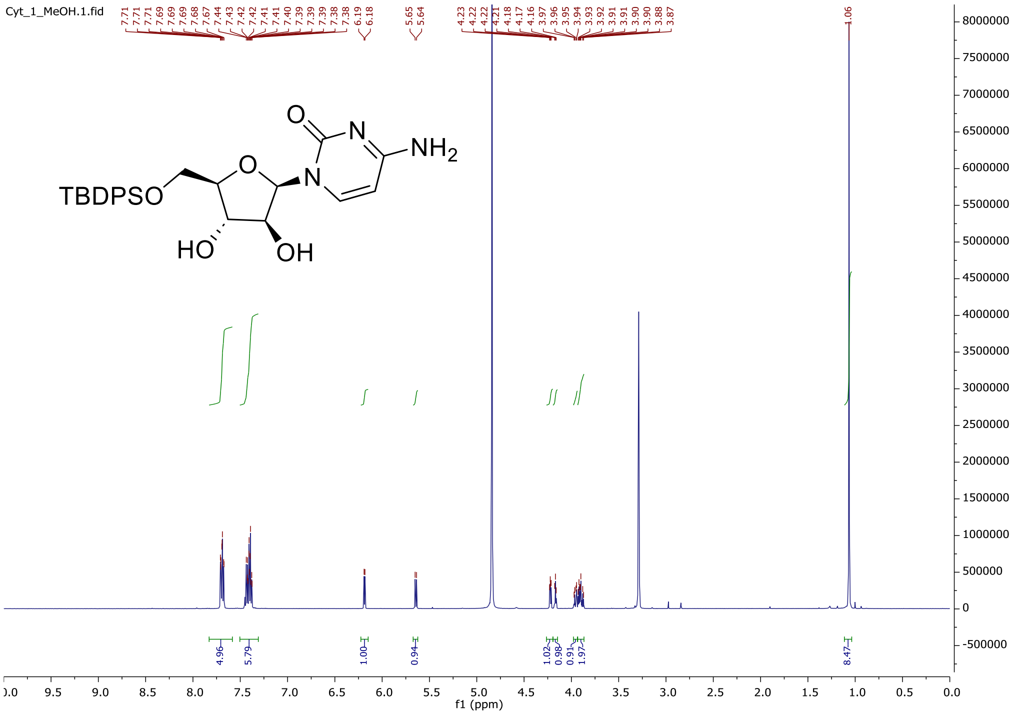

^{1}H-NMR (500 MHz, MeOD-d^4) of intermediate **I** TPSO-Cyt

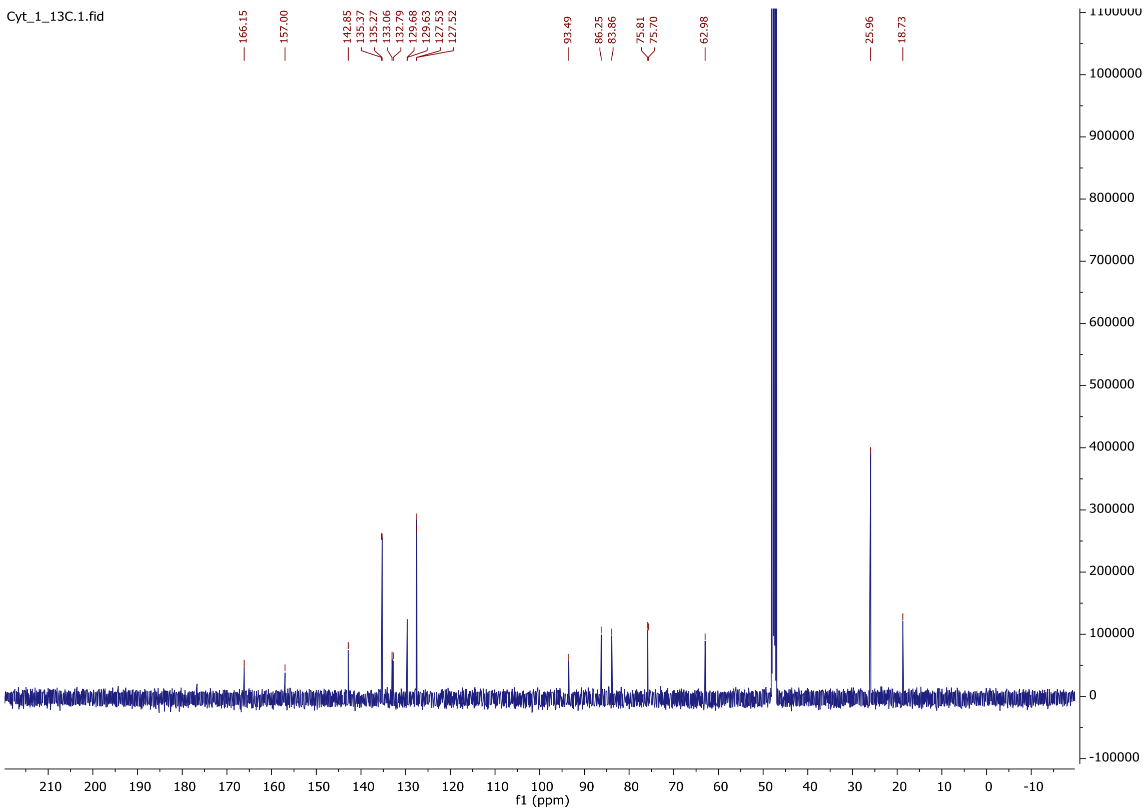

^{13}C-NMR (125 MHz, MeOD-d^4) of intermediate **I** TPSO-Cyt

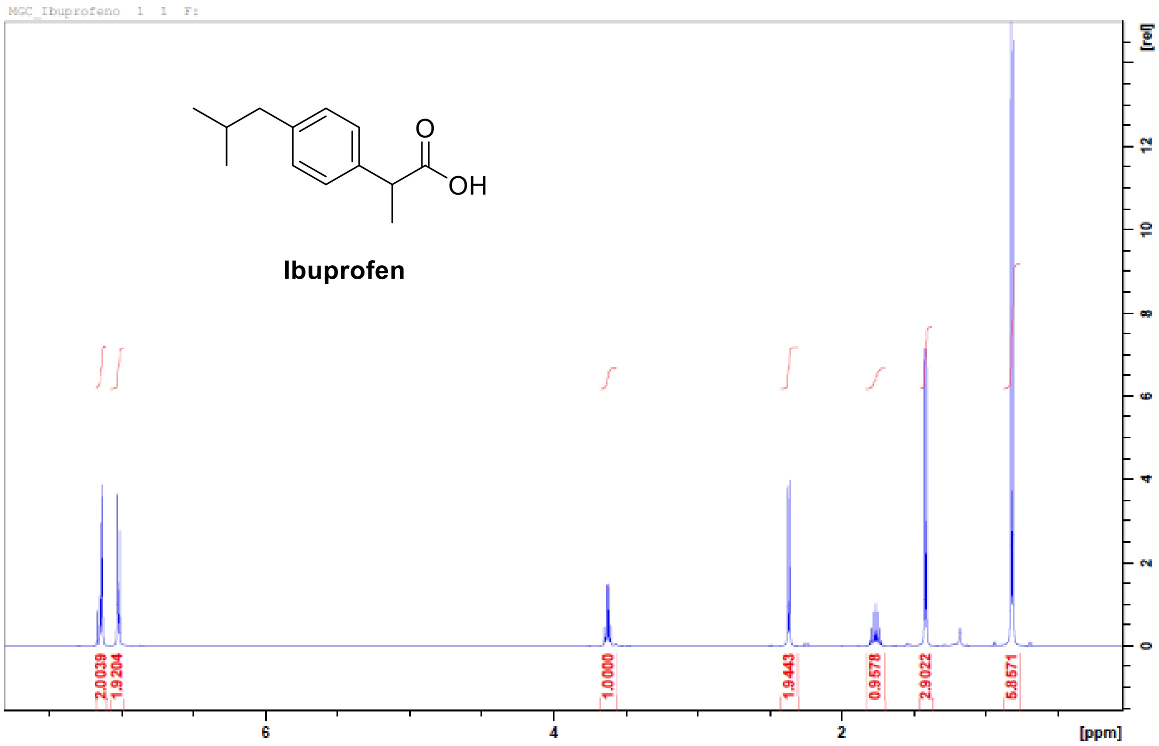

^{1}H-NMR (500 MHz, $CDCl_3$) of Ibuprofen

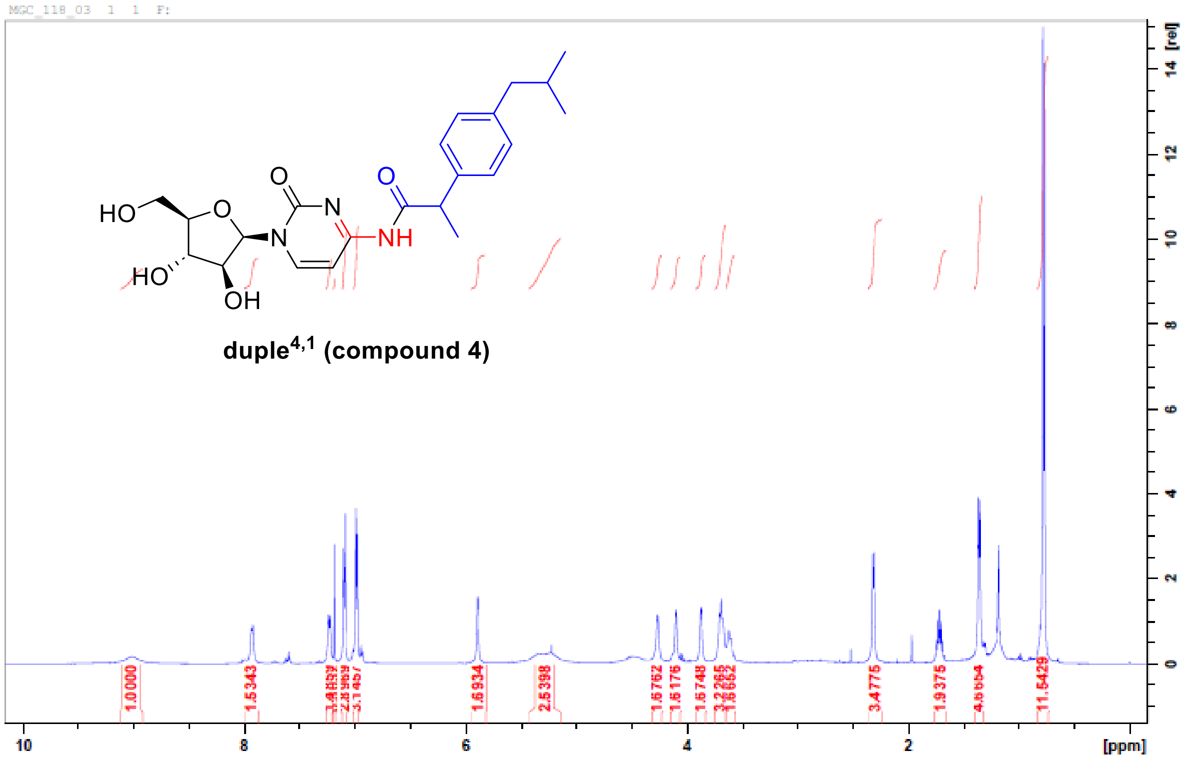

¹H-NMR (500 MHz, CDCl₃) of duple[4,1]

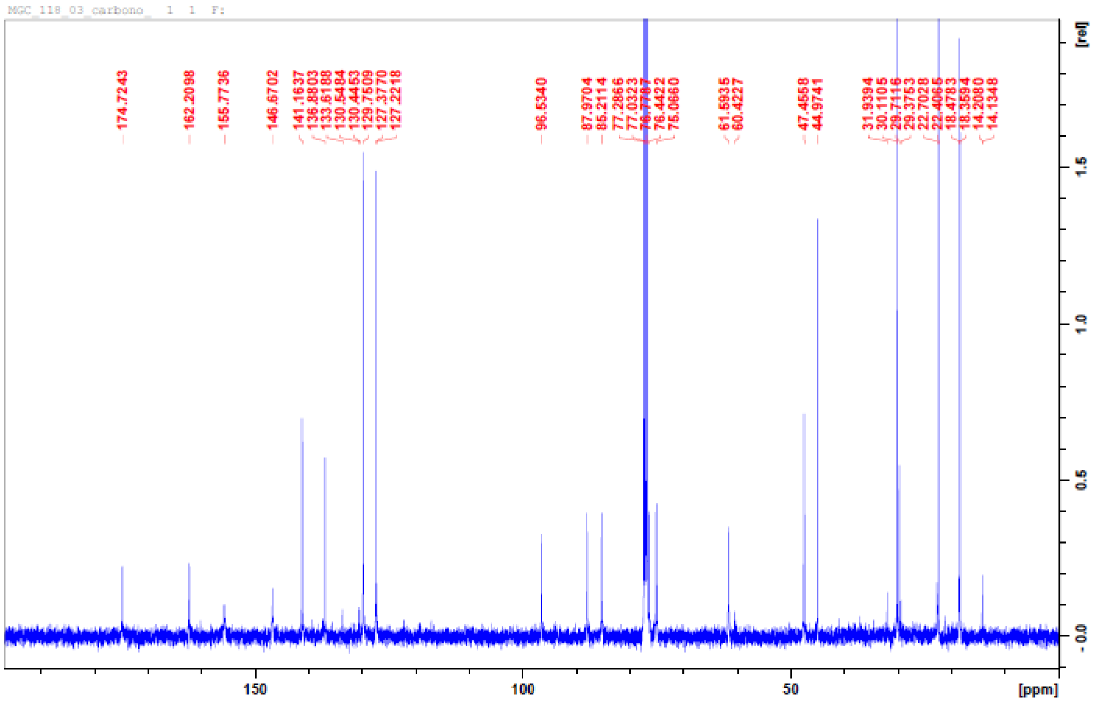

^{13}C-NMR (125 MHz, CDCl₃) of duple[4,1]

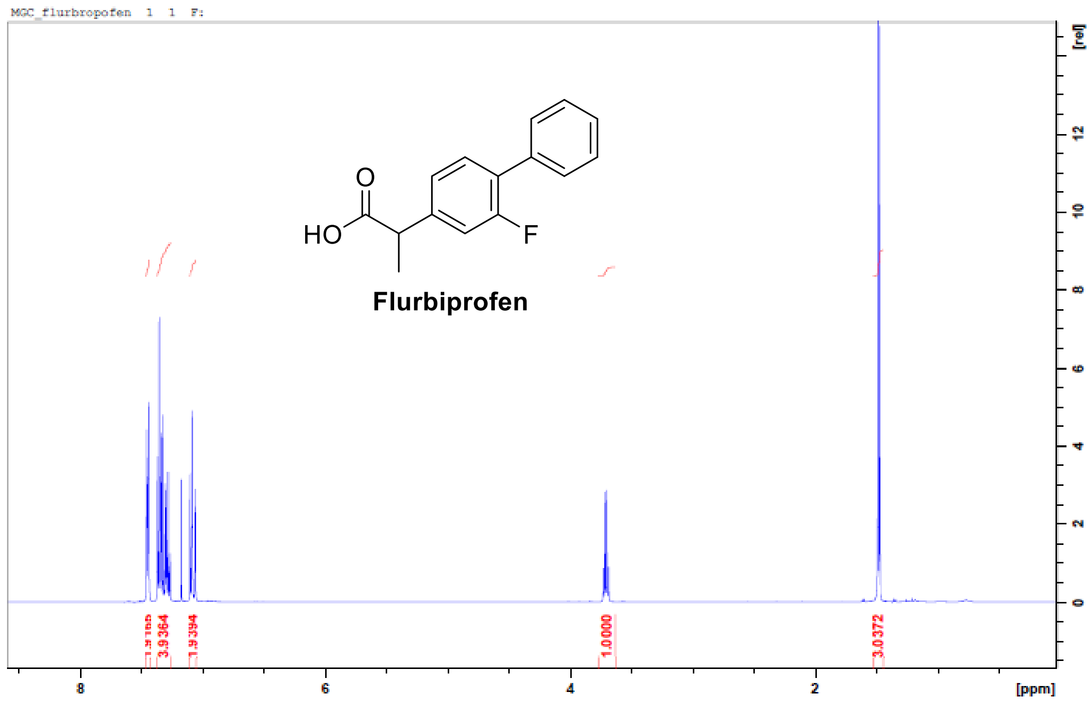

^{1}H-NMR (500 MHz, $CDCl_3$) of Flurbiprofen

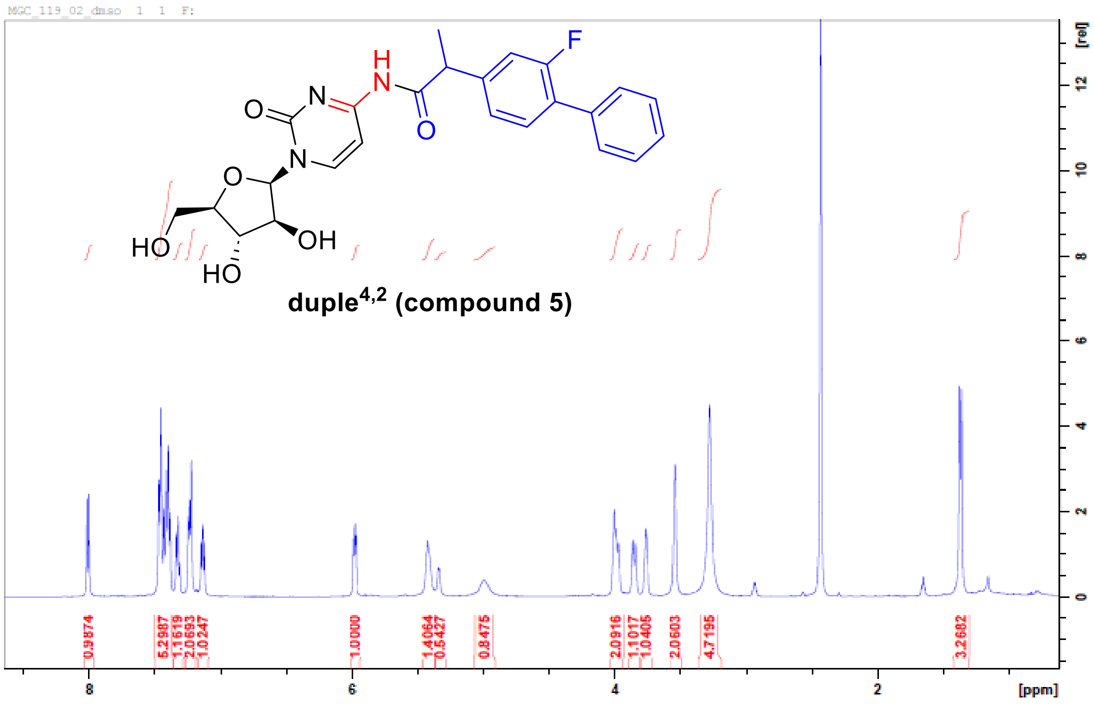

^{1}H-NMR (500 MHz, DMSO-d_6) of duple^{4,2}

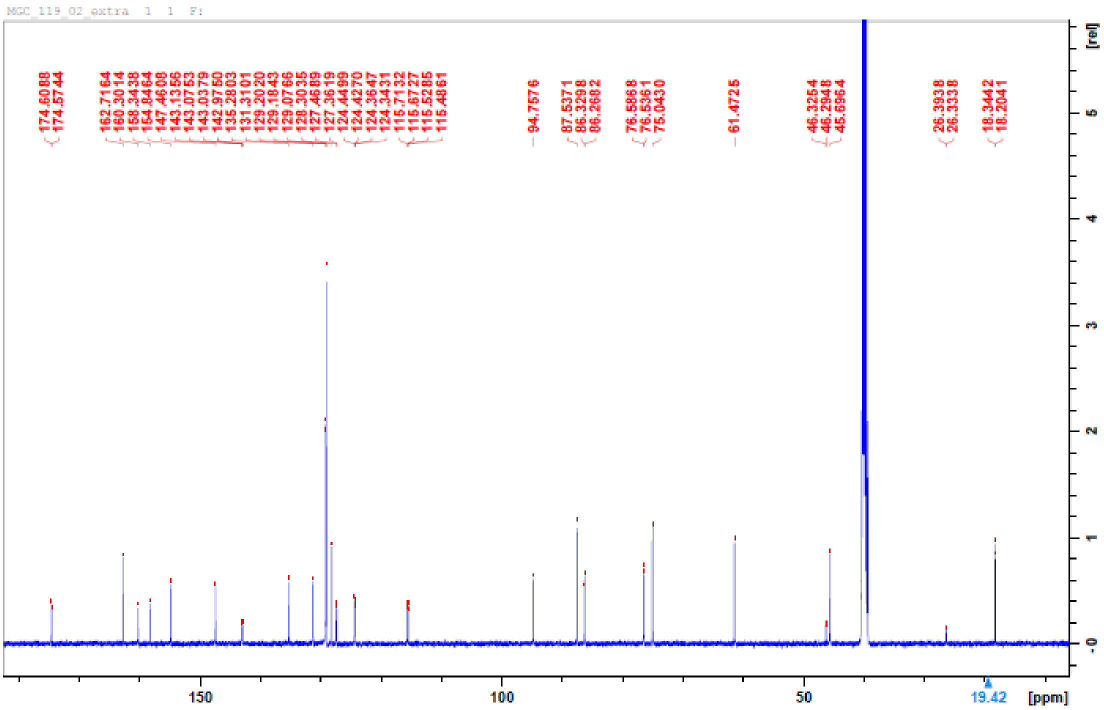

^{13}C-NMR (125 MHz, DMSO-d_6) of duple[4,2]

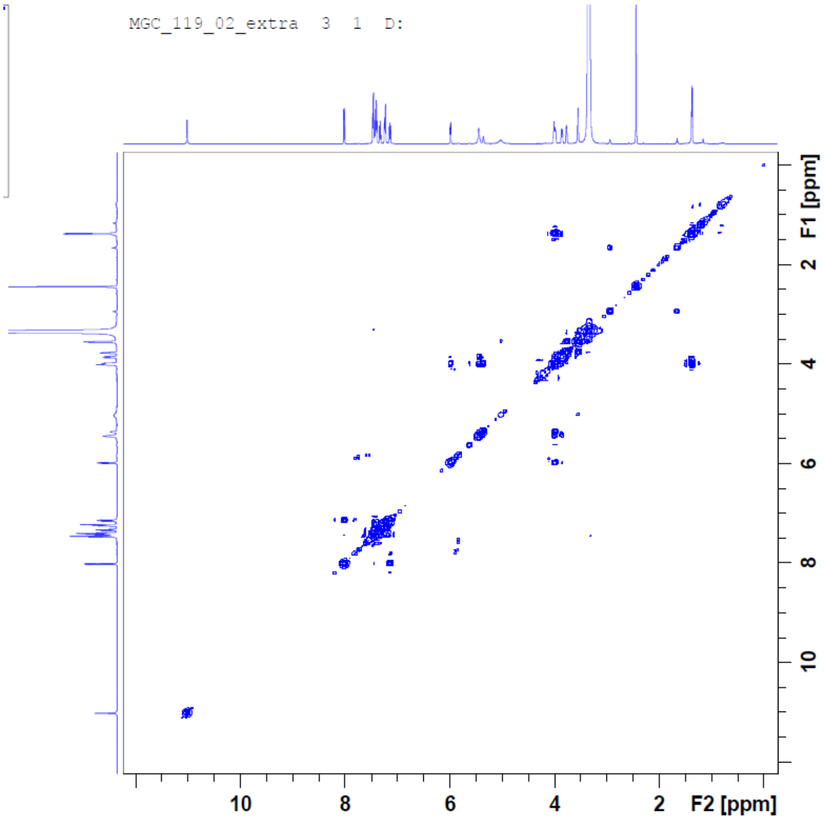

Bidimensional COSY-experiment of duple[4,2]

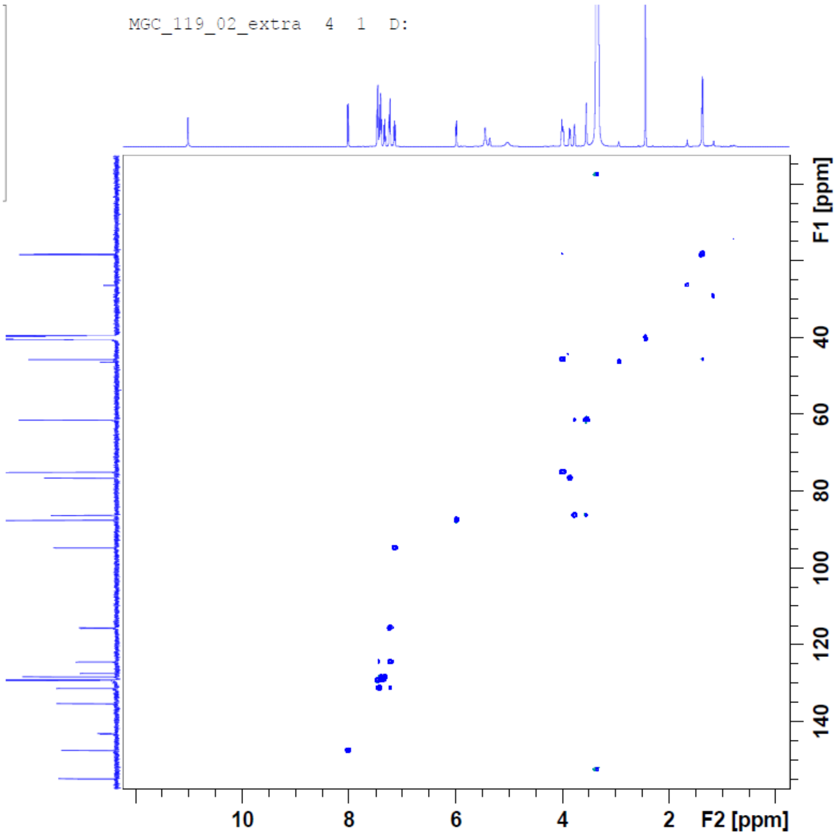

Bidimensional HSQC-experiment of duple[4,2]

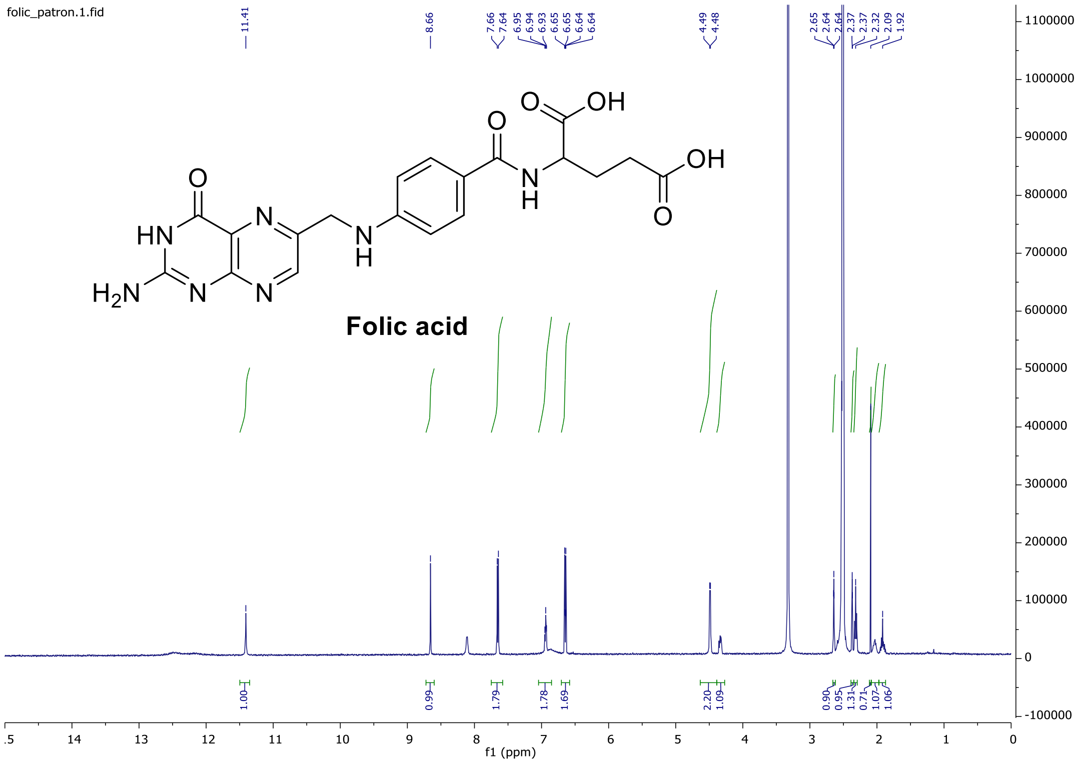

^{1}H-NMR (500 MHz, DMSO-d$_6$) of Folic acid

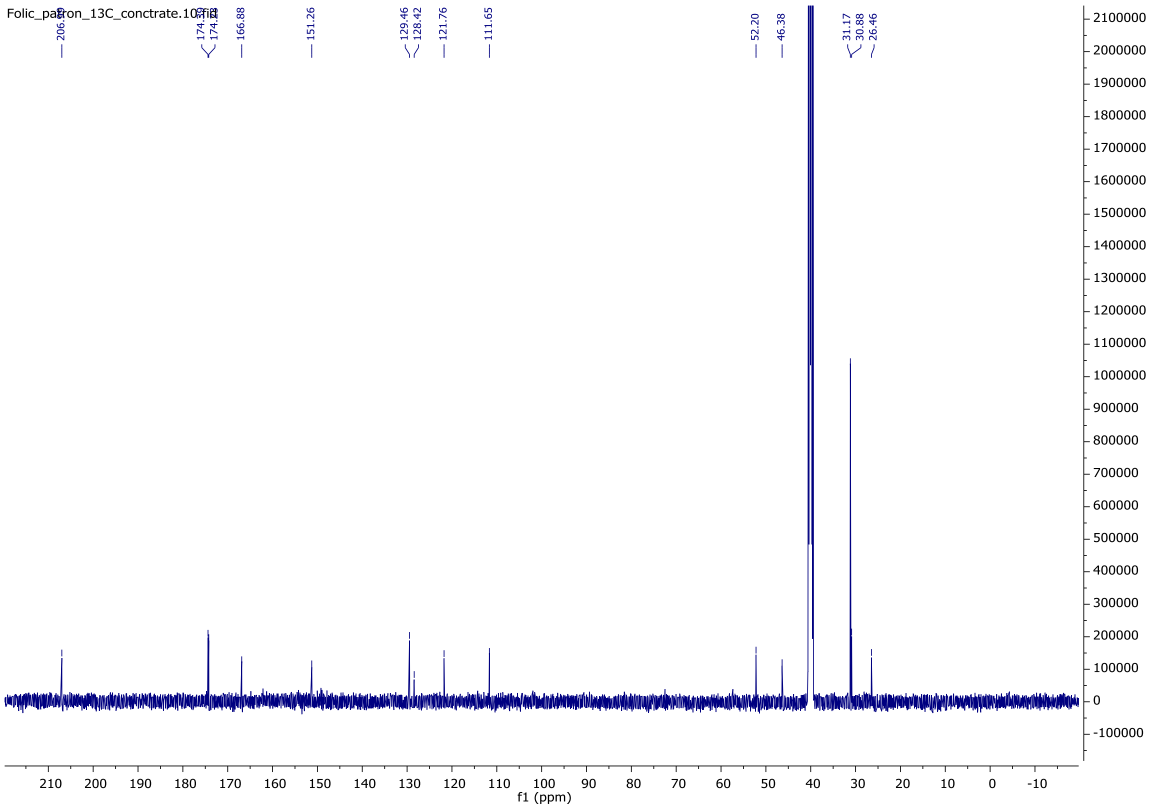

^{13}C-NMR (125 MHz, DMSO-d_6) of Folic acid

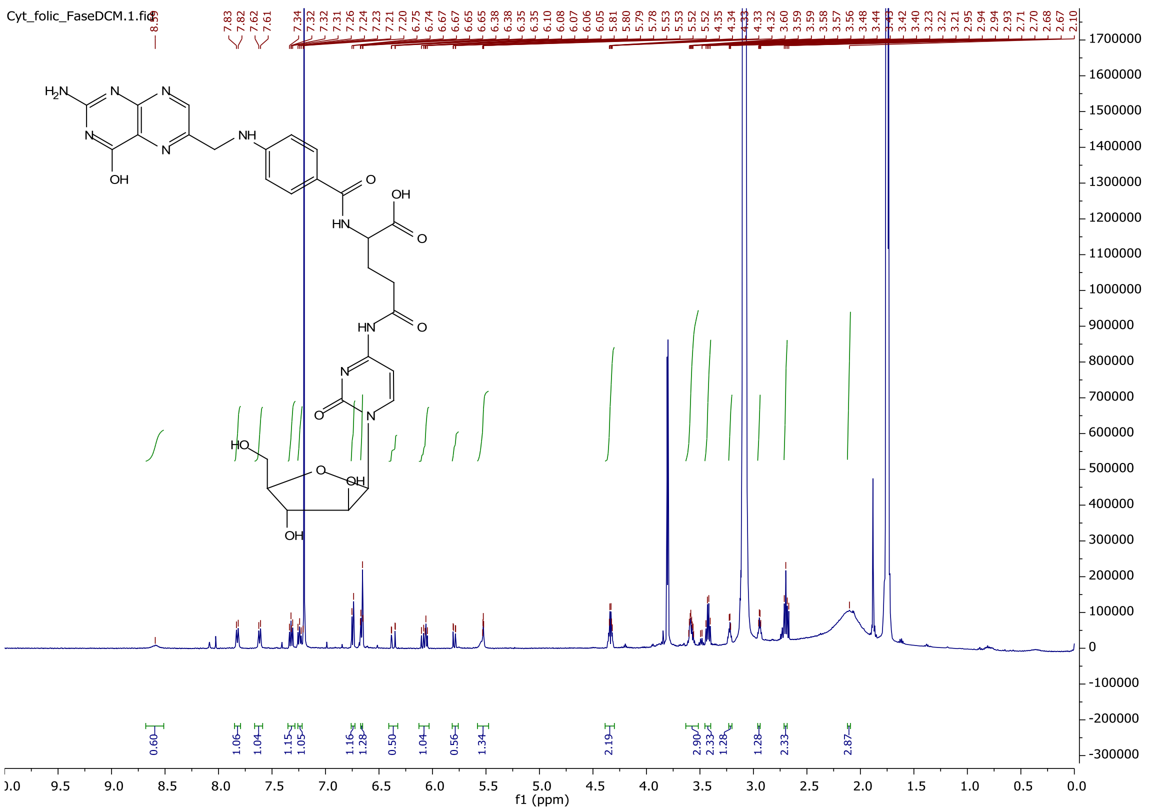

^{1}H-NMR (500 MHz, DMSO-d_6) of duple[4,3]

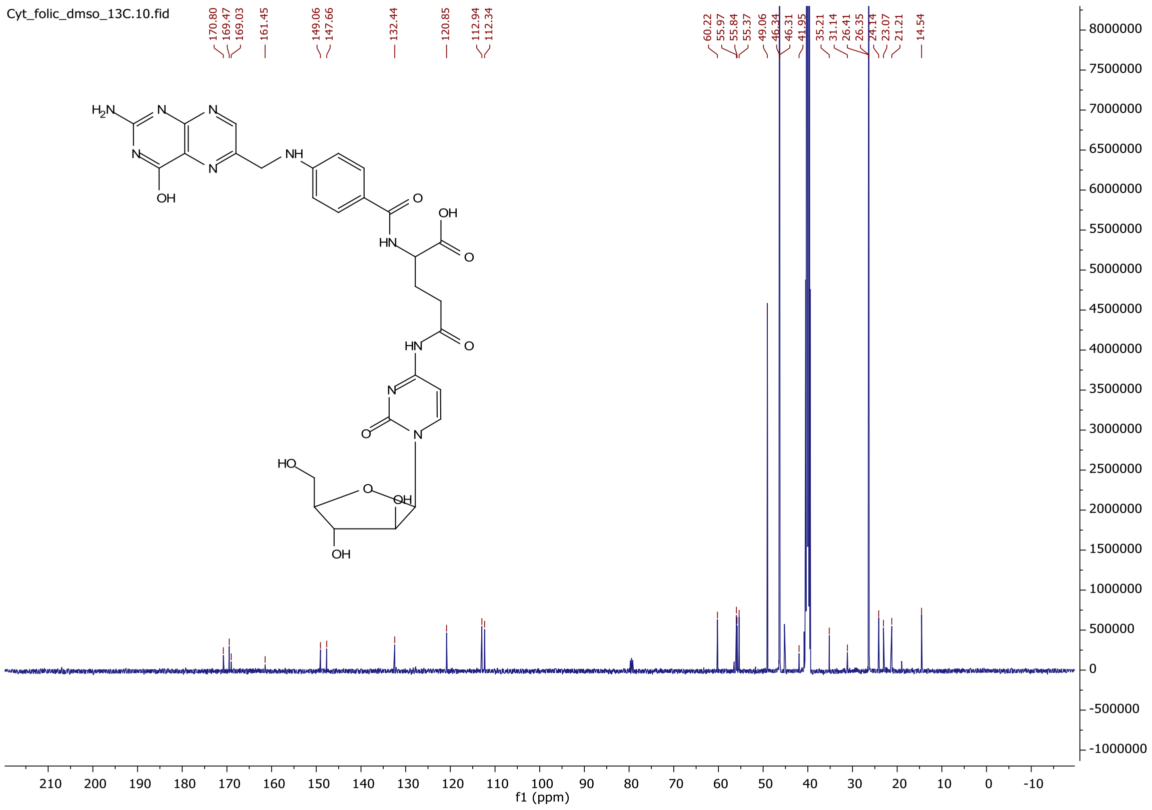

^{13}C-NMR (125 MHz, DMSO-d$_6$) of duple[4,3]

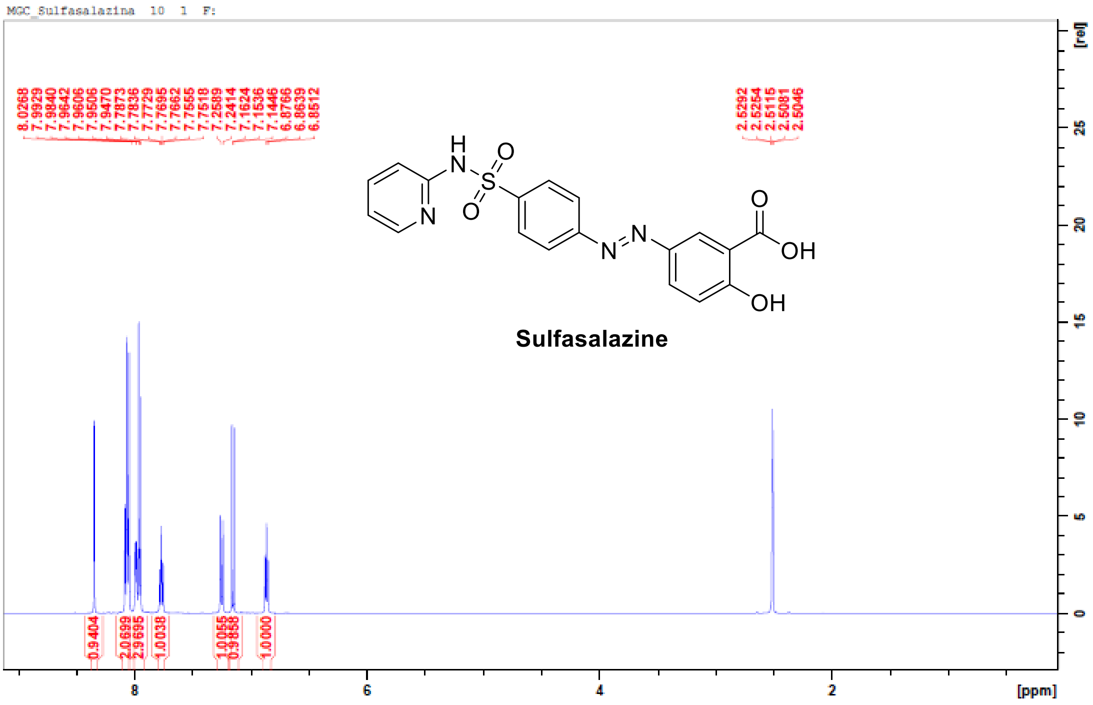

^{1}H-NMR (500 MHz, DMSO-d$_6$) of Sulfasalazine

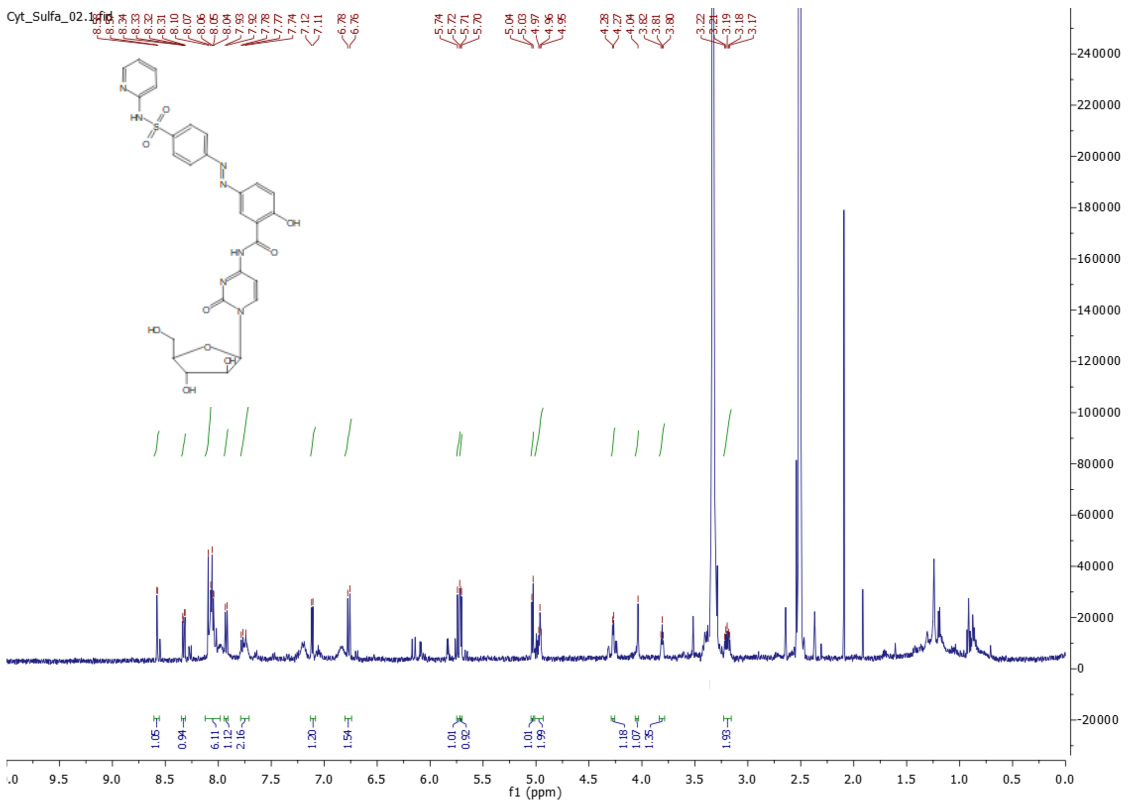

^{1}H-NMR (500 MHz, DMSO-d_6) of duple[4,4]

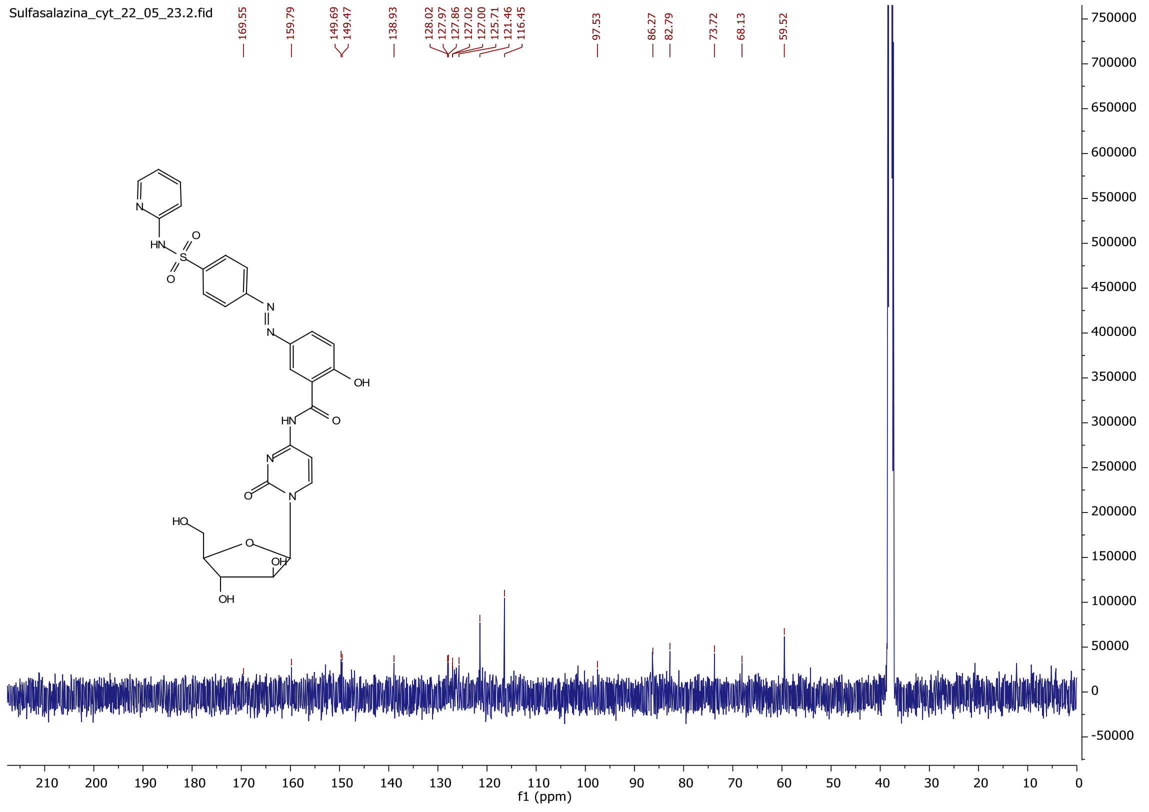

^{13}C-NMR (125 MHz, DMSO-d_6) of duple[4,4]

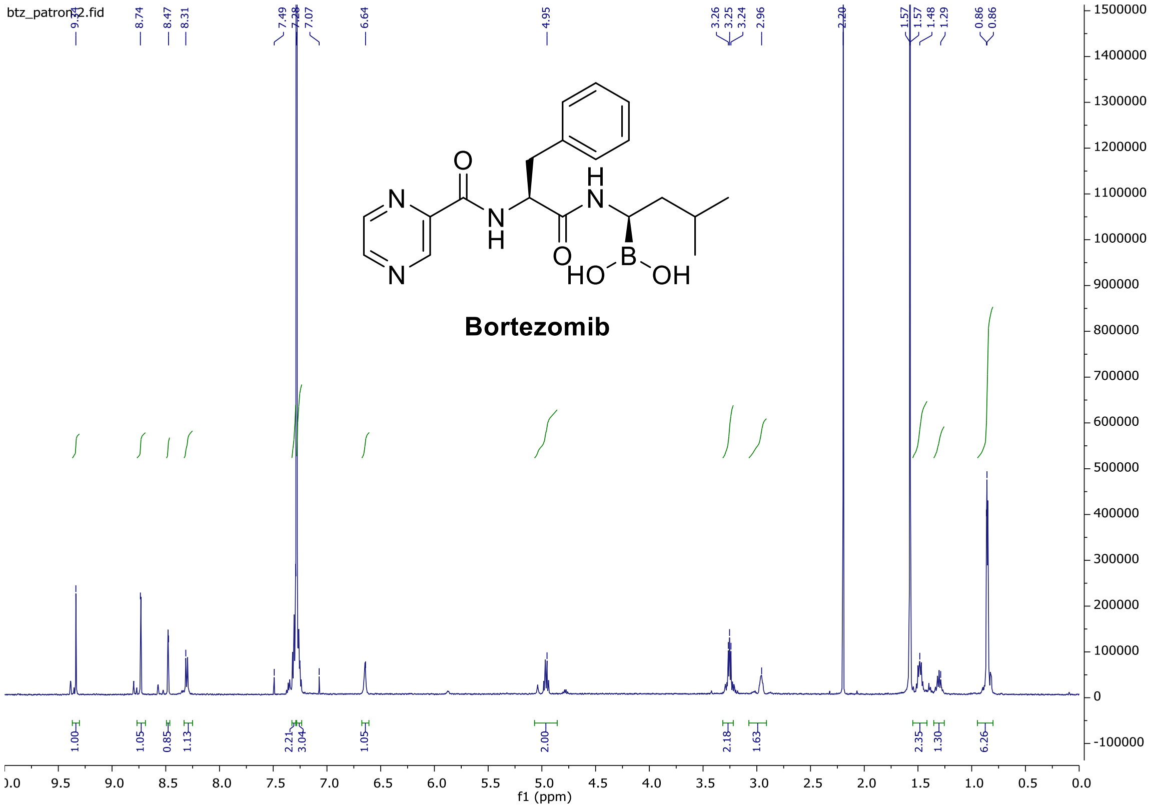

^{1}H-NMR (500 MHz, $CDCl_3$ + drop DMSO-d_6) of Bortezomib

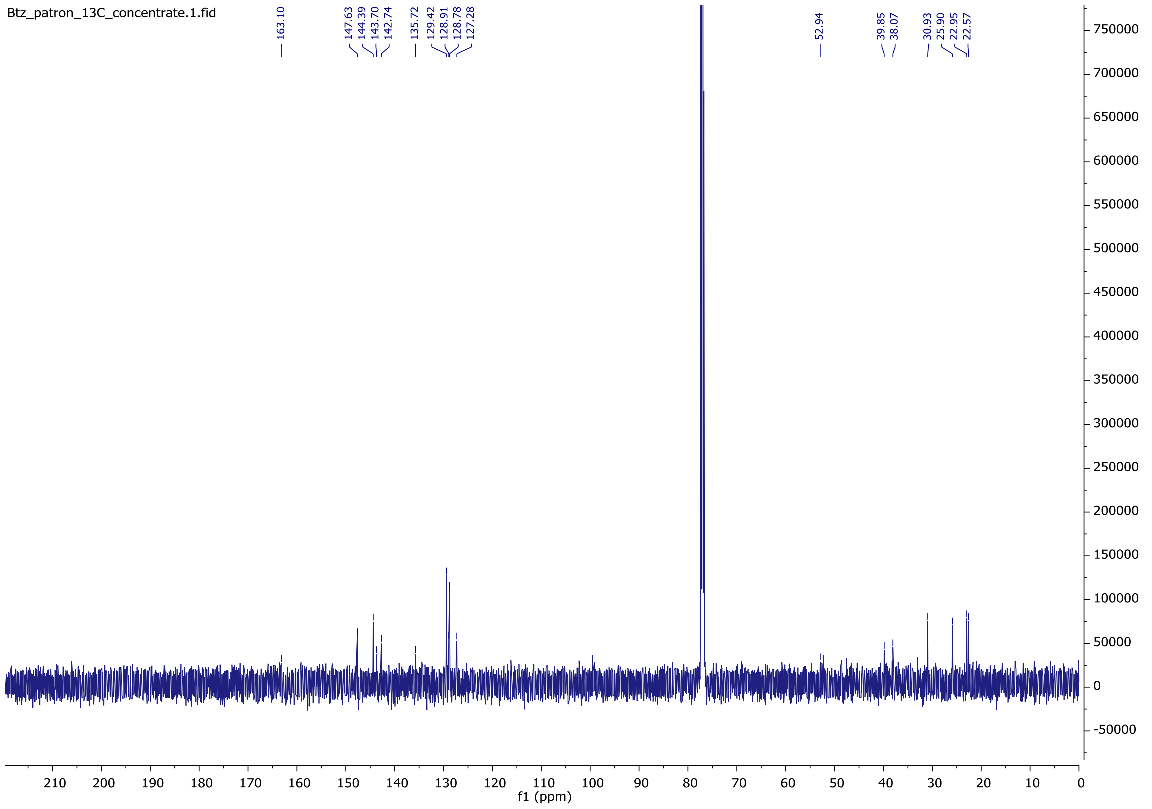

^{13}C-NMR (125 MHz, $CDCl_3$) of Bortezomib

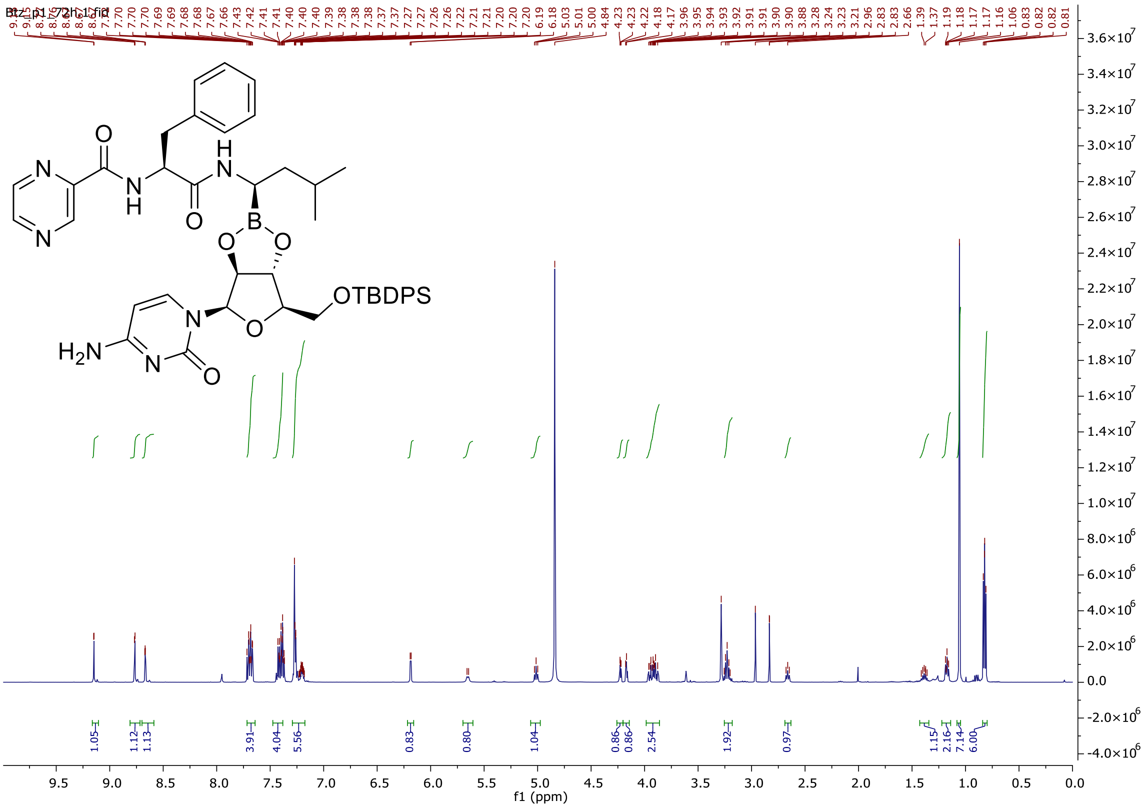

^{1}H-NMR (500 MHz, MeOD-d^4) of intermediate **II** TPSO-duple[2-3,5]

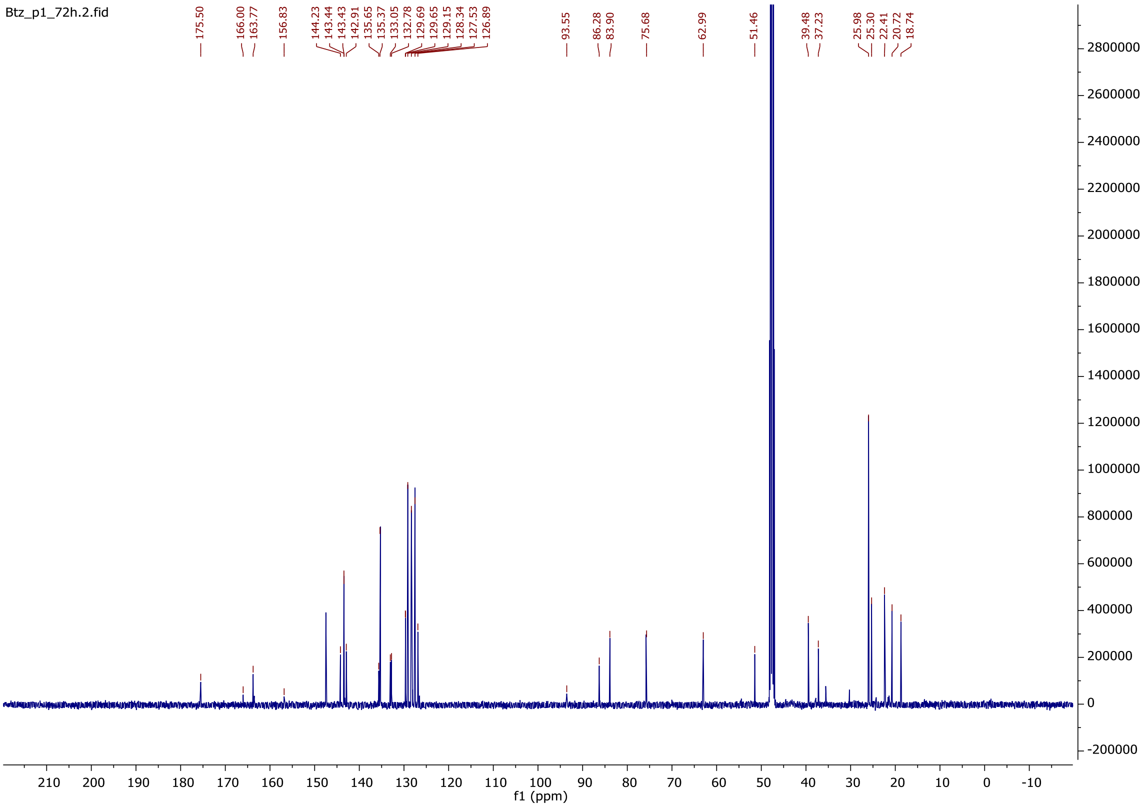

^{13}C-NMR (125 MHz, MeOD-d^4) of intermediate **II** TPSO-duple[2-3,5]

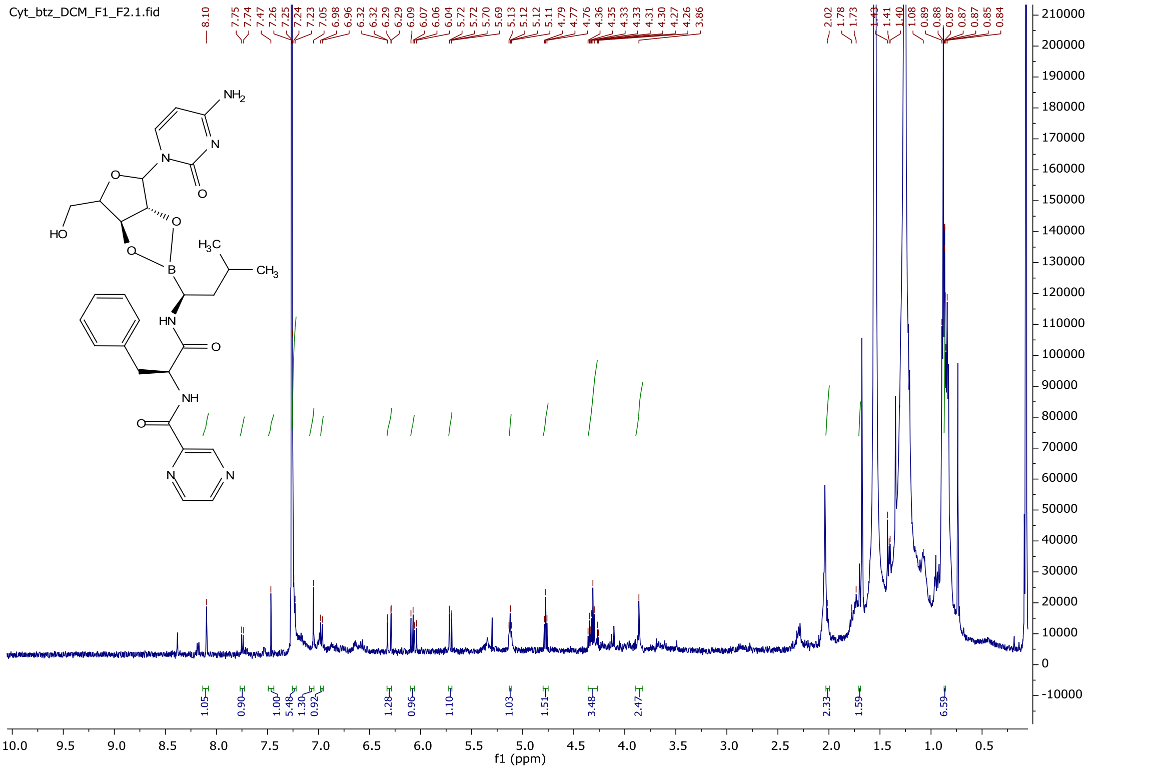

^{1}H-NMR (500 MHz, $CDCl_3$) of duple[2-3,5]

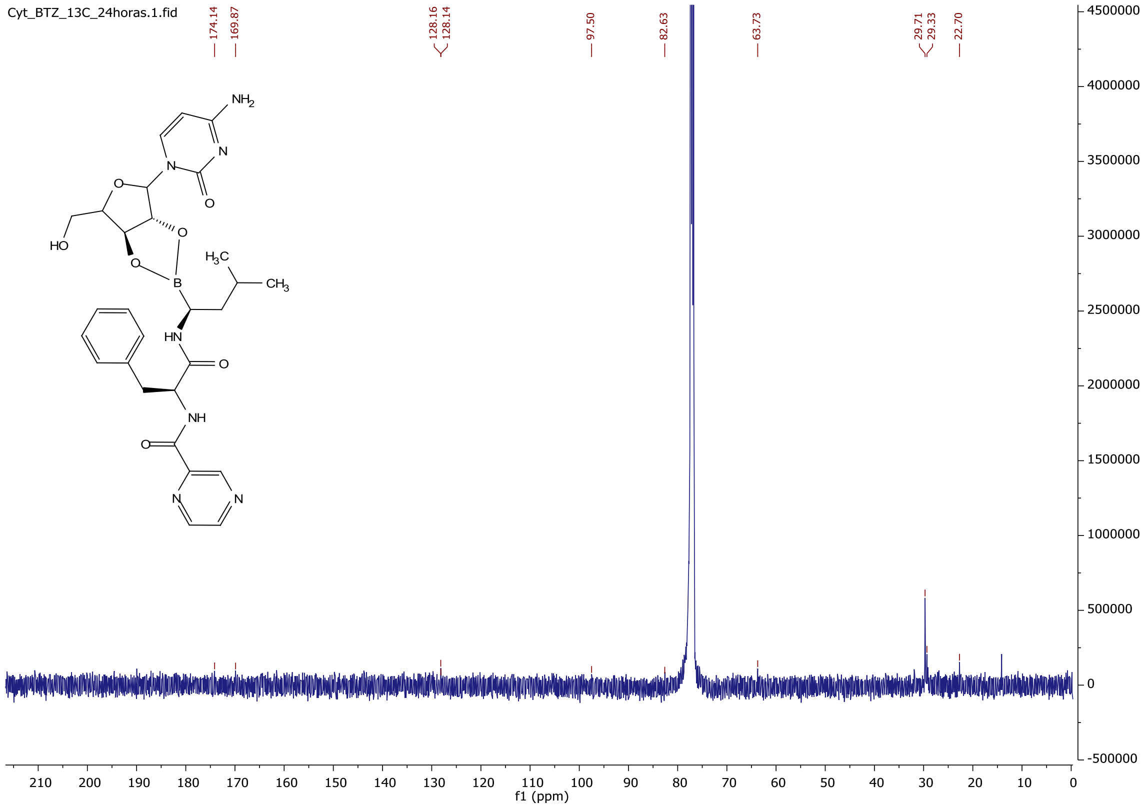

^{13}C-NMR (125 MHz, $CDCl_3$) of duple[2-3,5]

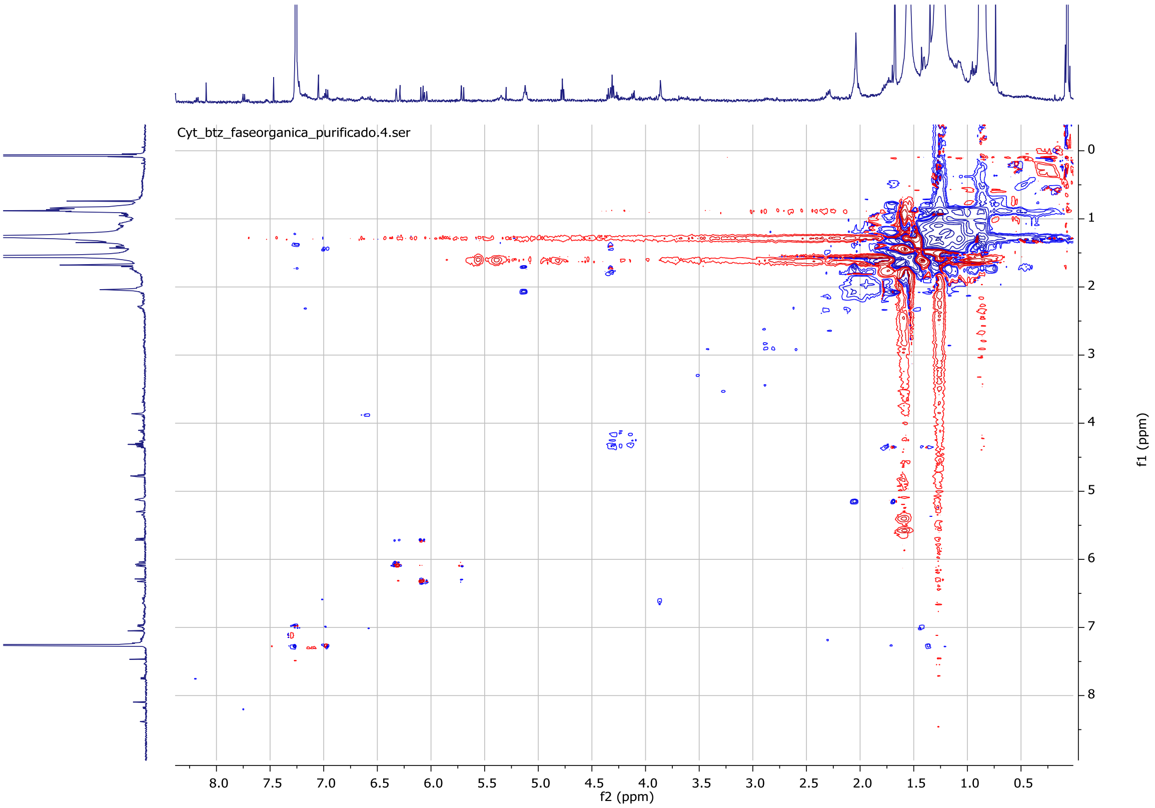

Bidimensional NOESY-experiment of duple[2-3,5]

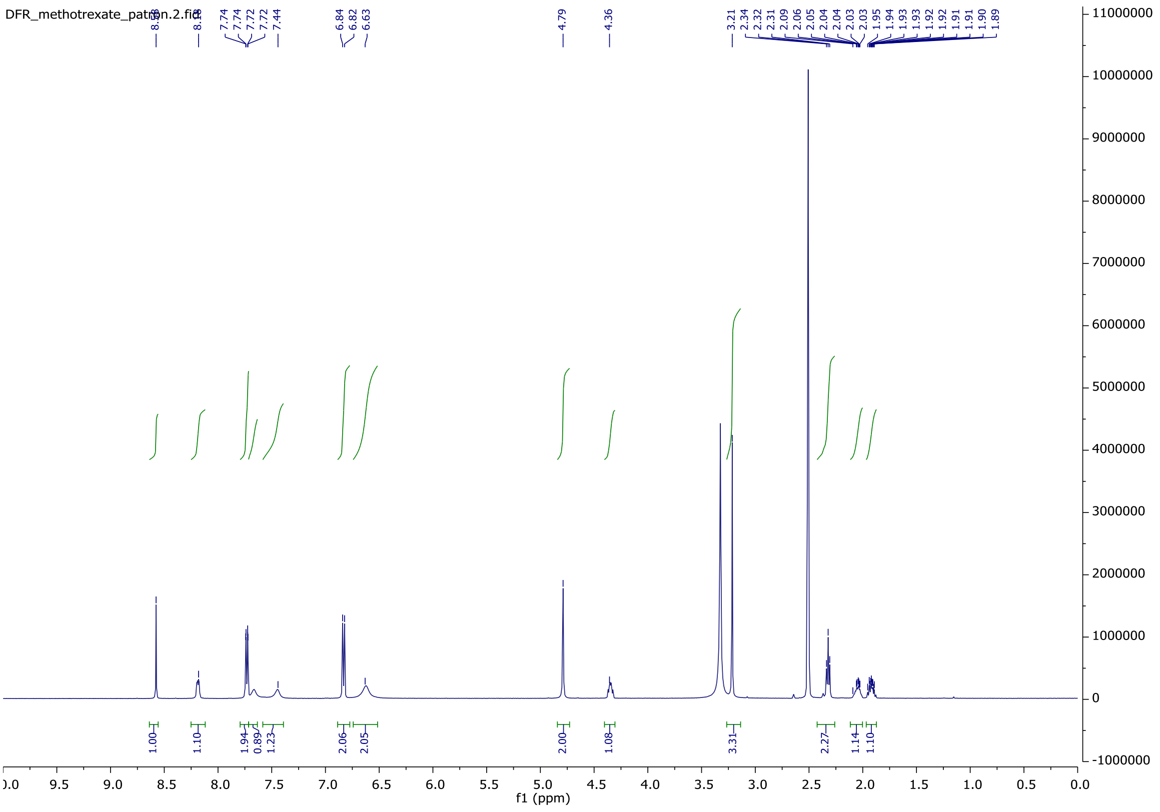

^{1}H-NMR (500 MHz, DMSO-d_6) of Methotrexate

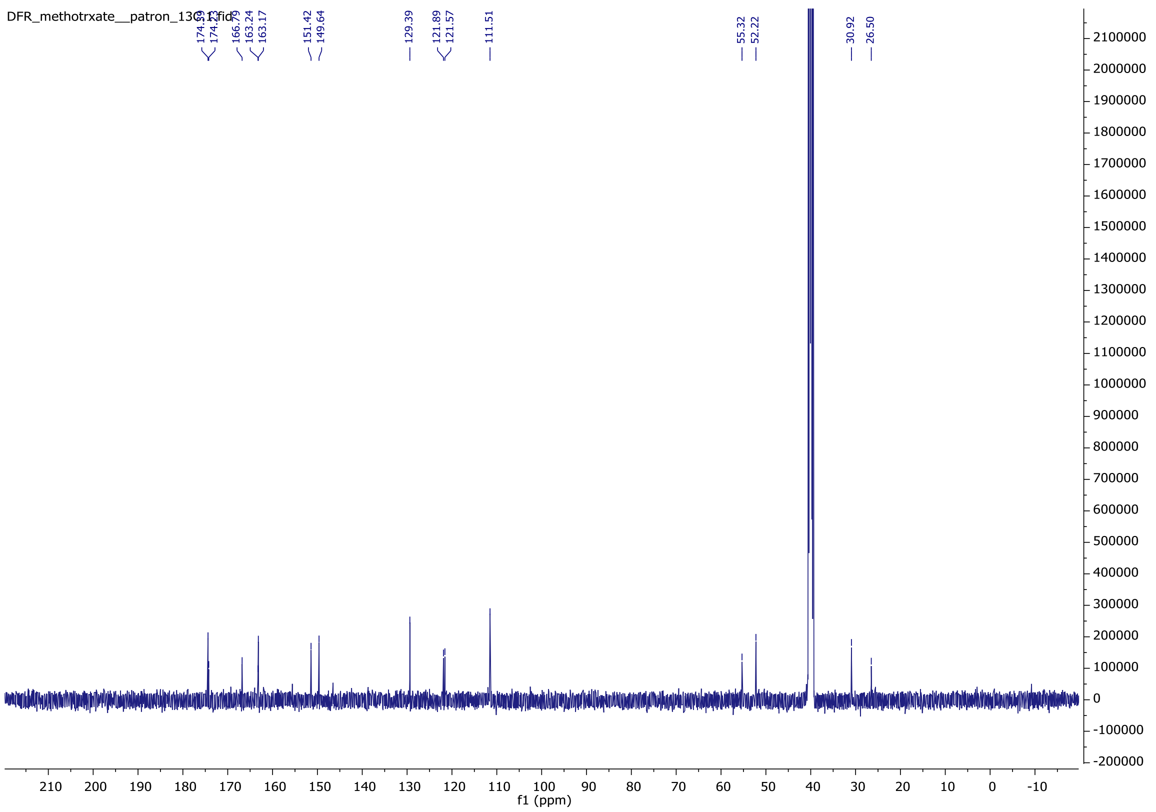

^{13}C-NMR (125 MHz, DMSO-d$_6$) of Methotrexate

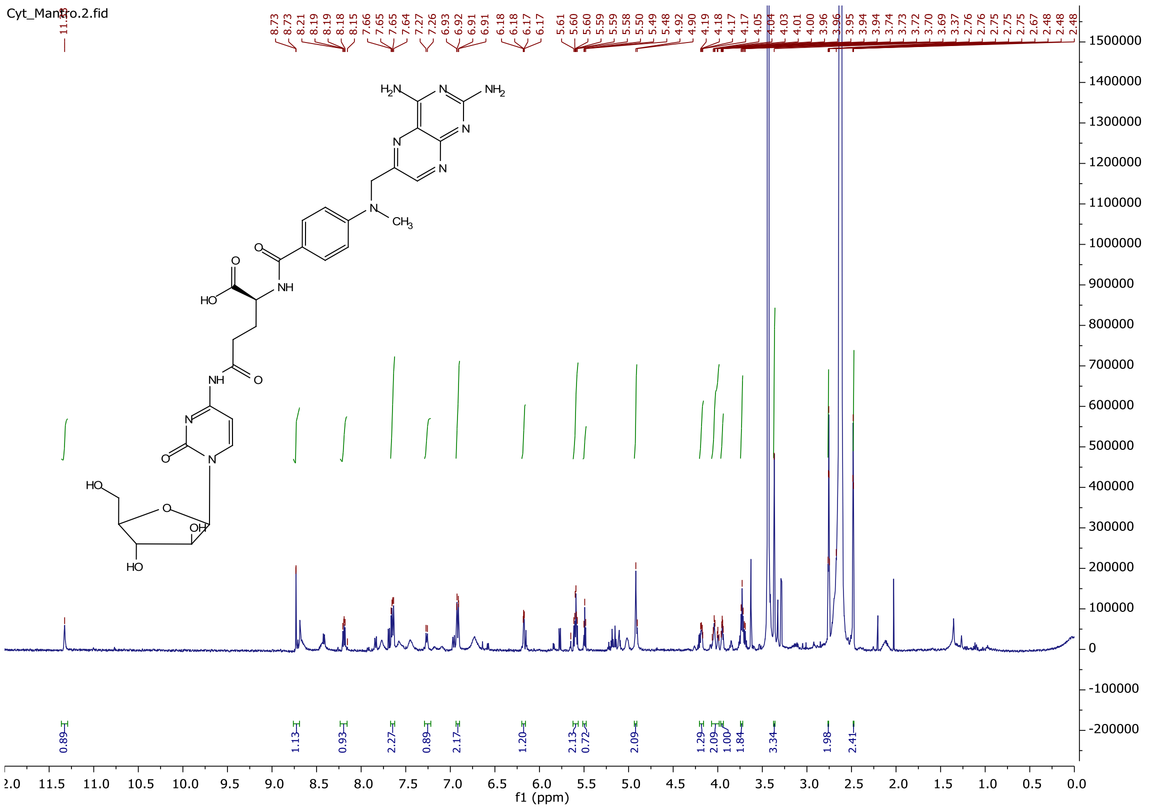

^{1}H-NMR (500 MHz, DMSO-d$_6$) of duple[4,6]

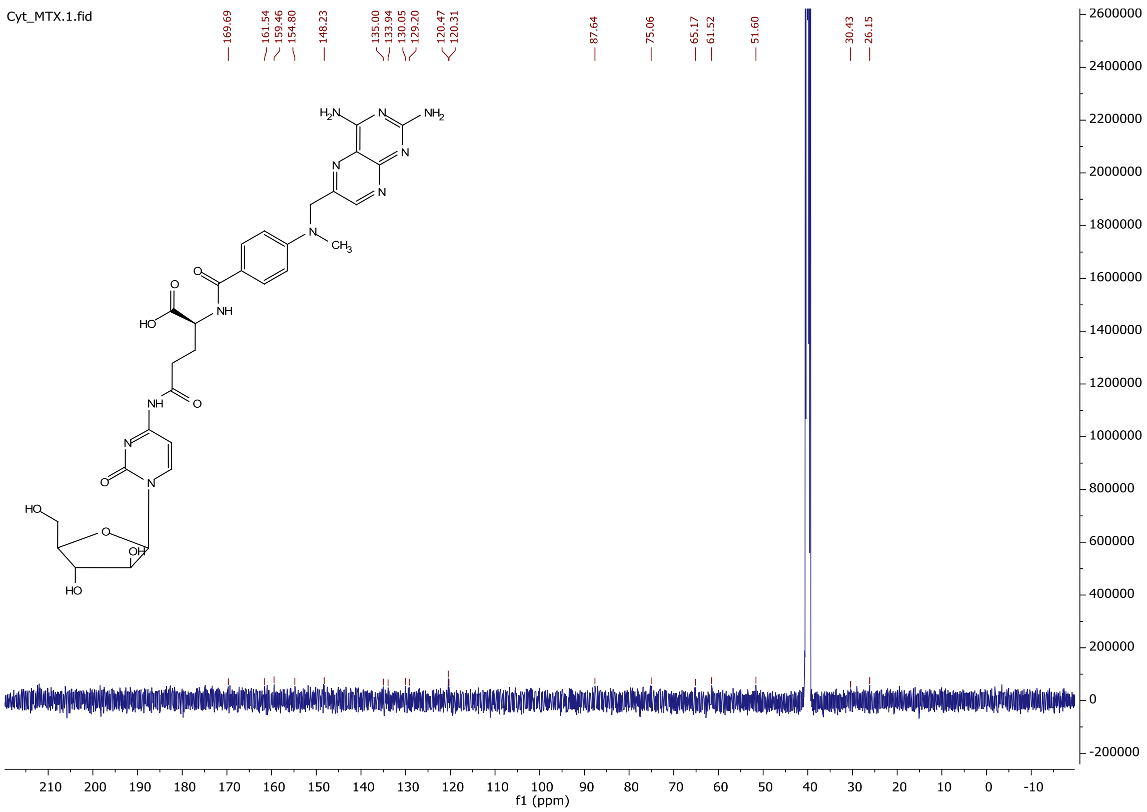

^{13}C-NMR (126 MHz, DMSO-d$_6$) of duple[4,6]

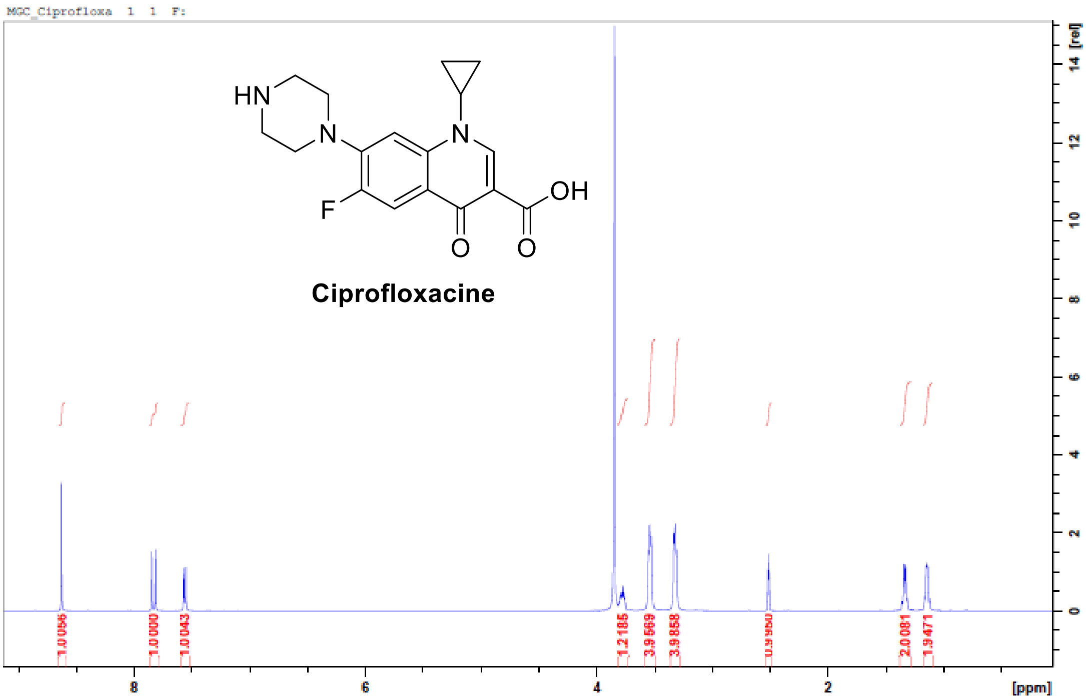

^{1}H-NMR (400 MHz, DMSO-d$_6$ + drops of D_2O) of Ciprofloxacine

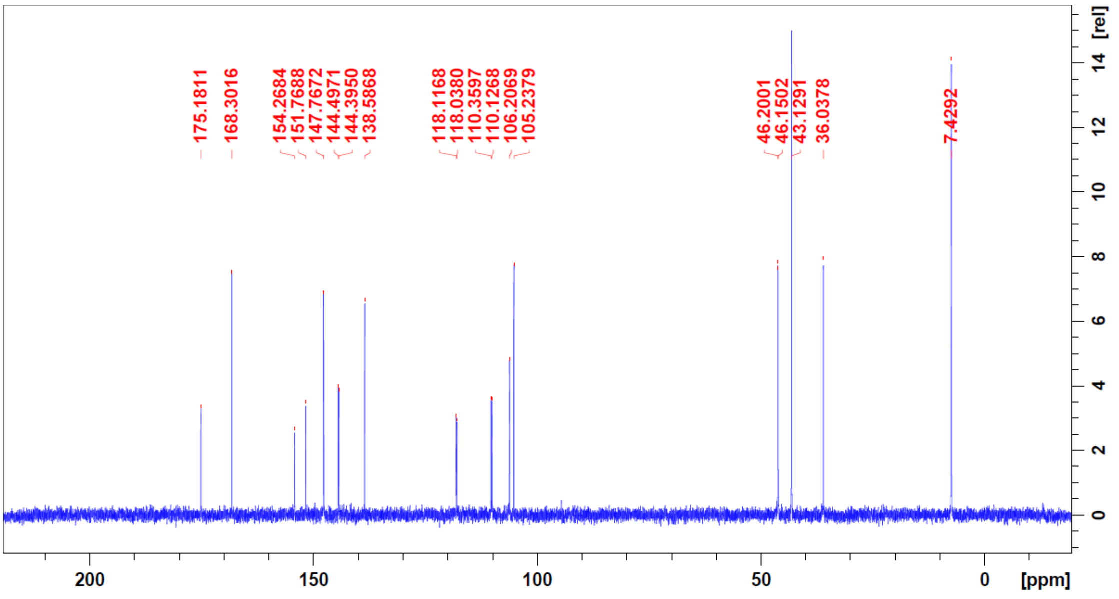

^{13}C-NMR (100 MHz, D_2O) of Ciprofloxacine

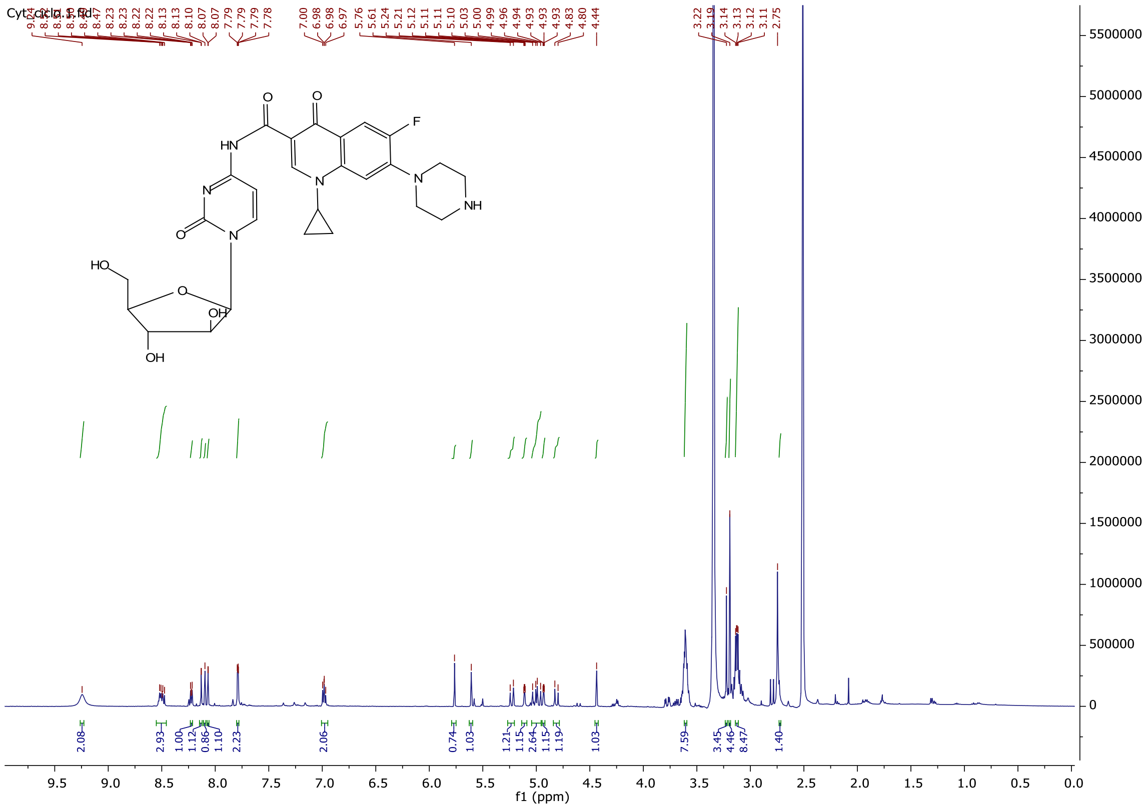

^{1}H-NMR (500 MHz, DMSO-d_6) of duple[4,8]

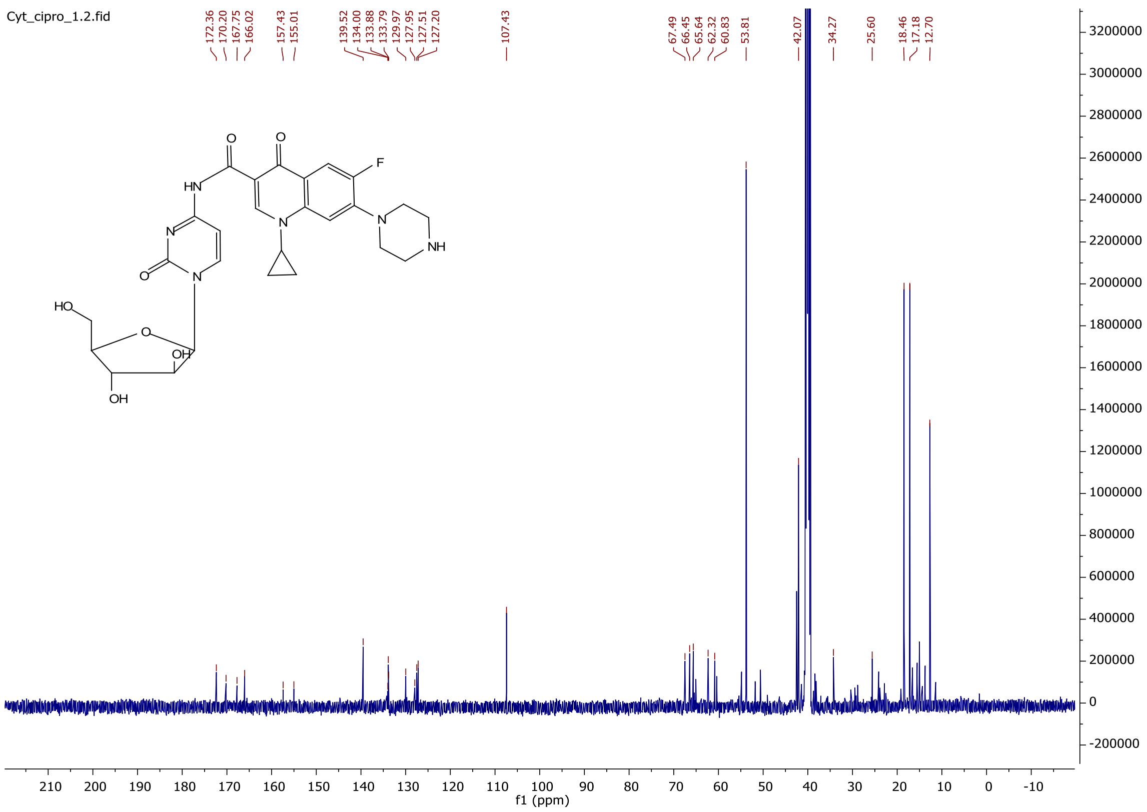

^{13}C-NMR (126 MHz, DMSO-d_6) of duple[4,8]

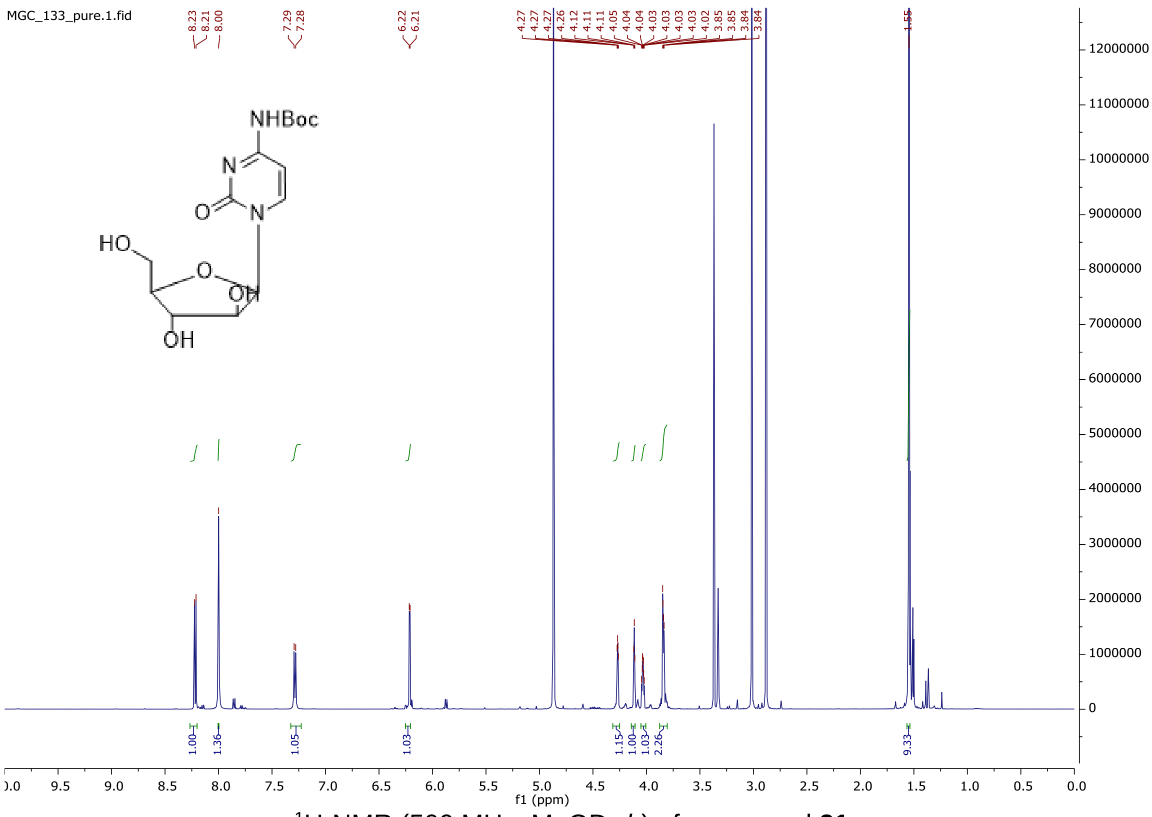

^{1}H-NMR (500 MHz, MeOD-d_4) of compound **21**

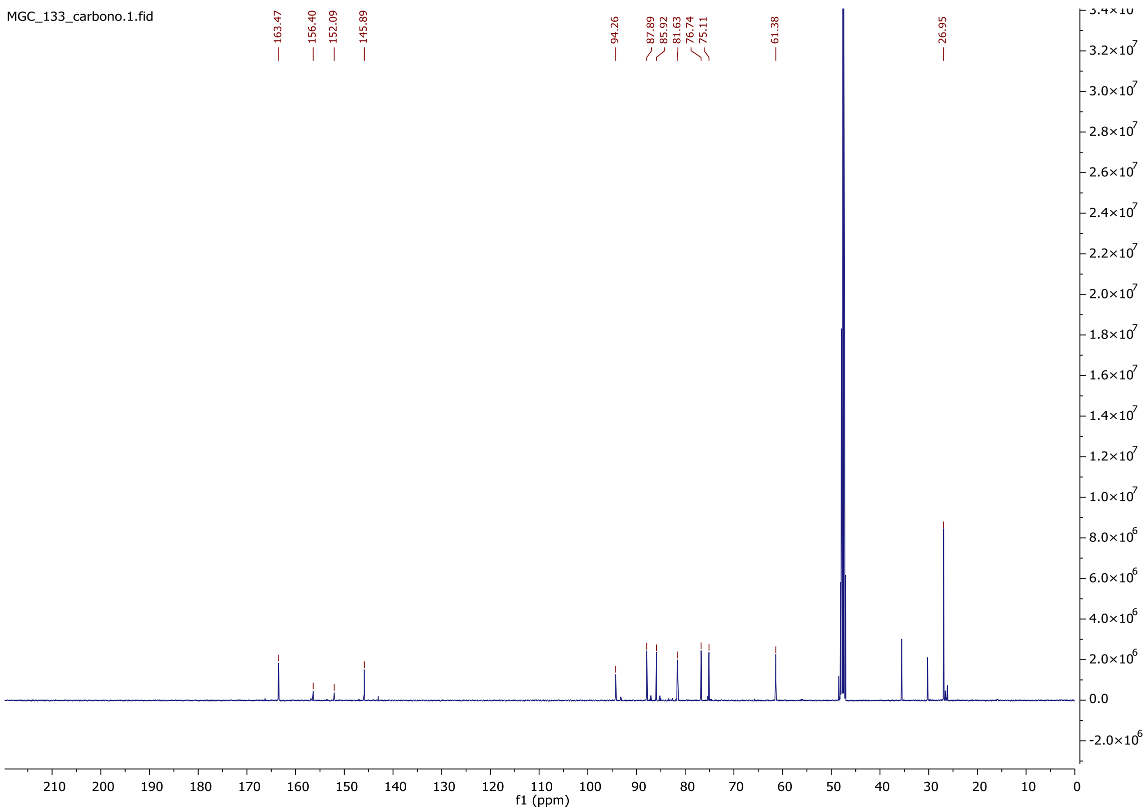

^{13}C-NMR (125 MHz, MeOD-d_4) of compound **21**

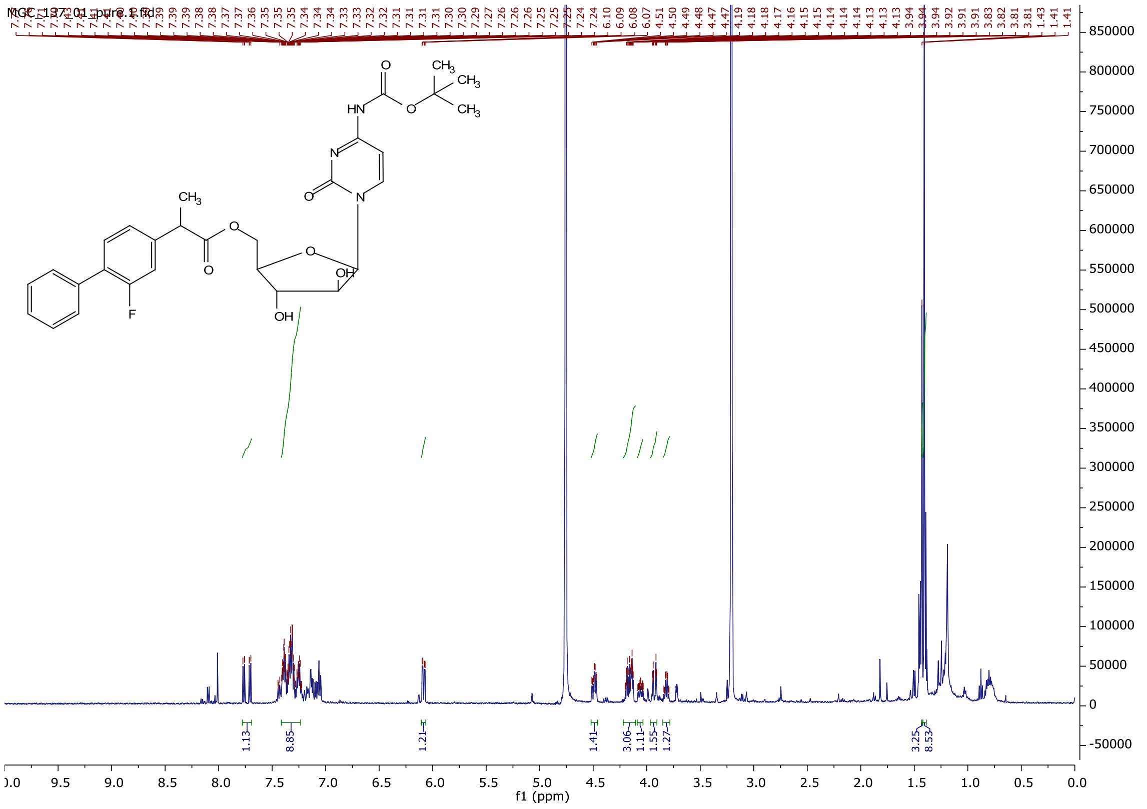

^{1}H-NMR (500 MHz, MeOD-d_4) of Boc-duple[1,2]

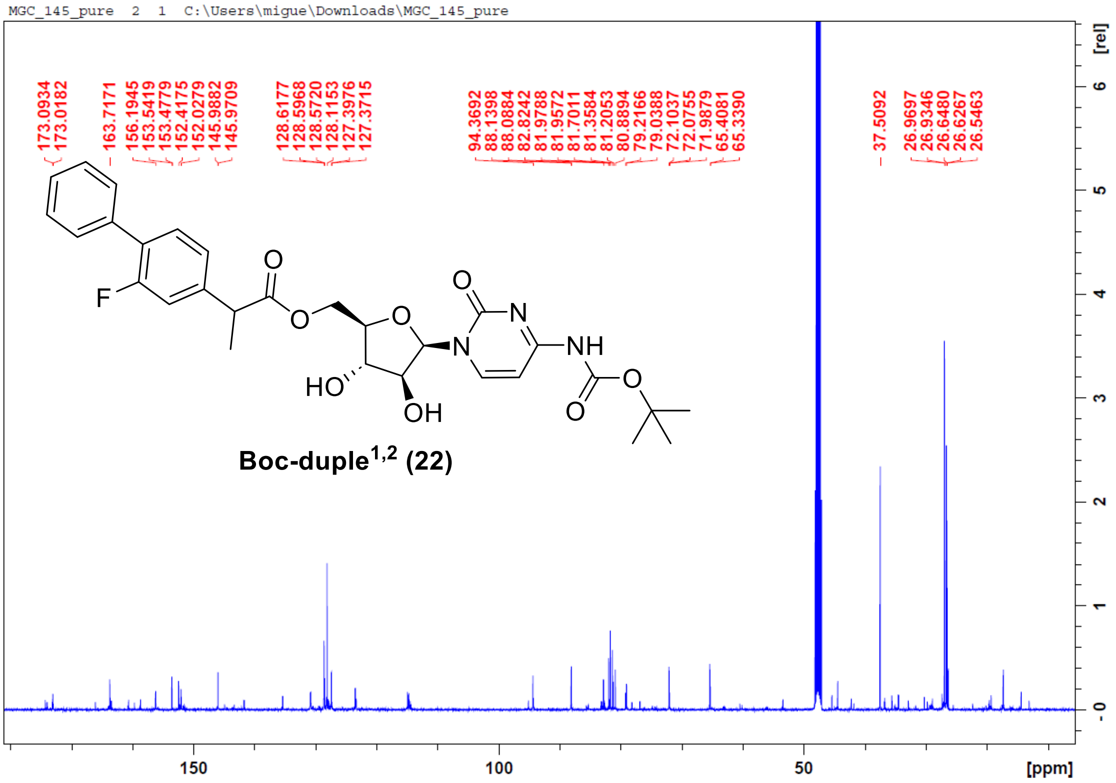

^{13}C-NMR (125 MHz, MeOD-*d*$_4$) of Boc-duple[1,2] (**22**)

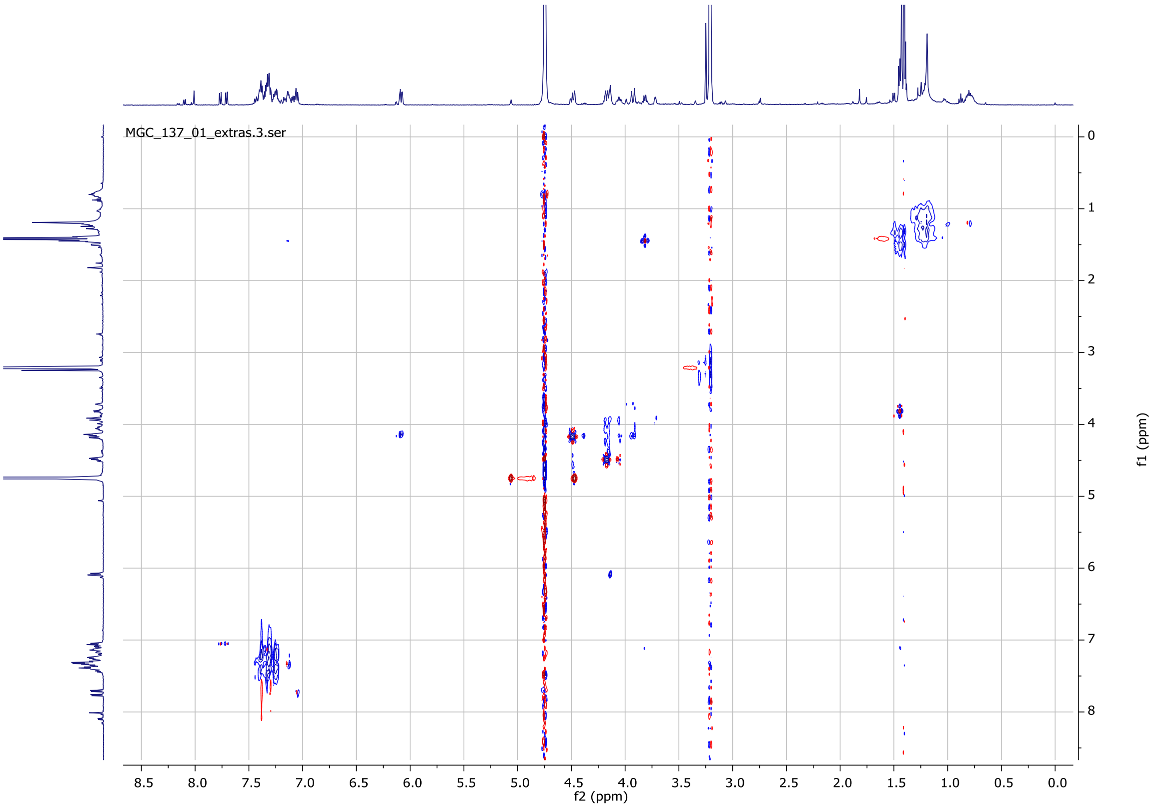

Bidimensional NOESY-experiment of Boc-duple[1,2] (**22**)

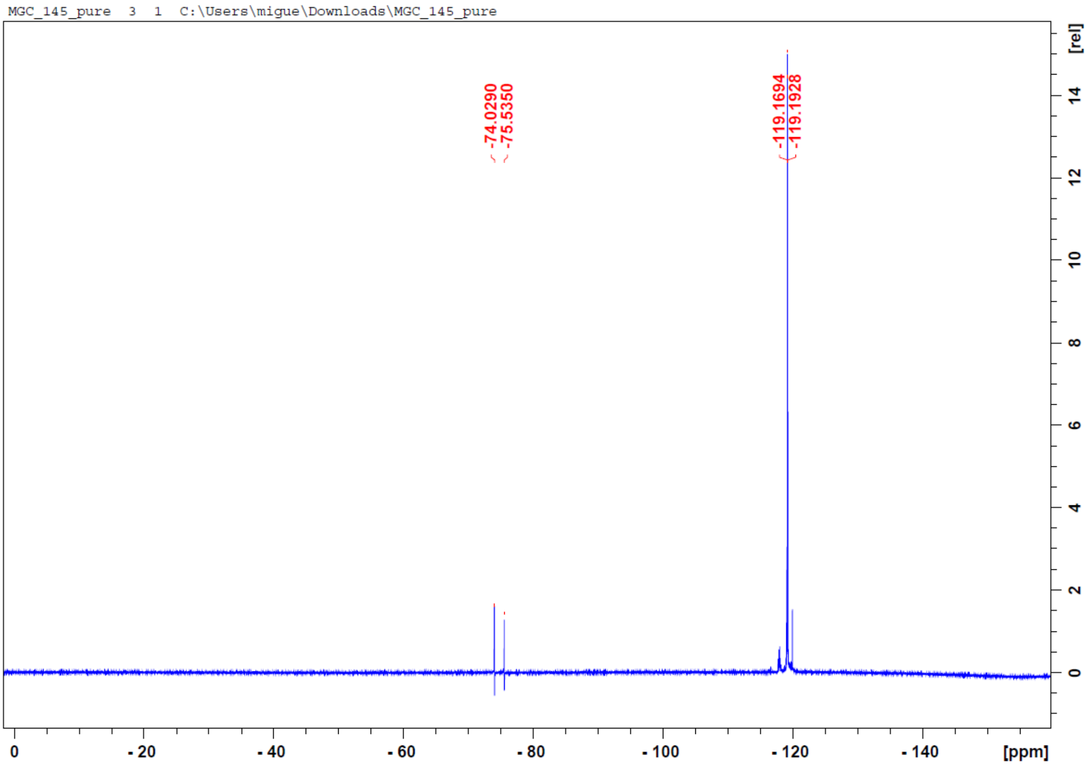

^{19}F-NMR (470 MHz, MeOD-d_4) of Boc-duple[1,2] (**22**)

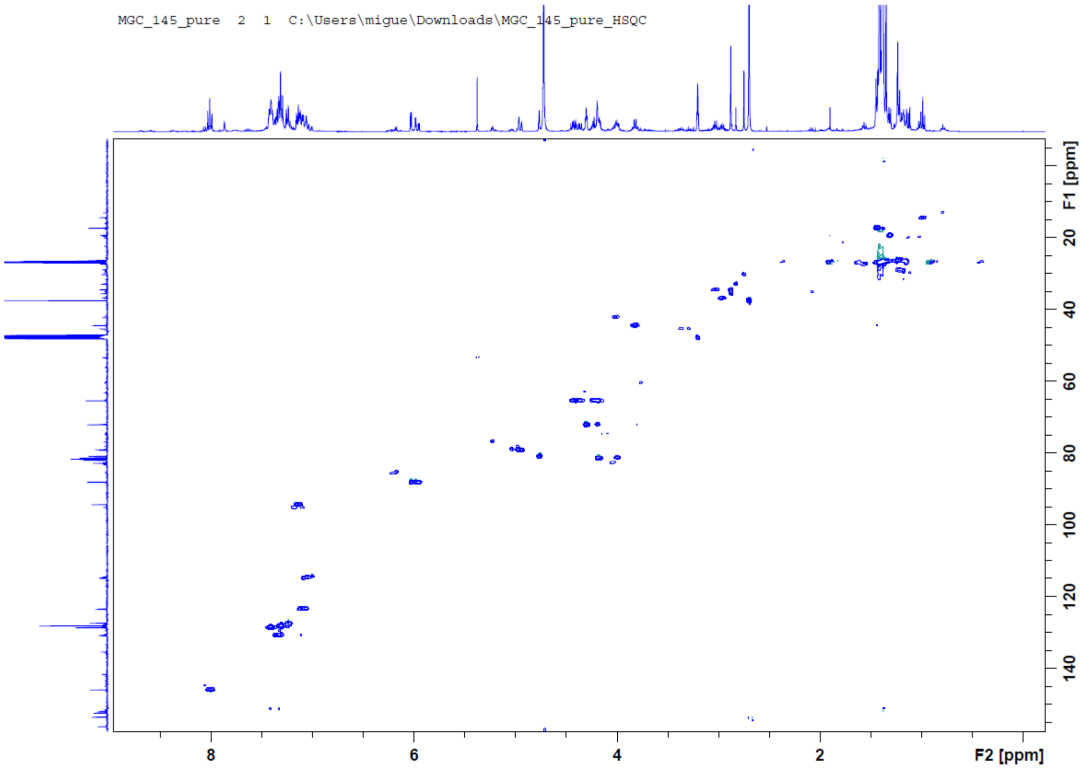

Bidimensional HSQC-experiment of Boc-duple[1,2] (**22**)

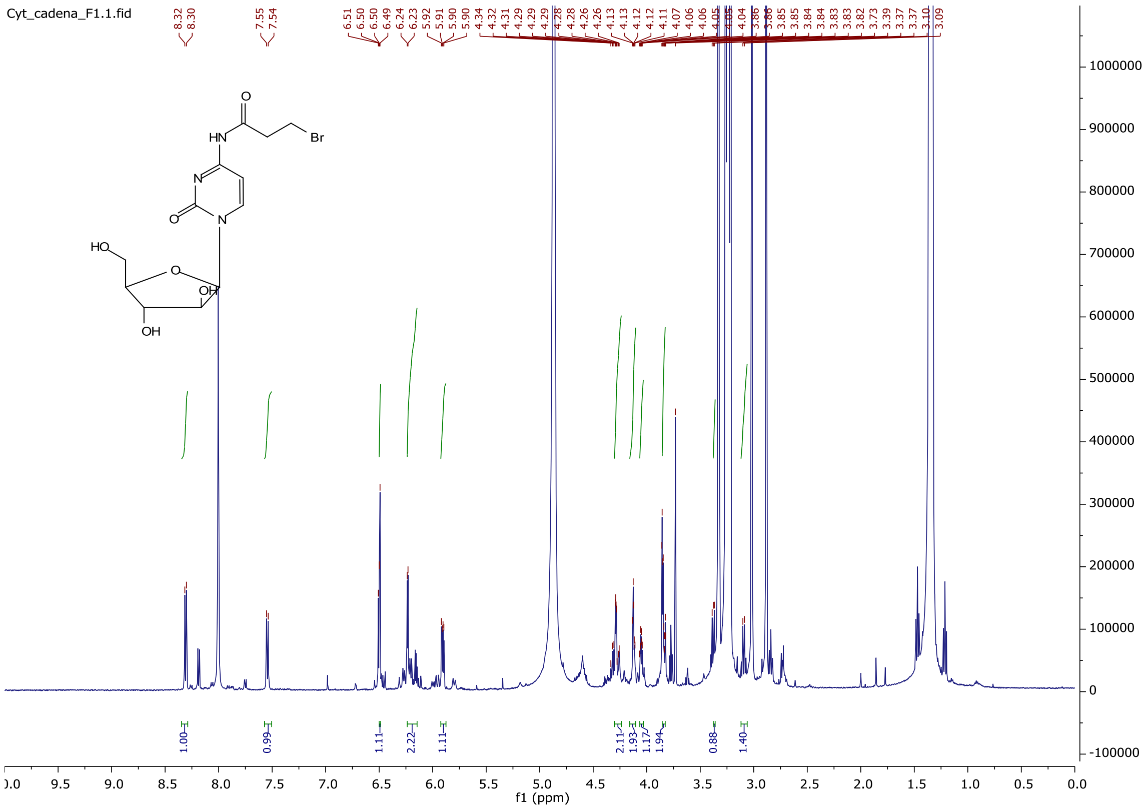

^{1}H-NMR (500 MHz, MeOD-d_4) of compound **20**

Chemical nomenclature of our 16-duples

[Compound **4**]: Ibuprofen bond in position 4 = $duple^{4,1}$

[Compound **5**]: Flurbiprofen bond in position 4 = $duple^{4,2}$

[Compound **6**]: Folic acid bond in position 4 = $duple^{4,3}$

[Compound **7**]: Sulfasalazine bond in position 4 = $duple^{4,4}$

[Compound **8**]: Bortezomib bond in position 2 and 3 = $duple^{2\text{-}3,5}$

[Compound **9**]: Methotrexate bond in position 4 = $duple^{4,6}$

[Compound **10**]: Tazobactam bond in position 4 = $duple^{4,7}$

[Compound **11**]: Ciprofloxacine bond in position 4 = $duple^{4,8}$

[Compound **12**]: Dapsone bond in position 4 = $duple^{4,9}$

[Compound **13**]: Metoclopramide bond in position 4 = $duple^{4,10}$

[Compound **14**]: Hydroxychloroquine bond in position 4 = duple[4,11]

[Compound **15**]: Norfloxacine bond in position 4 = duple[4,12]

[Compound **16**]: Furosemide bond in position 4 = duple[4,13]

[Compound **17**]: Cilastatin bond in position 4 = duple[4,14]

[Compound **18**]: Alprostadil bond in position 4 = duple[4,15]

[Compound **19**]: Dexamethasone bond in position 4 = duple[4,16]

[Compound **23**]: Flurbiprofen bond in position 1 = duple[1,2]

Characterization of all synthesized compounds

Duple[4,1] (compound 4):
$C_{22}H_{29}N_3O_6$

^{1}H-NMR (500 MHz, $CDCl_3$) δ (ppm): 8.00 (d, *J* = 7.5 Hz, 1H, H-6), 7.30 (d, *J* = 6.9 Hz, 1H, H-5), 7.17 (d, *J* = 7.7 Hz, 2H, H-6’)), 7.06 (d, *J* = 7.9 Hz, 2H, H-7’), 5.96 (d, *J* = 3.6 Hz, 1H, H-7), 5.35 (m, 1H, -CON-*H*) 4.34 (s, 1H,H-8), 4.17 (s, 1H, C-9), 3.95 (s, 1H, H-10), 3.86 – 3.74 (m, 2H, H-11), 3.68 (m, 1H, H-2’), 2.39 (d, *J* = 7.1 Hz, 2H, H-11’), 1.79 (dt, *J* = 13.4, 6.7 Hz, 1H,), 1.43 (d, *J* = 6.9 Hz, 3H, H-3’), 0.85 (d, *J* = 6.5 Hz, 6H, H-13’);

^{13}C-NMR (126 MHz, CDCl3) δ (ppm): 174.75 (-CONH-), 162.22 (C-2), 155.72 (C-6), 146.67 (C-7), 141.17 (C-4’), 136.88 (C-7’), 129.75 (C-5’,6’), 127.38 (C-7’,8’), 96.54 (C-7), 87.97, 85.22, 77.29, 76.44, 75.07, 61.59, 47.46 (C-2’), 44.98 (C-10’), 29.71 (C-11’), 22.41 (C-13’), 18.36 (C-3’);

Exact mass: 431.2056; **MALDI-TOF-MS:** 454.428 $[M + Na]^+$

Duple[4,2] (compound 5):
$C_{24}H_{24}FN_3O_6$

^{1}H-NMR (500 MHz, DMSO-*d*$_6$) δ (ppm): 11.10 (s, 1H, CON-H), 8.08 (d, *J* = 7.5 Hz, 1H, C-6), 7.57-7.45 (m, 5H, -Ph), 7.40 (m, 1H, C-5’), 7.34-7.29 (m, 2H, C-6´, C-8’), 7.21 (m, 1H, C-5), 6.09-6.05 (m, 1H, C-7), 5.57-5.47 (m,-OH 2H), 5.42 (m, 1H, -OH), 5.07 (bs, 1H, -OH), 4.10-4.03 (m, 2H, C-2’, C-8), 3.93 (m, 1H, C-9), 3.84 (dt, *J* = 5.7 Hz, 3.3 Hz 1H, C-10), 3.62 (dd, *J* = 5.5, 3.2 Hz, 2H, C-11), 1.45 (d, *J* = 6.5 Hz, 3H, C-3’);

^{13}C-NMR (126 MHz, DMSO-*d*$_6$) δ (ppm): 174.61 (C-1`), 174.58, 162.72, 160.31-158.35 (*J* C-F = 246.2 Hz), 154.85, 147.46 (C-6), 135.29, 131.32, 131.29, 129.21, 129.19, 129.08, 128.31, 127.47-127.37 (*J* C-F = 10.7 Hz), 124.45, 124.43, 124.37, 124.34, 115.72, 115.68, 115.53, 115.49, 94.76 (C-5), 87.54 (C-7), 86.33, 86.27, 76.59, 76.54, 75.05 (C-8), 61.48 (C-11), 45.70 (C-2’), 18.35 (C-3’), 18.21;

Exact mass: 469.1649; **MALDI-TOF-MS**: 492.069 m/z $[M + Na]^+$

Duple[4,3] (compound 6):
$C_{28}H_{30}N_{10}O_{10}$

^{1}H-NMR (500 MHz, $CDCl_3$) δ (ppm): 8.59 (bs, 1H, - COO-**H**), 7.82 (d, *J* = 8.4 Hz, 1H), 7.62 (d, *J* = 8.3 Hz, 1H), 7.34 – 7.30 (m, 1H), 7.27 – 7.22 (m, 1H), 6.80 – 6.75 (m, 1H) , 6.37 (d, *J* = 17.3, 1H, H-6), 6.19 – 6.01 (m, 1H), 5.80 (d, *J* = 17.4 1H, H-5), 5.53 (m, 1H), 4.37 – 4.30 (m, 2H), 3.62 - 3.54 (m, 3H), 3.46-3.39 (m, 2H), 3.26 - 3.20 (m, 1H), 2.97 – 2.91 (m, 1H), 2.73-2.65 (m, 2H) 2.32 - 1.95 (bs, 3H);

^{13}C-NMR (126 MHz, DMSO) δ (ppm): 170.80, 169.47, 169.03, 161.45 149.06, 147.66, 132.44, 120.85, 112.94, 112.34, 60.22, 55.97, 55.84, 55.37, 49.06, 46.34, 46.31, 41.95, 35.21, 31.14, 26.41, 26.35, 24.14, 23.07, 21.21, 14.54;

Exact mass: 666.2146; **MALDI-TOF-MS:** 651.381 $[M-16\ (-NH_2)]^+$

Duple[4,4] (compound 7):
$C_{27}H_{25}N_7O_9S$

^{1}H NMR (500 MHz, DMSO-d_6) δ (ppm): 8.58 (d, *J* = 2.3 Hz, 1H), 8.32 (dd, *J* = 8.8, 2.4 Hz, 1H), 8.14 – 7.99 (m, 6H), 7.93 (d, *J* = 8.8 Hz, 1H),7.81 – 7.69 (m, 2H), 7.11 (d, *J* = 6.1 Hz, 1H, H-7), 6.77 (d, *J* = 10.5 Hz, 1H, H-6), 5.73 (d, *J* = 10.6 Hz, 1H, H-5), 5.71 (d, *J* = 4.5 Hz, 1H, -O**H**), 5.03 (d, *J* = 5.9 Hz, 1H, -O**H**), 4.98 – 4.94 (m, 2H, O**H**), 4.27 (m, 1H), 4.04 (m, 1H), 3.85 – 3.78 (m, 1H), 3.19 (m, 2H);

^{13}C NMR (126 MHz, DMSO-d_6) δ 169.55, 159.79, 149.69, 149.47, 138.93, 128.02, 127.97, 127.86, 127.02, 127.00, 125.71, 121.46, 116.45, 97.53, 86.27, 82.79, 73.72, 68.13, 59.52;

Exact mass: 623.1434; **MALDI-TOF-MS:** 528.767 $[M – 94]^+$

Duple[2-3,5] (compound 8):
$C_{28}H_{34}BN_7O_7$

^{1}H-NMR (500 MHz, $CDCl_3$) δ (ppm): 8.10 (s, 1H), 7.74 (d, *J* = 7.9 Hz, 1H), 7.47 (s, 1H),7.27 – 7.23 (m, 5H, -Ph) 7.05 (s, 1H), 6.97 (d, *J* = 8.7 Hz, 4H), 6.31 (dd, J = 17.0, 1.2 Hz, 1H, H-7), 6.08 (d, *J* = 10.4 Hz, 1H, H-6), 5.71 (dd, *J* = 10.4, 1H, H-5), 5.15 – 5.09 (m, 1H), 4.77 (t, *J* = 6.4 Hz, 1H), 4.38 – 4.22 (m, 3H), 3.86 (s, 2H), 2.02-2.00 (m, 2H) 1.78-1.76 (m, 1H) 0.95 – 0.81 (m, 6H, $-(CH_3)_2$);

^{13}C-NMR (126 MHz, MeOD-d_4) δ (ppm): 174.14 (-**C**ONH-) 169.87 (-**C**ONH-), 128.16, 128.14, 97.50 (C-7), 82.63, 63.73, 29.71, 29.33 $(-CH_3)_2$, 22.70;

Exact mass: 591.2613; **MALDI-TOF-MS:** 656.346 [M + Zn-H] $^{+}$ [Reference 1]

Duple[4,6] (compound 9):
$C_{29}H_{33}N_{11}O_9$

^{1}H-NMR (500 MHz, DMSO-*d*$_6$) δ (ppm): 11.33 (s, 1H,-COO-**H**), 8.73 (s, 1H), 8.19 (d, *J* = 7.5, 1H, H-6), 7.65 (d, *J* = 9.1, 1.5 Hz, 2H), 7.26 (d, *J* = 7.7 Hz, 1H, H-6), 6.92 (dd, *J* = 9.1, 1.8 Hz, 2H), 6.18 (dd, *J* = 3.9, 1.9 Hz, 1H. H-7), 5.59 (m, 2H, -O**H**), 5.49 (m, 1H, -O**H**), 4.91 (s, 2H, H-11'), 4.23 – 4.15 (m, 1H, H-4'), 4.06 – 3.94 (m, 2H, H-8, H-9), 3.95 (dt, *J* = 5.6, 3.0 Hz, 1H, H-10) 3.73- (t, J = 5.6 Hz, 2H,H-11), 3.37 (s, 3H,-N-C**H$_3$**), 2.75 (m, 2H), 2.48 (m, 2H);

^{13}C-NMR (126 MHz, DMSO-*d*$_6$) δ (ppm): 169.69, 161.54, 159.46, 154.80, 148.23, 135.00, 133.94, 130.05, 129.20, 120.47, 120.31, 87.64, 75.06, 65.17, 61.52, 51.60, 30.43, 26.15;

Exact mass: 679.2463; **MALDI-TOF-MS:** 702.417 m/z [M + Na]$^+$, 684.433 m/z [M - 17 (-NH_3) + Na]$^+$

Duple[4,8] (compound 11): [Reference 59 in the maintext]
$C_{26}H_{29}FN_6O_7$

^{1}H-NMR (500 MHz, DMSO-*d*$_6$) δ (ppm): 9.24 (s, 2H), 8.59 – 8.45 (m, 3H), 8.28 – 8.20 (m, 1H), 8.13 (s, 1H), 8.10 (s, 1H), 8.07 (s, 1H), 7.79 (m, 1H), 6.98 (m, 1H), 5.76 (s, 1H), 5.61 (s, 1H), 5.23 (m, 1H), 5.11 (m, 1H), 5.00 (m, 2H), 4.93 (m, 1H), 4.81 (m, 1H), 4.44 (s, 1H), 3.61 (m, 8H), 3.22 (m, 4H), 3.19 (m, 4H), 3.13 (m, 8H), 2.75 (s, 2H);

^{13}C-NMR (126 MHz, DMSO-*d*$_6$) δ (ppm): 172.36, 170.20, 167.75, 166.02, 157.43-155.01 (C-F, *J* C-F = 302 Hz), 139.52, 134.00, 133.88, 133.79, 129.97, 127.95, 127.51-127.20 (C-F, *J* C-F = 38.8 Hz), 107.43, 67.49, 66.45, 65.64, 62.32, 60.83, 53.81, 42.07, 34.27, 25.60, 18.46, 17.18, 12.70;

Exact mass: 556.2082; **MALDI-TOF-MS:** 557.534 m/z [M + H]$^+$

Compound 20:

^{1}H-NMR (500 MHz, MeOD-*d*$_4$) δ (ppm): 8.31 (d, *J* = 7.5 Hz, 1H), 7.54 (d, *J* = 7.5 Hz, 1H), 6.56 – 6.43 (m, 1H), 6.23 (d, *J* = 3.8 Hz, 2H), 5.91 (m, 1H), 4.33 – 4.23 (m, 2H), 4.12 (m, 1H), 4.05 (m, 1H), 3.87 – 3.81 (m, 2H), 3.39 – 3.36 (m, 1H), 3.09 (m, 1H);

Boc-Duple[1,2] (compound 22):
$C_{29}H_{32}FN_3O_8$

^{1}H-NMR (500 MHz, MeOD-*d4*) δ (ppm): 7.74 (d, *J* = 7.6 Hz, 1H), 7.49 – 7.22 (m, 10H), 6.08 (dd, *J* = 10.4, 3.5 Hz, 1H), 4.49 (ddd, *J* = 11.5, 7.9, 3.3 Hz, 1H), 4.21 – 4.12 (m, 3H), 4.10 – 4.03 (m, 1H), 3.93 (dt, *J* = 13.3, 2.2 Hz, 1H), 3.82 (qd, *J* = 7.2, 2.5 Hz, 1H), 1.43 (s, 3H), 1.41 (s, 9H);

^{13}C-NMR (126 MHz, MeOD-*d4*) δ (ppm): 173.09, 173.02, 163.72, 156.19-153.54 (*J* C-F = 333.9 Hz), 153.48, 152.42, 152.03, 145.99, 128.62-128.57 (*J* C-F = 5.04 Hz), 128.12, 127.40, 94.37, 88.09, 82.82, 81.96, 81.70, 81.36, 80.89, 72.08, 65.41, 37.51, 26.97, 26.65, 26.55, 17.34, 14.38;

^{19}F-NMR (470 MHz, MeOD-*d4*) δ (ppm): -119.17

Exact mass: 569.2173; **MALDI-TOF-MS:** 590.946 m/z $[M + Na]^+$

TPSO-Cyt (Intermediate I):

^{1}H-NMR (500 MHz, MeOD-*d4*) δ (ppm): 7.83 – 7.63 (m, 5H), 7.54 – 7.34 (m, 6H), 6.19 (d, *J* = 4.7 Hz, 1H), 5.64 (d, *J* = 7.5 Hz, 1H), 4.22 (dd, *J* = 4.6, 3.6 Hz, 1H), 4.17 (t, *J* = 3.9 Hz, 1H), 3.96 (dd, *J* = 9.9, 3.4 Hz, 1H), 3.93 – 3.86 (m, 2H), 1.06 (s, 9H);

^{13}C-NMR (126 MHz, MeOD-*d4*) δ (ppm): 166.15, 157.00, 142.85, 135.37, 135.27, 133.06, 132.79, 129.68, 129.63, 127.53, 127.52, 93.49, 86.25, 83.86, 75.81, 75.70, 62.98, 25.96, 18.73;

TPSO-Duple[2-3,5] (Intermediate II):
$C_{44}H_{52}BN_7O_7Si$

^{1}H-NMR (500 MHz, MeOD-*d4*) δ (ppm): 9.15 (d, *J* = 1.5 Hz, 1H), 8.77 (d, *J* = 2.5 Hz, 1H), 8.70 – 8.61 (m, 1H), 7.75 – 7.61 (m, 5H), 7.47 – 7.34 (m, 6H), 7.28 – 7.18 (m, 6H), 6.19 (d, *J* = 4.6 Hz, 1H), 5.65 (d, *J* = 7.5 Hz, 1H), 5.01 (t, *J* = 7.6 Hz, 1H), 4.26 – 4.21 (m, 1H), 4.17 (d, *J* = 4.0 Hz, 1H), 4.00 – 3.83 (m, 3H), 3.23 (t, *J* = 7.9 Hz, 2H), 2.66 (t, *J* = 7.6 Hz, 1H), 1.39 (dt, *J* = 13.4, 6.7 Hz, 1H), 1.17 (dd, *J* = 8.3, 6.4 Hz, 2H), 1.06 (s, 9H), 0.82 (dd, *J* = 6.6, 5.8 Hz, 8H);

^{13}C-NMR (126 MHz, MeOD-*d4*) δ (ppm): 175.50, 166.00, 163.77, 156.83, 144.23, 143.44, 143.43, 142.91, 135.65, 135.37, 133.05, 132.78, 129.69, 129.65, 129.15, 128.34, 127.53, 126.89, 93.55, 86.28, 83.90, 75.68, 62.99, 51.46, 39.48, 37.23, 25.98, 25.30, 22.41, 20.72, 18.74;

Exact mass: 829.3791 **MALDI-TOF-MS:** 831.087 $[M+H]^+$

MALDI-TOF Analyses of synthesized duples

MALDI-TOF-MS. 454.428 [M + Na]$^+$

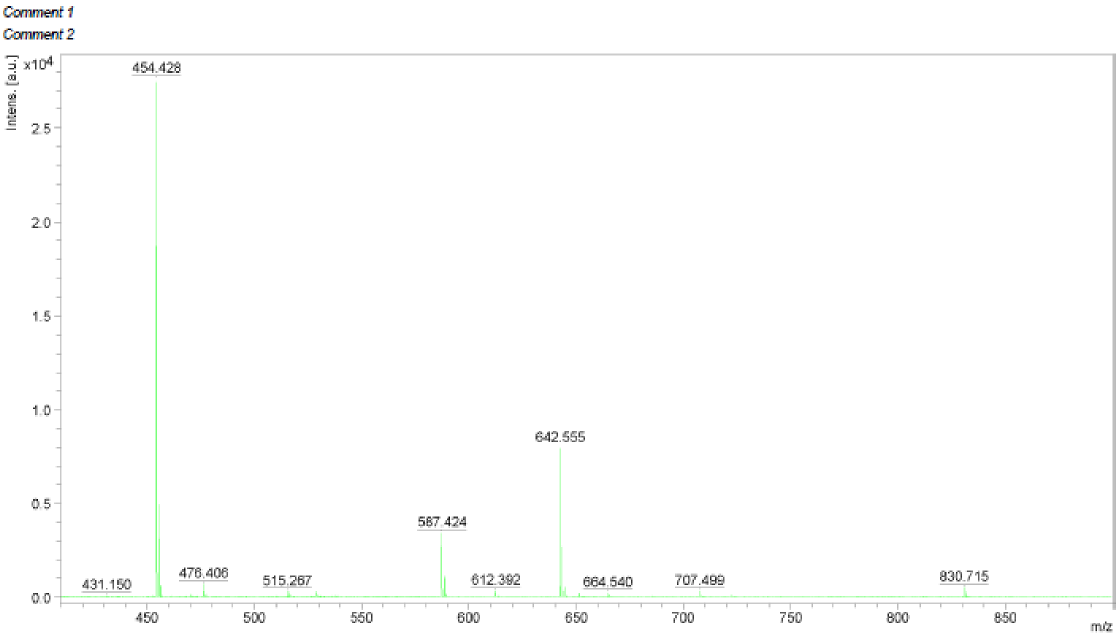

Mass spectrometry of duple[4,1]

MALDI-TOF-MS: 492.069 m/z [M + Na]$^+$

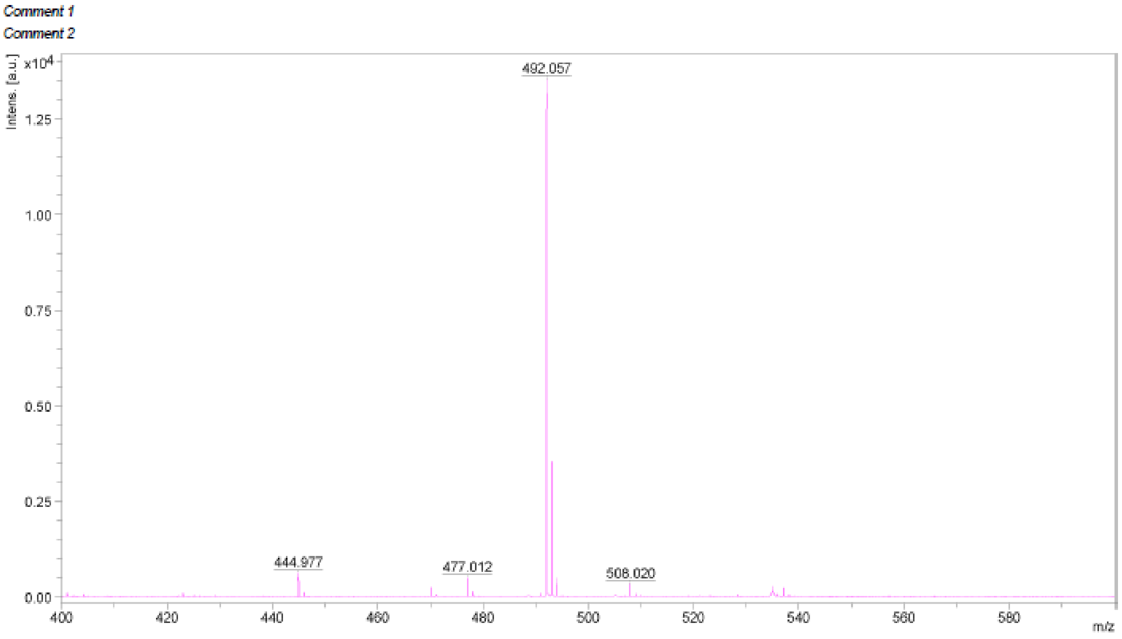

Mass spectrometry of duple[4,2]

MALDI-TOF-MS: 651.381 [M-16 (-NH_2)]$^+$

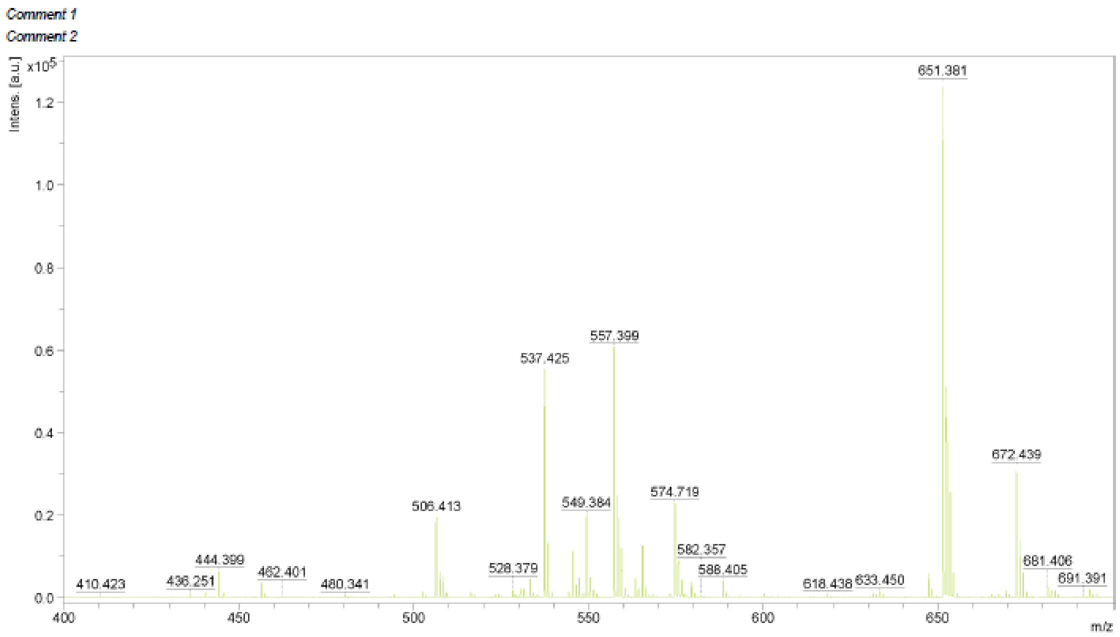

Mass spectrometry of duple[4,3]

MALDI-TOF-MS: 528.767 [M – 94]$^+$

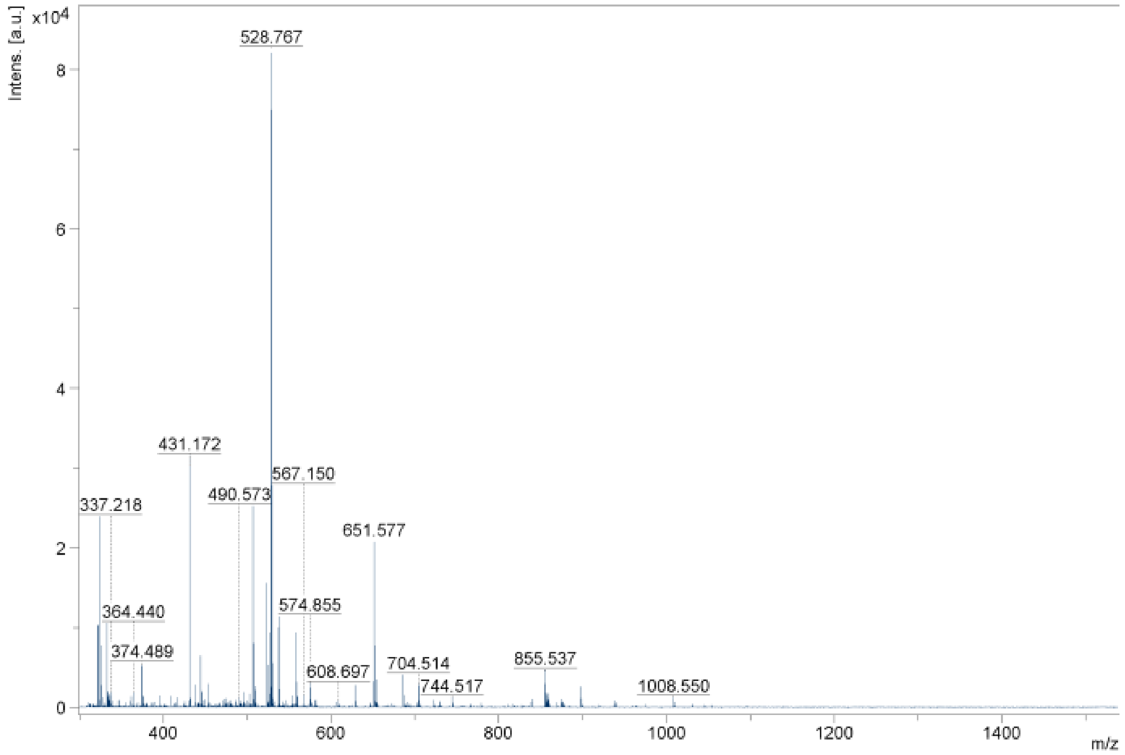

Mass spectrometry of duple[4,4]

MALDI-TOF-MS: 656.346 [M + Zn-H]$^+$ [Reference 58 in the maintext]

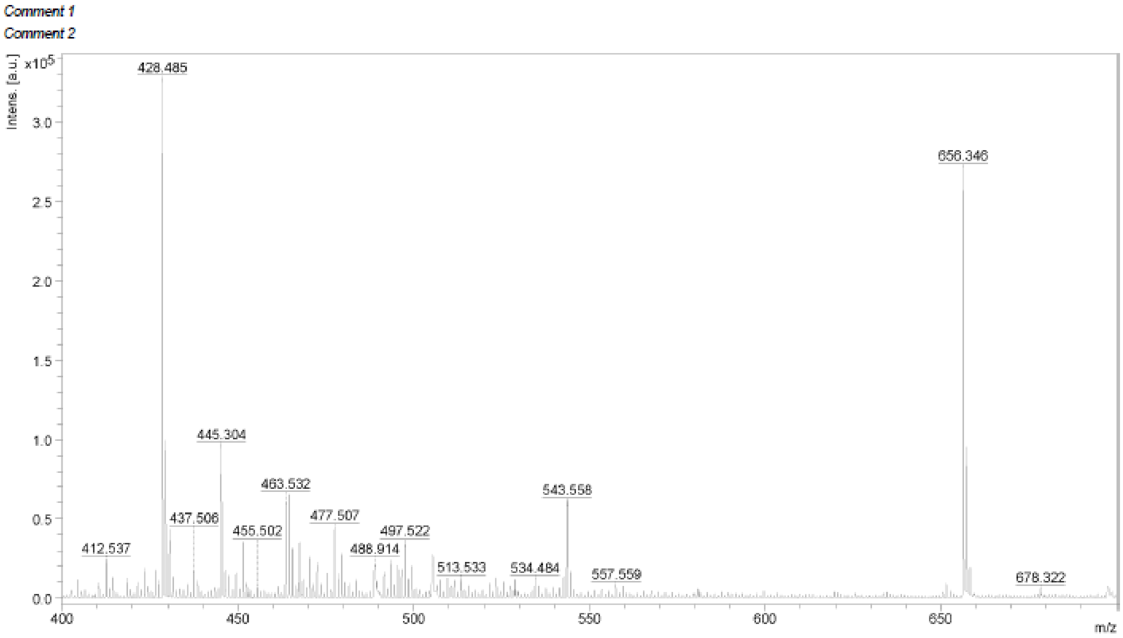

Mass spectrometry of duple[2,3,5]

MALDI-TOF-MS: 702.417 m/z [M + Na] $^+$, 684.433 m/z [M - 17 (-NH_3) + Na]$^+$

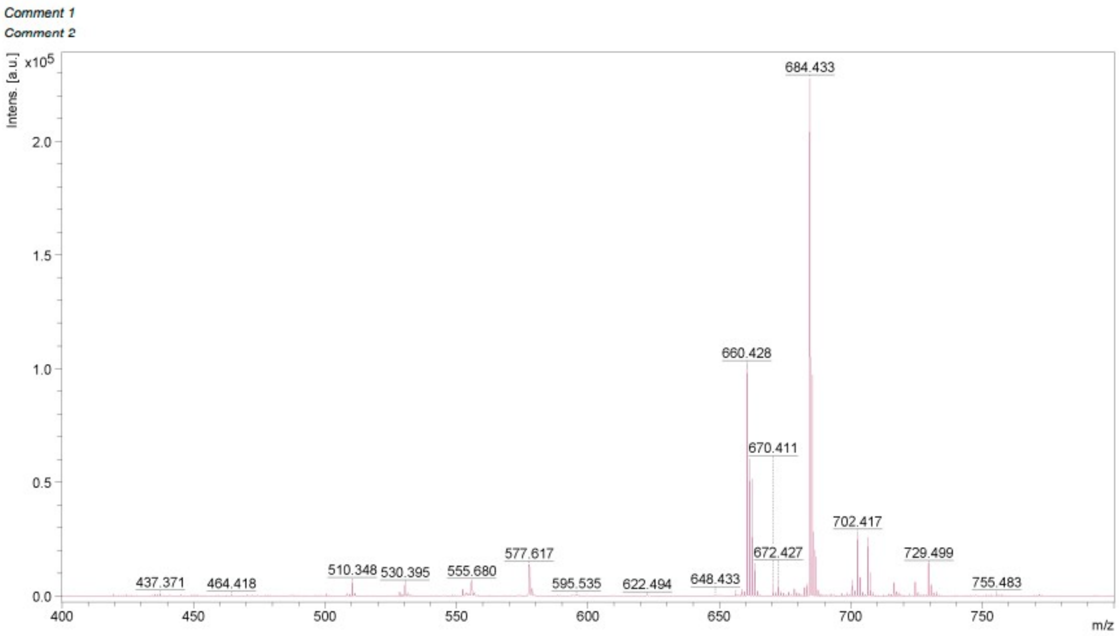

Mass spectrometry of duple[4,6]

MALDI-TOF-MS: 557.534 m/z [M + H]$^+$

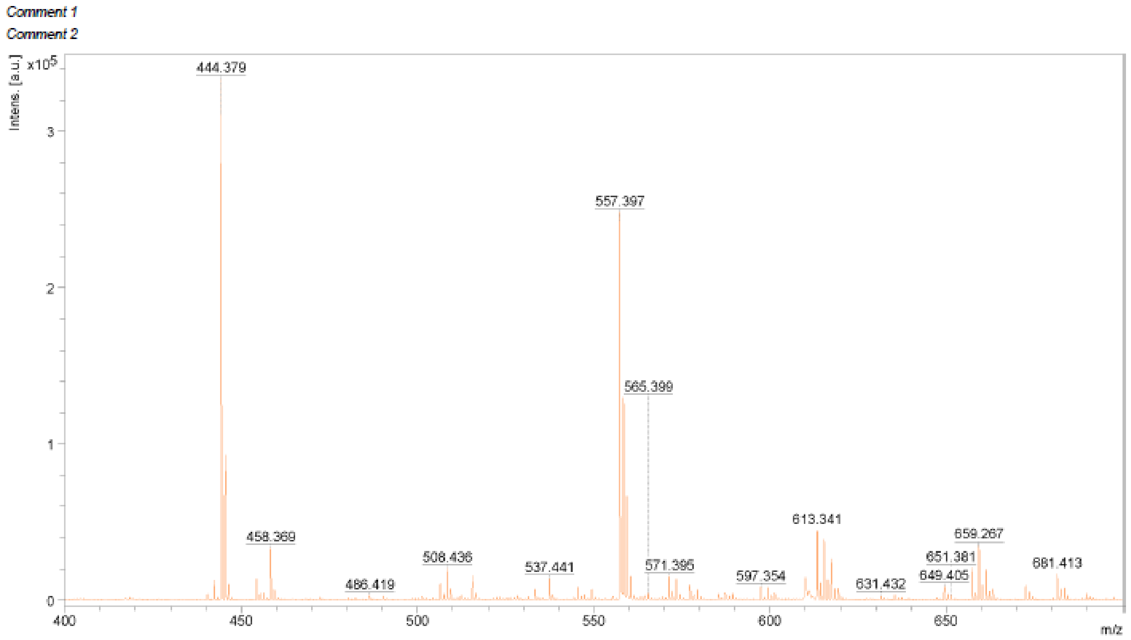

Mass spectrometry of duple[4,8]

MALDI-TOF-MS: 831.087 [M+H]$^+$

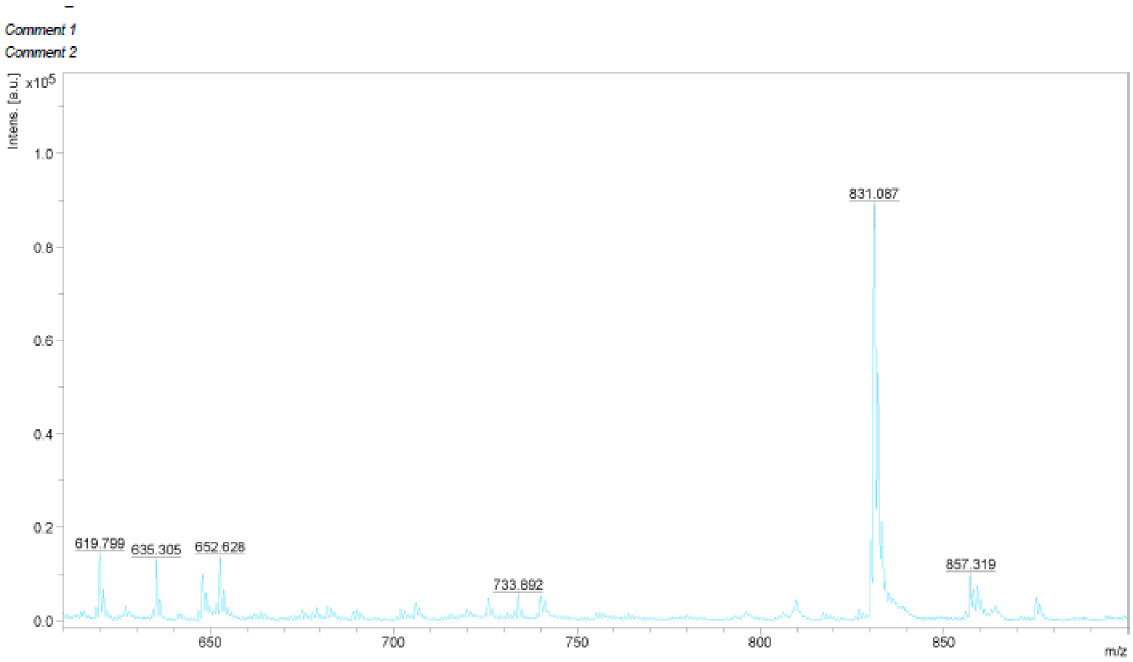

Mass spectrometry of TPSO-duple[2-3,5] (Intermediate **II**)

MALDI-TOF-MS: 590.946 [M + Na]+

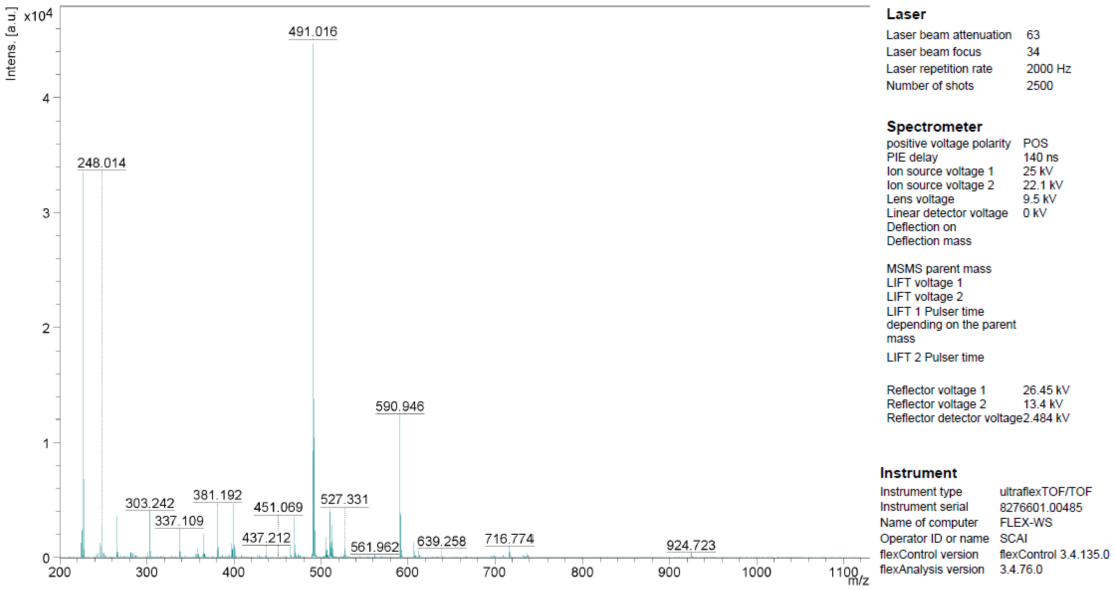

Mass spectrometry of Boc-duple[1,2]

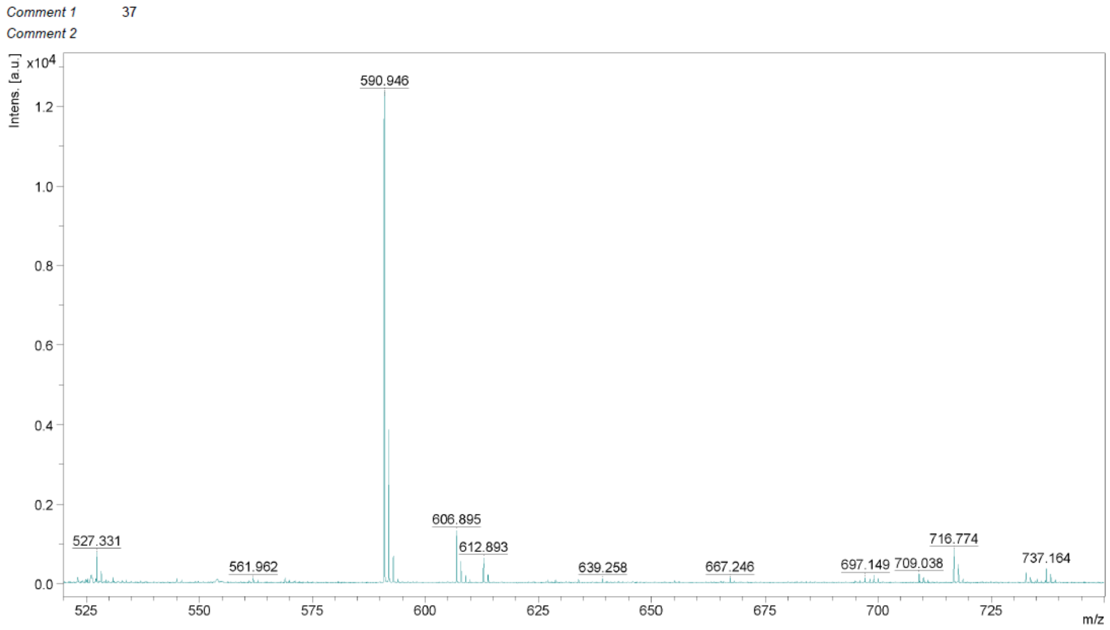

Mass spectrometry of Boc-duple[1,2] (scaled up)
